# Iodine(III)-mediated halogenations of acyclic monoterpenoids

**DOI:** 10.3762/bjoc.14.96

**Published:** 2018-05-18

**Authors:** Laure Peilleron, Tatyana D Grayfer, Joëlle Dubois, Robert H Dodd, Kevin Cariou

**Affiliations:** 1 Institut de Chimie des Substances Naturelles, CNRS UPR 2301, Université Paris-Sud, Université Paris-Saclay, Avenue de la Terrasse, 91198 Gif-sur-Yvette, France

**Keywords:** halogenation, hypervalent iodine, monoterpenes

## Abstract

Five different halofunctionalizations of acyclic monoterpenoids were performed using a combination of a hypervalent iodine(III) reagent and a halide salt. In this manner, the dibromination, the bromo(trifluoro)acetoxylation, the bromohydroxylation, the iodo(trifluoro)acetoxylation or the ene-type chlorination of the distal trisubstituted double bond occurred with excellent selectivity and moderate to good yields.

## Introduction

**Figure 1:**
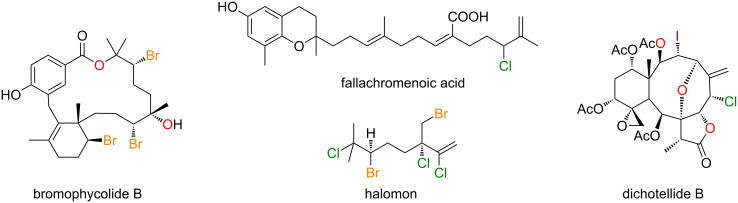
Halogenated terpenoids from natural sources.

In nature, mostly in marine environments, halogenated compounds are produced by means of various enzymes that rely on widely available halides as feedstock [1]. These halogenases can perform an extremely wide array of electrophilic halogenations on a myriad of substrates with exquisite chemo-, stereo- and enantioselectivities that remain extremely challenging to rival for the chemist. When applied to terpenic feedstock, they give birth to a variety of molecular scaffolds, from the mono-chlorinated linear chain of fallachromenoic acid [2], to the pentahalogenated halomon skeleton [3] and encompassing many intricate polycyclic and macrocyclic structures with complex vicinal oxygen/halogen patterns, such as bromophycolide B [4] and dichotellide B, which contains both iodine and chlorine atoms (Figure 1) [5].

**Scheme 1:**
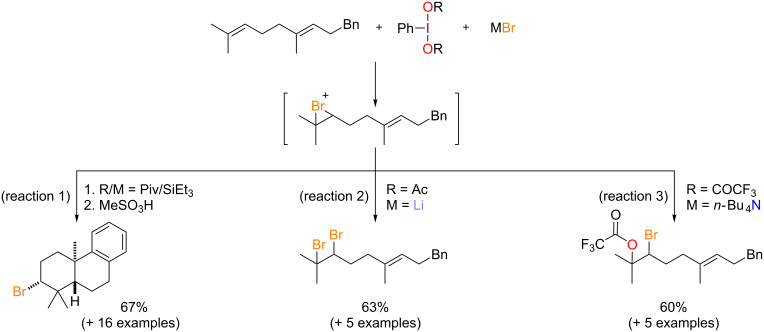
Previously developed bromo-functionalizations of polyprenoids using iodine(III) reagents.

This structural richness has fuelled the development of many synthetic strategies that take inspiration from these enzymatic machineries. A key aspect is to be able to mildly oxidize the halides into halenium equivalents in order to promote halogenations with increased selectivity. In this regard, hypervalent iodine reagents [6] have emerged as particularly versatile mediators [7–10]. We have shown that electrophilic halogenations [11–13], or pseudohalogenations [14] can be triggered by combining an iodine(III) derivative with a suitable halide salt. In particular, the chemoselectivity of the reaction can be finely tuned by adjusting several parameters, such as the nature of the halide as well as of the iodine(III) ligands and the halide counterion [15–16]. In the case of polyprenoids, we mostly devoted our efforts to achieve the bromocarbocyclization of aryl-geranyl derivatives using a combination of iodine(III) oxidant and a bromide source. In this fashion, the reaction of homogeranylbenzene with bis(*tert*-butylcarbonyloxy)iodobenzene and triethylsilyl bromide, followed by acidic treatment led to a tricyclic brominated adduct (Scheme 1, reaction 1). Yet in the course of our study we also showed that the reactivity of the key bridged bromonium intermediate could also be steered towards non-cyclizing vicinal difunctionalizations using slightly different combinations of a (diacyloxyiodo)arene and a bromide salt. Indeed, (diacetoxy)iodobenzene (DIB) and lithium bromide yield a dibromo adduct (Scheme 1, reaction 2), whereas a combination of (bis(trifluoroacetoxy)iodo)benzene (PIFA) and tetra-*n*-butylammonium bromide (TBAB) gives bromo(trifluoro)acetoxylated **3a** (Scheme 1, reaction 3) [16].

**Figure 2:**
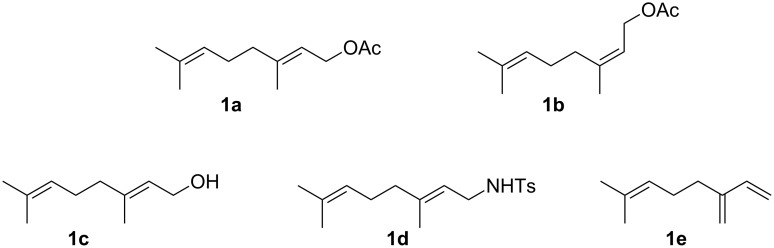
Selected monoterpenoids used in this study.

We then decided to further explore the synthetic potential of the latter two vicinal difunctionalizations of terpenoids by not only expanding their substrate scope but also by trying to achieve other bromo-oxylations as well as analogous chloro- and/or iodofunctionalizations of linear terpenoids. For this purpose, five monoterpene derivatives – bearing various functional groups (protected and free alcohols, amine, *E* and *Z* olefins, diene moiety) aimed at probing potential chemo-, regio- and stereoselectivity issues – were selected: geranyl acetate (**1a**), neryl acetate (**1b**), geraniol (**1c**), *N*-tosylgeranylamine (**1d**) and myrcene (**1e**, Figure 2).

## Results and Discussion

### Optimizations

In order to carry out the exploration of the various halogenations that could be performed, geranyl acetate (**1a**) was chosen as the model substrate. In our study on terpenoids [16], the reaction conditions for the dibromination of the distal double bond were easily established from our previous study on the bromination of enamides [11]. Thus, using a slight excess of DIB along with a two-fold amount of lithium bromide at 0 °C in dry acetonitrile rapidly yielded dibromo adduct **2a** in 91% yield (Table 1, entry 1). Switching the reaction conditions to bromo(trifluoro)acetoxylation requires the use of a PIFA/TBAB combination in a 1:1 ratio with slow addition of the latter to the reaction mixture thereby preventing the formation of **2a**. In this fashion, bromo(trifluoro)acetoxy adduct **3a** was obtained in 77% yield (Table 1, entry 2). The reaction course can also be modified by changing the solvent. For instance, the complementary hydroxybromination reaction, giving **4a** in 59% yield, was achievable if a mixture of acetonitrile and water was used as the solvent (Table 1, entry 3). In order to suppress the observed formation of minor amounts of **2a**, the reaction temperature was lowered to −10 °C and the amount of lithium bromide, which was added dropwise as an aqueous solution, was diminished to 1.3 equivalents. By doing so, both the selectivity and the yield of **4a** were improved though full conversion was not attained (Table 1, entry 4). Keeping the same procedure, complete reaction was nevertheless achieved by slightly increasing the amount of each reagent thus efficiently giving bromohydroxylated adduct **4a** in 70% yield (Table 1, entry 5). Since the use of water as the sole solvent was not possible because of solubility issues, the reaction was attempted in ethanol. In this case the analogous bromoethoxylated adduct **4a’** could be isolated in 68% yield, albeit along with 25% of **2a** (Table 1, entry 6). Turning our attention to iodination, we first used the combination of PIFA and KI that had given the best results with enamides [13]. Thus, iodo(trifluoro)acetoxylated adduct **5a** was obtained in 70% yield (Table 1, entry 7). Interestingly, no traces of the diiodo compound were observed even if the hypervalent iodine(III) reagent was slowly added. Transposing the previously optimized bromo(trifluoro)acetoxylation conditions but using TBAI instead of KI did not improve the yield and reaction times and excess reagents were required to reach completion (Table 1, entry 8). Using directly 1.5 equivalents of both PIFA and TBAI did not suffice to improve the yield, whether in acetonitrile or in dichloromethane (Table 1, entries 9 and 10). When chlorination was attempted with a combination of DIB and iron(III) chloride in a 1.2:0.8 ratio (i.e., a 1:1 OAc/Cl ratio [12]) in acetonitrile, allylic chloride **6a** [17] was obtained with a moderate 45% yield (Table 1, entry 11). Switching to a combination of PIFA and TBACl (Table 1, entry 12) did not change the course of the reaction towards chloro(trifluoro)acetoxylation and, while the yield was moderate in acetonitrile, it was greatly improved in dichloromethane [18], affording **6a** in 85% yield (Table 1, entry 13).

**Table 1:** Optimization of the reactions conditions.

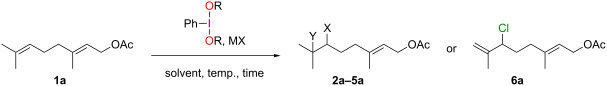						

entry	R (x equiv)	MX (y equiv)	solvent	temp.	time	N°, Y, X (yield %)^a^

1	Ac (1.2)	LiBr (2.4)	MeCN	0 °C	5 min	**2a**, Br, Br (91)
2	C(O)CF_3_ (1.1)	*n*-Bu_4_NBr^b^ (1.2)	MeCN	0 °C	15 min^c^	**3a**, OCOCF_3_, Br (77)
3	Ac (1.2)	LiBr (2.4)	MeCN/H_2_O	rt	5 min	**4a**, OH, Br (59)^d^
4	Ac (1.2)	LiBr^e^ (1.3)	MeCN/H_2_O	−10 °C	15 min^c^	**4a**, OH, Br (65)^f^
5	Ac (1.4)	LiBr^e^ (1.6)	MeCN/H_2_O	−10 °C	15 min^c^	**4a**, OH, Br (70)
6	Ac (1.2)	LiBr (1.2)	Et**O**H	−10 °C	105 min	**4a’**, OEt, Br (68)^g^
7	C(O)CF_3_ (1.5)^h^	KI (2.4)	MeCN	0 °C	20 min	**5a**, OCOCF_3_, I (70)
8	C(O)CF_3_ (1.3)^i^	*n*-Bu_4_NI^b^ (1.5)^i^	MeCN	0 °C	100 min	**5a**, OCOCF_3_, I (44)
9	C(O)CF_3_ (1.5)	*n*-Bu_4_NI^b^ (1.5)	MeCN	0 °C	20 min	**5a**, OCOCF_3_, I (60)^j^
10	C(O)CF_3_ (1.5)	*n*-Bu_4_NI^b^ (1.5)	CH_2_Cl_2_	0 °C	20 min	**5a**, OCOCF_3_, I (63)
11	Ac (1.2)	FeCl_3_ (0.8)	MeCN	rt	5 min	**6a** (45)
12	C(O)CF_3_ (1.2)	*n*-Bu_4_NCl^b^ (1.5)	MeCN	0 °C	15 min^c^	**6a** (36)
13	C(O)CF_3_ (1.2)	*n*-Bu_4_NCl^b^ (1.5)	CH_2_Cl_2_	0 °C	15 min^c^	**6a** (85)

^a^Isolated yields; ^b^slow addition of a 0.1 M solution of the TBA salt; ^c^5 min of addition followed by 10 min of stirring; ^d^along with 6% of **2a**; ^e^slow addition of a 0.1 M aqueous solution of LiBr; ^f^full conversion was not reached; ^g^along with 25% of **2a**; ^h^slow addition of a 0.1 M solution of PIFA; ^i^initially 1.1 equiv of PIFA and 1.2 equiv of TBAI, followed by 0.2 equiv of PIFA and 0.3 equiv of TBAI to reach completion; ^j^along with 20% of **1a**.

At this stage, we chose to discard the bromoethoxylation reaction which led to mixtures of compounds, leaving five optimized transformations (dibromination, bromo(trifluoro)acetoxylation, bromohydroxylation, iodo(trifluoro)acetoxylation and ene-type chlorination) which were applied to substrates **1b**–**e** in order to explore their scope.

### Bromination

#### Dibromination

**Scheme 2:**
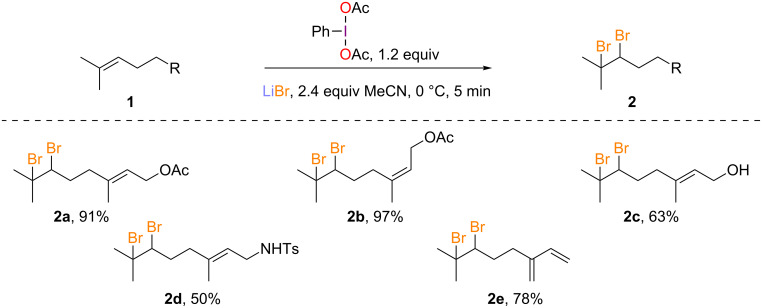
Dibromination of acyclic monoterpenoids.

Application of the dibromination protocol to neryl acetate (**1b**) proceeded smoothly and the desired compound **2b** was obtained in nearly quantitative yield without any detectable reaction on the *Z* double bond (Scheme 2). Despite the potential sensitivity of the alcohol and the amine functions, both geraniol (**1c**) and protected geranylamine **1d** gave the dibromo adduct **2c** [16] and **2d** in good yields. Finally, the reaction was performed on myrcene (**1e**), providing **2e** in 78% yield and leaving the diene moiety untouched.

#### Bromo(trifluoro)acetoxylation

**Scheme 3:**
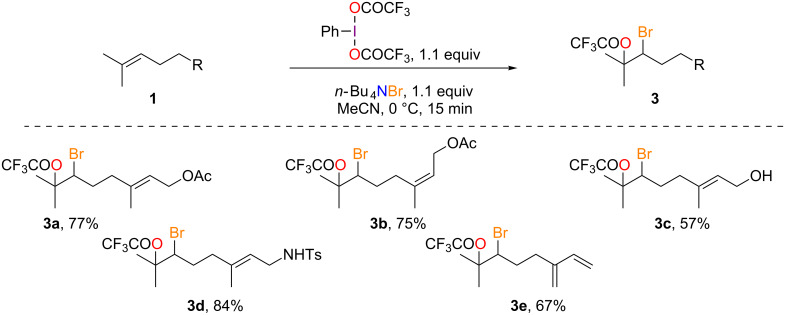
Bromo(trifluoro)acetoxylation of acyclic monoterpenoids.

The success of the bromo(trifluoro)acetoxylation lies in the reverse addition protocol whereby the tetrabutylammonium bromide is slowly added to the suspension of the terpenoid and PIFA at 0 °C. In this manner, good to excellent yields (57–84%) were obtained for the formation of *Z*-derivative **3b**, alcohol **3c** [16], tosylamine **3d** and diene **3e** (Scheme 3). Once again, the selectivity is excellent as no reactions with the other double bonds or with the aryl ring [19] are observed and only one regioisomer is formed in all cases.

#### Bromohydroxylation

**Scheme 4:**
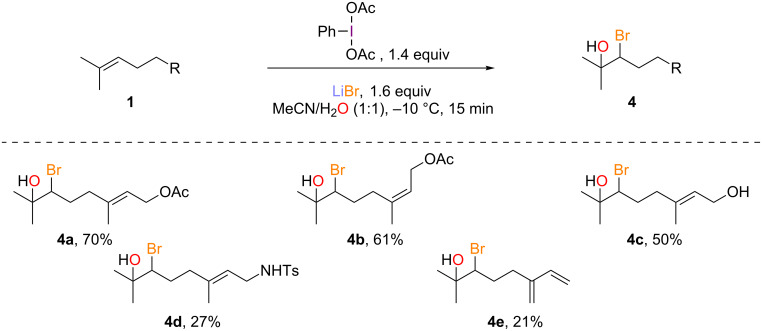
Bromohydroxylation of acyclic monoterpenoids.

Although the trifluoroacetoxy group can be readily cleaved [16], having a direct access to the corresponding bromohydrins would still be desirable especially since selective deprotection in the presence of another ester would presumably be difficult to achieve. By using water as the co-solvent, bromohydrins **4b–e** were directly prepared from the corresponding starting material, albeit with moderate yields in the case of *N*-tosylgeranylamine (**1d**) and myrcene (**1e**, Scheme 4).

### Iodo(trifluoro)acetoxylation

**Scheme 5:**
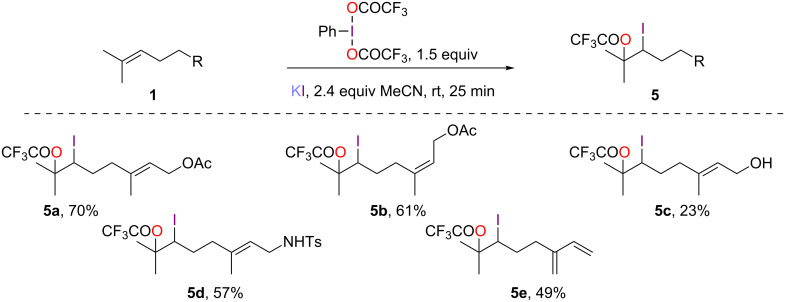
Iodo(trifluoro)acetoxylation of acyclic monoterpenoids.

In an analogous fashion to the above-described bromo(trifluoro)acetoxylation, the iodo(trifluoro)acetoxylation proceeded with both good yields (49–70%) and chemo-selectivities for geranyl and neryl acetates (**1a**,**b**), geranylamine **1d** and myrcene (**1e**, Scheme 5). However, the adducts were quite sensitive and needed to be manipulated with care (low temperature storage) to avoid rapid decomposition. Nevertheless, this was particularly the case for alcohol **5c** which could only be isolated with a 23% yield. This mild protocol offers a complementary alternative to the use of iodine monoacetate [20] or NIS [21] and does not require the use of a strong oxidant such as IBX [22]. Compared to the standard procedure for the preparation of acetoxyhypohalites that requires the use of expensive and potentially toxic silver salts [23], our method offers a more practical alternative. It also differs from the more user-friendly protocols that rely on Oxone^®^ [20], DIB [24], or PIFA [25] since the iodide source is an iodide salt and not molecular iodine.

### Chlorination

**Scheme 6:**
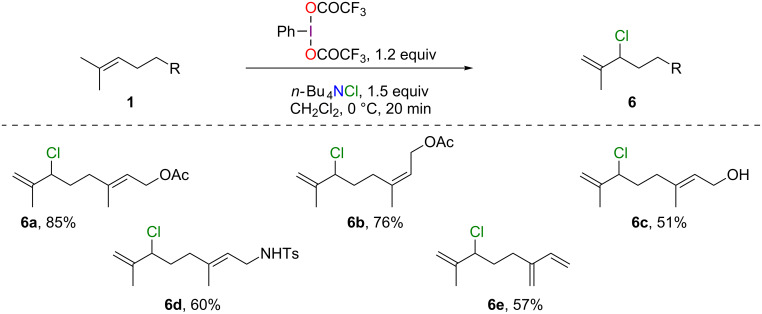
Chlorination of acyclic monoterpenoids.

In the case of chlorination, we have yet to observe adducts arising from the vicinal difunctionalization of the double bond and, in accordance with the literature [13], ene-type products **6** were obtained (Scheme 6). The reaction performed equally well with acetates (**6a**,**b**), alcohol (**6c**), amine (**6d**) and myrcene (**6e**). Once again, excellent chemo- and regioselectivities and good yields (51–85%) were witnessed.

### Mechanism proposal and control experiments

**Scheme 7:**
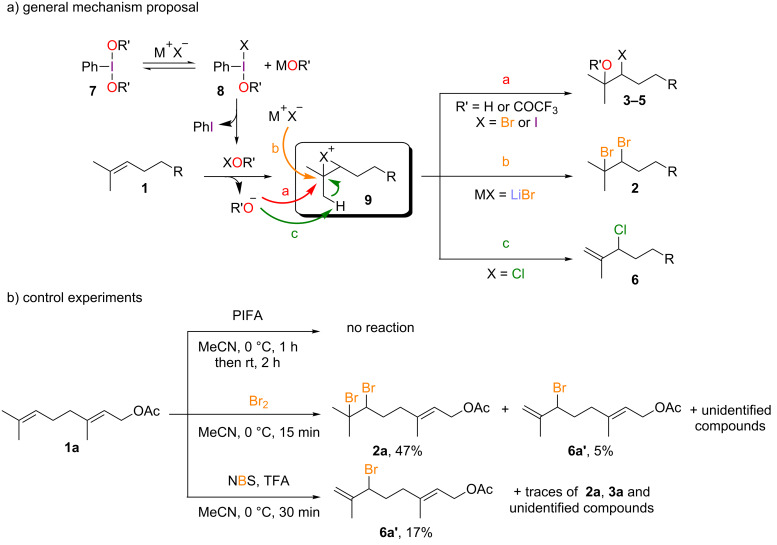
General mechanism proposal for the formation of **2**–**6** and control experiments.

Considering the differences and similarities in the outcome of the various reaction conditions, a common mechanism with a central divergence point can be proposed. First, the diacetoxyiodobenzene reagent **7** would undergo ligand exchange with the halide to give mixed hypervalent iodine(III) **8** (Scheme 7a) [12]. We ruled out a possible direct reaction between the olefin and the hypervalent iodine(III) reagent as in the absence of any halide no reaction occurred within 1 h (Scheme 7b). A reductive elimination would then generate hypohalite R’OX which is presumably the active electrophilic species. If the reductive elimination only occurred after a second ligand exchange it would then give X_2_ which is generally a less efficient electrophilic species than the corresponding acetoxyhypohalite, especially trifluoroacetoxyhypohalites [26]. Indeed, when we reacted geranyl acetate **1a** with bromine we did isolate dibromo adduct **2a** but only with 47% yield and we also observed the formation of various byproducts such as ene-bromination adduct **6a’** (Scheme 7b) [27]. Moreover, for the iodination of enamides using the PIFA/KI combination we had already shown that the observed reactivity is more akin to the one induced by acetoxyhypoiodite [13]. Alternatively, the involvement of an ammonium-complexed halogen(I) species of type *n*-Bu_4_N[X(O_2_CCF_3_)_2_], as described by Kirschning [28] and later Muniz [29] cannot be ruled out although, to the best of our knowledge, it has yet to be characterized for X = Cl. Regardless of the actual halogenation species involved, regioselective halogenation of the terminal double bond of **1** would then give bridged halonium **9**. From there three manifolds can be at play. Pathway **a** involves the addition of an oxygenated nucleophile. For X = Br, this is the case when the bromide is the default reagent because of its slow addition (giving **3**) or because the nucleophile is the co-solvent (water giving **4** and to a lesser extent EtOH yielding **4’**). While the formation of **4** is related to the well-established NBS-mediated formation of bromohydrins in aqueous media [30], the formation of **3** is not so straightforward. For instance, submitting **1a** to NBS in the presence of trifluoroacetic acid led to a complex mixture of products, the major one being allylic bromide **6a’**. For X = I, the steric bulk would preclude the formation of the vicinal diiodide and only pathway **a** would thus be operative, yielding **5**. This is not the case for the dibromide **2**, which is readily formed through pathway **b** when the reaction takes place in the presence of excess bromide. Finally, if X = Cl, rapid deprotonation by the acetate (or the chloride) alpha to the chloronium bridge (pathway **c**) explains the formation of allylic chloride **6**.

## Conclusion

Overall, we have further extended the scope of the iodine(III)-mediated oxidative halogenation of terpenoids which now includes dibromination, bromo(trifluoro)acetoxylation, bromohydroxylation, iodo(trifluoro)acetoxylation and allylic ene-chlorination. The conditions are mild and selective and the reactions proceed rapidly with generally good yields. Application of this tunable strategy towards the rapid constitution of chemical libraries of biologically active molecules is now actively pursued in our laboratory.

## Experimental

### General procedure A: dibromination

To a solution of the geranyl derivative (1.0 equiv) in acetonitrile (0.05 M) at 0 °C, lithium bromide (2.0–2.4 equiv), 4 Å molecular sieves (1.0 mass equiv) and DIB (1.2–1.4 equiv) were added. After stirring for 5 min, the reaction mixture was diluted with EtOAc, filtered over alumina (EtOAc) and concentrated under reduced pressure before purification by flash chromatography.

### General procedure B: bromo(trifluoro)acetoxylation

To a solution of the geranyl derivative (1.0 equiv) in acetonitrile (0.07 M) cooled to 0 °C, PIFA (1.1 equiv) was added. A solution of tetra-*n*-butylammonium bromide (1.1 equiv) in acetonitrile (0.07 M) was then added dropwise over 5 min. After stirring for 10 min, the reaction mixture was diluted with EtOAc, saturated aqueous Na_2_S_2_O_3_ solution was added and the layers were separated. The aqueous layer was extracted twice with EtOAc. The combined organic extracts were washed with brine, dried over MgSO_4_, filtered and concentrated under reduced pressure before purification by flash chromatography.

### General procedure C: bromohydroxylation

To a solution of the geranyl derivative (1.0 equiv) in acetonitrile (0.1 M) cooled to −10 °C, DIB (1.4 equiv) was added. A solution of lithium bromide (1.6 equiv) in H_2_O (0.1 M) was then added dropwise over 5 min. After stirring at −10 °C for 10 min, the reaction mixture was diluted with EtOAc, filtered over alumina (EtOAc) and concentrated under reduced pressure before purification by flash chromatography.

### General procedure D: iodo(trifluoro)acetoxylation

To a solution of the geranyl derivative (1.0 equiv) in acetonitrile (0.1 M) at room temperature, 3 Å molecular sieves (1.0 mass equiv) and potassium iodide (2.4 equiv) were added. A solution of PIFA (1.5 equiv) in acetonitrile (0.1 M) was then added dropwise over 10 min and the reaction mixture was stirred for 15 min. The reaction mixture was diluted with EtOAc, Na_2_S_2_O_3_ solution (10%) was added and the layers were separated. The aqueous layer was extracted three times with EtOAc. The combined organic extracts were washed with water and brine, dried over MgSO_4_, filtered and concentrated under reduced pressure before purification by flash chromatography.

### General procedure E: allylic chlorination

To a solution of the geranyl derivative (1.0 equiv) in dichloromethane (stabilized with amylene, 0.1 M) cooled to 0 °C, PIFA (1.2 equiv) was added. A solution of tetra-*n*-butylammonium chloride (1.5 equiv) in DCM (0.1 M) was then added dropwise over 10 min and the reaction mixture was stirred for 10 min at 0 °C. The reaction mixture was diluted with EtOAc, Na_2_S_2_O_3_ solution (10%) was added and the layers were separated. The aqueous layer was extracted three times with EtOAc. The combined organic extracts were washed with water and brine, dried over MgSO_4_, filtered and concentrated under reduced pressure before purification by flash chromatography.

## Supporting Information

File 1 Full characterization data of all new compounds and copies of ^1^H and ^13^C NMR spectra.
