# Supplementary material for: Iodine(III)-mediated halogenations of acyclic monoterpenoids
Source: Beilstein J Org Chem. 2018 May 18;14:1103–11. doi: 10.3762/bjoc.14.96 (PMC6009204; doi:10.3762/bjoc.14.96)
Supplement: File 1 — Full characterization data of all new compounds and copies of 1H and 13C NMR spectra. [file Beilstein_J_Org_Chem-14-1103-s001.pdf]

Supporting Information

for

Iodine(III)-mediated halogenations of acyclic monoterpenoids

Laure Peilleron, Tatyana D. Grayfer, Joëlle Dubois, Robert H. Dodd and Kevin Cariou*

Address: Institut de Chimie des Substances Naturelles, CNRS UPR 2301, Université Paris-Sud, Université Paris-Saclay, Avenue de la Terrasse, 91198 Gif-sur-Yvette, France

Email: Kevin Cariou - kevin.cariou@cnrs.fr

* Corresponding author

Full characterization data of all new compounds and copies of ^{1}H and ^{13}C NMR spectra

1. General remarks **S2**
2. Procedures and analytical data for starting materials **S2**
3. Procedures and analytical data for dibromination products **S3**
4. Procedures and analytical data for trifluoroacetoxy-bromination products **S6**
5. Procedures and analytical data for hydroxy-bromination products **S9**
6. Procedures and analytical data for trifluoroacetoxy-iodination products **S12**
7. Procedures and analytical data for allylic-chlorination products **S15**
8. Copies of ^{1}H and ^{13}C NMR spectra for starting materials **S18**
9. Copies of ^{1}H and ^{13}C NMR spectra for halogenated products **S20**

1. General remarks

Infrared spectra were recorded on a Perkin Elmer Spectrum BX FT-IR spectrometer. Proton (^{1}H) and carbon (^{13}C) NMR spectra were recorded on Bruker Avance 300 MHz spectrometer (QNP or Dual probe). Carbon NMR (^{13}C) spectra were recorded at 75 MHz, using a broadband decoupled mode with the multiplicities obtained using a DEPT sequence. NMR experiments were carried out in deuterochloroform ($CDCl_3$), chemical shifts (δ) are reported in parts per million (ppm) with reference to $CDCl_3$ (^{1}H: 7.26; ^{13}C: 77.23) and deuterobenzene (C_6D_6), chemical shifts (δ) are reported in parts per million (ppm) with reference to C_6D_6 (^{1}H: 7.15; ^{13}C: 128.62). The following abbreviations are used for the proton spectra multiplicities: s: singlet, bs: broad singlet, d: doublet, t: triplet, q: quartet, m: multiplet, br: broad. Coupling constants (*J*) are reported in Hertz (Hz). High resolution mass spectra (HRMS) were obtained from a Time-of-Flight analyzer (ESI-MS). Thin-layer chromatography was performed on silica gel 60 F254 on aluminum plates (Merck) and visualized under a UVP Mineralight UVLS-28 lamp (254 nm) and with TLC stains (phosphomolybdic acid or potassium permanganate in ethanol). Flash chromatography was conducted on Merck silica gel 60 (40–63 µm) on a CombiFlash apparatus (Serlabo Technologies) at medium pressure (300 mbar), using standard settings.

All reagents were obtained from commercial suppliers unless stated otherwise. Where necessary, organic solvents were routinely dried and/or distilled prior to use and stored over molecular sieves under nitrogen. All reactions were conducted under an argon atmosphere, unless stated otherwise.

2. Procedures and analytical data for starting materials

(E)-N-(3,7-Dimethylocta-2,6-dien-1-yl)-4-methylbenzenesulfonamide (1d)

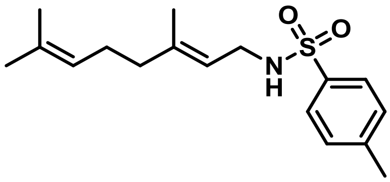

$C_{17}H_{25}NO_2S$, 307.4520 g/mol

To a solution of geranylamine (204.8 mg, 1.34 mmol) in anhydrous dichloromethane (8 mL) at room temperature, triethylamine (0.56 mL, 4.01 mmol) and DMAP (8.2 mg, 0.067 mmol) were added. The reaction mixture was cooled to 0 °C and *p*-toluenesulfonyl chloride (280.2 mg, 1.47 mmol) was added. After stirring at room temperature for 5 hours, the reaction mixture was quenched with saturated aqueous NH_4Cl solution. The aqueous layer was extracted twice with EtOAc. The combined organic extracts were washed with saturated aqueous NH_4Cl solution, water and brine, dried over $MgSO_4$, filtered and concentrated under reduced pressure. The crude residue

was purified by flash chromatography on silica gel (PE/EtOAc 100/0 → 95/5) to afford **1d** (384 mg, 93%) as a colorless oil. The chemical shifts of the product are in accordance with literature.[1]

NMR ^{1}H (300 MHz, $CDCl_3$): δ = 7.75 (d, *J* = 8.2 Hz, 2H, CH_{Ar}), 7.30 (d, *J* = 8.2 Hz, 2H, CH_{Ar}), 5.09–5.00 (m, 2H, =CH), 4.34 (bt, *J* = 5.4 Hz, 1H, NH), 3.55 (d, *J* = 6.4 Hz, 2H, CH_2-NHTs), 2.44 (s, 3H, CH_3-Ar), 2.00–1.90 (m, 4H, CH_2), 1.67 (s, 3H, $(CH_3)_2C$=C), 1.57 (s, 3H, $(CH_3)_2C$=C), 1.54 (s, 3H, CH_3-C=C).

NMR ^{13}C (75 MHz, $CDCl_3$): δ = 143.3 (C_q), 141.1 (C_q), 137.1 (C_q), 131.8 (C_q), 129.6 (2CH), 127.2 (2CH), 123.6 (CH), 118.6 (CH), 41.0 ($NHCH_2$), 39.3 (CH_2), 26.2 (CH_2), 25.6 (CH_3), 21.5 (CH_3), 17.7 (CH_3), 16.2 (CH_3).

HRMS (ESI): *m/z* calcd for $C_{17}H_{26}NO_2S^+$, $[M+H]^+$ 308.1679, found 308.1689.
m/z calcd for $C_{17}H_{25}NNaO_2S^+$, $[M+Na]^+$ 330.1498, found 330.1507.
m/z calcd for $C_{19}H_{28}N_2NaO_2S^+$, $[M+CH_3CN+Na]^+$ 371.1764, found 371.1768.

IR (cm^{-1}): 3278, 2919, 1424, 1324, 1157, 1094, 1048, 814, 662.

3. Procedures and analytical data for dibromination products

***(E)-6,7-Dibromo-3,7-dimethyloct-2-en-1-yl acetate* (2a)**

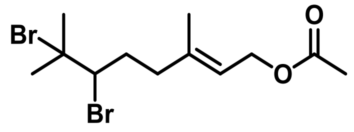

$C_{12}H_{20}Br_2O_2$, 356.0980 g/mol

General procedure A was applied to geranyl acetate (**1a**, 28 mg, 0.14 mmol). The crude residue was purified by flash chromatography on silica gel (PE/EtOAc 100/0 → 95/5) to afford **2a** (48 mg, 91%) as a colorless oil. The chemical shifts of the product are in accordance with literature.[2]

NMR ^{1}H (300 MHz, CDCl3): δ = 5.43 (bt, *J* = 7.0 Hz, =CH), 4.59 (d, *J* = 7.0 Hz, 2H, CH_2-OAc), 4.11 (dd, *J* = 10.0, 1.2 Hz, 1H, CH-Br), 2.62–2.52 (m, 1H, CH_2), 2.46–2.35 (m, 1H, CH_2), 2.25–2.15 (m, 1H, CH_2), 2.05 (s, 3H, Ac), 1.97 (s, 3H, CH_3), 1.95–1.82 (m, 1H, CH_2), 1.80 (s, 3H, CH_3), 1.73 (s, 3H, CH_3-C=C).

NMR ^{13}C (75 MHz, CDCl3): δ = 171.2 (C=O), 140.3 (C_q), 120.2 (CH), 68.8 (C_q), 65.8 (CH-Br), 61.4 (OCH_2), 37.9 (CH_2), 35.6 (CH_3), 33.7 (CH_2), 28.3 (CH_3), 21.2 (CH_3), 16.6 (CH_3).

HRMS (ESI): m/z calcd for $C_{10}H_{16}{}^{79}Br^+$, $[M–Br–HOAc]^+$ 215.0430, found 215.0438.

IR (cm^{-1}): 2978, 2929, 1737, 1454, 1386, 1370, 1227, 1097, 1021, 1022, 955, 869, 820, 777.

[1] F. Inagaki; S. Hira; C. Mukai; *Synlett*, **2017**, *28*, 2143–2146.
[2] A. M. Moiseenkov, V. A. Dragan, A. V. Lozanova, V. V. Veselovskii. *Russ. Chem. Bull.* **1988**, *8*, 1797–1803.

(Z)-6,7-Dibromo-3,7-dimethyloct-2-en-1-yl acetate (2b)

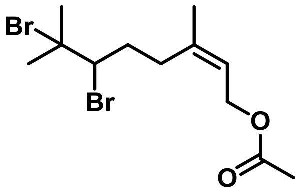

$C_{12}H_{20}Br_2O_2$, 356.0980 g/mol

General procedure A was applied to neryl acetate (**1b**, 50 mg, 0.25 mmol). The crude residue was purified by flash chromatography on silica gel (PE/EtOAc 98/2 → 95/5) to afford **2b** (86 mg, 97%) as a colorless oil.

NMR ^{1}H (300 MHz, $CDCl_3$): δ = 5.38 (bt, *J* = 7.3 Hz, 1H, =CH), 4.55 (dd, *J* = 7.3, 2.3 Hz, 2H, CH_2-OAc), 4.03 (dd, *J* = 11.0, 1.3 Hz, 1H, CH-Br), 2.57–2.46 (m, 1H, CH_2), 2.42–2.22 (m, 2H, CH_2), 1.99 (s, 3H, Ac), 1.91 (s, 3H, CH_3), 1.89–1.76 (m, 1H, CH_2), 1.73 (s, 6H, CH_3-C=C and CH_3).

NMR ^{13}C (75 MHz, $CDCl_3$): δ = 171.1 (C=O), 140.7 (C_q), 121.1 (CH), 68.5 (C_q), 65.5 (CH-Br), 61.0 (OCH_2), 35.4 (CH_3), 33.9 (CH_2), 30.2 (CH_2), 28.0 (CH_3), 23.3 (CH_3), 21.1 (CH_3).

HRMS (ESI): *m/z* calcd for $C_{10}H_{16}{}^{79}Br^+$, $[M–Br–HOAc]^+$ 215.0430, found 215.0430.

IR (cm^{-1}): 2975, 2933, 1737, 1456, 1442, 1371, 1226, 1097, 1021, 954.

***(E)-6,7-Dibromo-3,7-dimethyloct-2-en-1-ol* (2c)**

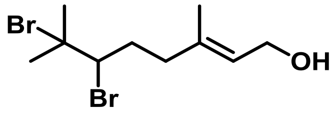

$C_{10}H_{18}Br_2O$, 314.0610 g/mol

General procedure A was applied to geraniol (**1c**, 53 mg, 0.34 mmol). The crude product was purified by flash chromatography on silica gel (DCM 100%) to afford **2c** (67 mg, 63%) as a colorless oil.

NMR ^{1}H (300 MHz, CDCl3): δ = 5.52 (bt, *J* = 6.8 Hz, 1H, =CH), 4.18 (bd, *J* = 6.8 Hz, 2H, CH_2-OH), 4.15 (dd, *J* = 11.1, 1.3 Hz, 1H, CH-Br), 2.64–2.53 (m, 1H, CH_2), 2.45–2.36 (m, 1H, CH_2), 2.25–2.15 (m, 1H, CH_2), 1.98 (s, 3H, CH_3), 1.96–1.81 (m, 1H, CH_2), 1.81 (s, 3H, CH_3), 1.71 (s, 3H, CH_3-C=C).

NMR ^{13}C (75 MHz, CDCl3): δ = 137.7 (C_q), 125.2 (CH), 68.9 (C_q), 65.8 (CH-Br), 59.5 (OCH_2), 37.8 (CH_2), 35.6 (CH_3), 33.7 (CH_2), 28.3 (CH_3), 16.4 (CH_3).

HRMS (ESI): m/z calcd for $C_{10}H_{16}{}^{79}Br^+$, $[M-Br-H_2O]^+$ 215.0430, found 215.0432.

IR (cm^{-1}): 3322, 2976, 2928, 2860, 1440, 1386, 1370, 1227, 1096, 997.

(E)-N-(6,7-Dibromo-3,7-dimethyloct-2-en-1-yl)-4-methylbenzenesulfonamide (2d)

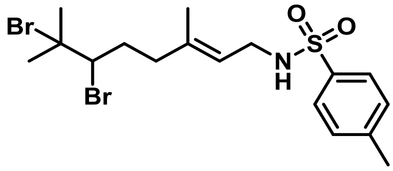

$C_{17}H_{25}Br_2NO_2S$, 467.2600 g/mol

General procedure A was applied to *N*-tosyl geranylamine (**1d**, 38 mg, 0.124 mmol). The crude residue was purified by flash chromatography on silica gel (PE/EtOAc 90/10) to afford **2d** (28.6 mg, 50%) as a colorless oil.

NMR ^{1}H (300 MHz, CDCl$_3$): δ = 7.76 (d, *J* = 8.1 Hz, 2H, CH_{Ar}), 7.31 (d, *J* = 8.1 Hz, 2H, CH_{Ar}), 5.15 (bt, *J* = 6.7 Hz, 1H, =CH), 4.42 (bs, 1H, NH), 4.05 (d, *J* = 10.9 Hz, 1H, CH-Br), 3.58 (bt, *J* = 6.5 Hz, 2H, CH_2-NHTs), 2.54–2.48 (m, 1H, CH_2), 2.44 (s, 3H, CH_3-Ar), 2.34–2.25 (m, 1H, CH_2), 2.15–2.05 (m, 1H, CH_2), 1.97 (s, 3H, CH_3), 1.84–1.74 (m, 1H, CH_2), 1.79 (s, 3H, CH_3), 1.59 (s, 3H, CH_3-C=C).

NMR ^{13}C (75 MHz, CDCl$_3$): δ = 143.4 (C_q), 139.0 (C_q), 137.1 (C_q), 129.7 (2CH), 127.2 (2CH), 120.4 (CH), 68.7 (C_q), 65.5 (CH-Br), 40.9 ($NHCH_2$), 37.5 (CH_2), 35.4 (CH_3), 33.5 (CH_2), 28.1 (CH_3), 21.6 (CH_3), 16.2 (CH_3).

HRMS (ESI): *m/z* calcd for $C_{17}H_{25}{}^{79}Br^{81}BrNNaO_2S^+$, $[M+Na]^+$ 489.9844, found 489.9841.
m/z calcd for $C_{19}H_{28}{}^{79}Br^{81}BrN_2NaO_2S^+$, $[M+CH_3CN+Na]^+$ 531.0109, found 531.0106.

IR (cm^{-1}): 3279, 2926, 1429, 1324, 1157, 1095, 1044, 909, 814, 731, 662.

6,7-Dibromo-7-methyl-3-methyleneoct-1-ene (2e)

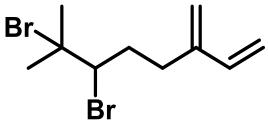

$C_{10}H_{16}Br_2$, 296.0460 g/mol

General procedure A was applied to myrcene (**1e**, 32.3 mg, 0.237 mmol) to afford **2e** (55 mg, 78%) as a colorless oil. The chemical shifts of the product are in accordance with literature .[3] *Caution is to be taken since this product is volatile.*

NMR ^{1}H (300 MHz, CDCl$_3$): δ = 6.35 (dd, *J* = 17.6, 10.6 Hz, 1H, C**H**=CH$_2$), 5.29 (d, *J* = 17.6 Hz, 1H, C**H**$_2$=CH), 5.14 (d, *J* = 10.6 Hz, 1H, C**H**$_2$=CH), 5.11 (s, 2H, C**H**$_2$=C), 4.21 (bd, *J* = 11.0 Hz, 1H, CH-Br), 2.74–2.58 (m, 2H, CH_2), 2.41–2.30 (m, 1H, CH_2), 2.07–1.93 (m, 1H, CH_2), 1.99 (s, 3H, CH_3), 1.83 (s, 3H, CH_3).

NMR ^{13}C (75 MHz, CDCl$_3$): δ = 144.7 (C_q), 138.4 (CH), 116.9 (CH_2), 113.8 (CH_2), 68.4 (C_q), 66.4 (CH-Br), 35.3 (CH_3), 34.6 (CH_2), 30.1 (CH_2), 28.3 (CH_3).

HRMS (ESI): no ionization could be observed for this compound.

IR (cm^{-1}): 2977, 2931, 1595, 1456, 1387, 1371, 1221, 1097, 990, 897.

[3] T. Kato, I. Ichinose, *J. Chem. Soc., Perkin Trans. 1*, **1980**, *0*, 1051-1056.

4. Procedures and analytical data for trifluoroacetoxy-bromination products

***(E)*-8-Acetoxy-3-bromo-2,6-dimethyloct-6-en-2-yl 2,2,2-trifluoroacetate** (**3a**)

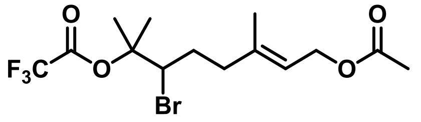

$C_{14}H_{20}BrF_3O_4$, 389.2092 g/mol

General procedure B was applied to geranyl acetate (**1a**, 39 mg, 0.20 mmol). The crude residue was purified by flash column chromatography (PE/EtOAc 100/0 ⟶ 95/5) to afford **3a** (60 mg, 77%) as a colorless oil.

NMR ^{1}H (300 MHz, CDCl3): δ = 5.42 (tq, *J* = 7.0, 1.1 Hz, 1H, =CH), 4.59 (d, *J* = 7.0 Hz, 2H, CH_2-OAc), 4.36 (dd, *J* = 11.1, 2.1 Hz, 1H, CH-Br), 2.45–2.36 (m, 1H, CH_2), 2.23–2.13 (m, 1H, CH_2), 2.05 (s, 3H, Ac), 2.04–1.92 (m, 1H, CH_2), 1.87–1.77 (m, 1H, CH_2), 1.70 (s, 9H, CH_3).

NMR ^{13}C (75 MHz, CDCl3): δ = 171.3 (C=O), 156.0 (q, *J* = 42 Hz, CF_3**C**=O), 140.0 (C_q), 120.5 (CH), 114.4 (q, *J* = 287 Hz, CF_3), 89.5 (C_q), 61.3 (OCH_2), 59.4 (CH-Br), 37.6 (CH_2), 31.2 (CH_2), 24.3 (CH_3), 22.5 (CH_3), 21.2 (CH_3), 16.5 (CH_3).

NMR ^{19}F (300 Hz, CDCl3): δ = -75.6.

HRMS (APPI): m/z calcd for $C_{14}H_{20}F_3O_4^+$, $[M–Br]^+$ 309.1308, found 309.1319.

IR (cm-1): 2993, 2950, 1780, 1737, 1446, 1367, 1218, 1159, 1116, 1023, 957, 871, 852, 774, 730.

***(Z)*-8-Acetoxy-3-bromo-2,6-dimethyloct-6-en-2-yl 2,2,2-trifluoroacetate (3b)**

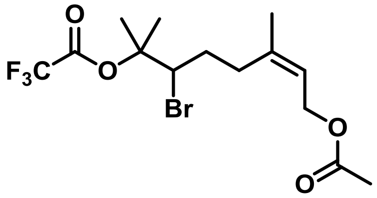

$C_{14}H_{20}BrF_3O_4$, 389.2092 g/mol

General procedure B was applied to neryl acetate (**3b**, 57 mg, 0.290 mmol). The crude residue was purified by flash column chromatography (PE/EtOAc 100/0 ⟶ 95/05) to afford **3b** (85 mg, 75%) as a colorless oil.

NMR ^{1}H (300 MHz, CDCl3): δ = 5.45 (bt, *J* = 7.3 Hz, 1H, =CH), 4.61 (d, *J* = 7.3 Hz, 2H, CH_2-OAc), 4.32 (dd, *J* = 11.3, 1.7 Hz, 1H, CH-Br), 2.46–2.30 (m, 2H, CH_2), 2.06 (s, 3H, Ac), 2.04–1.96 (m, 1H, CH_2), 1.88–1.80 (m, 1H, CH_2), 1.76 (s, 3H, CH_3-C=C), 1.71 (s, 6H, CH_3).

NMR ^{13}C (75 MHz, CDCl3): δ = 170.9 (C=O), 155.8 (q, *J* = 41.7 Hz, CF_3**C**=O), 140.2 (C_q), 121.4 (CH), 114.2 (q, *J* = 287 Hz, CF_3), 89.2 (C_q), 60.8 (OCH_2), 59.3 (CH-Br), 31.3 (CH_2), 30.1 (CH_2), 24.0 (CH_3), 23.0 (CH_3), 22.2 (CH_3), 21.0 (CH_3).

NMR ^{19}F (300 Hz, CDCl3): δ = -75.6.

HRMS (ESI): m/z calcd for $C_{14}H_{20}{}^{79}BrF_3NaO_4^+$, $[M+Na]^+$ 411.0389, found 411.0398.
m/z calcd for $C_{16}H_{23}{}^{79}BrF_3NNaO_4^+$, $[M+CH_3CN+Na]^+$ 452.0655, found 452.0661
m/z calcd for $C_{14}H_{20}{}^{81}BrF_3NaO_4^+$, $[M+Na]^+$ 413.0369, found 413.0388.
m/z calcd for $C_{16}H_{23}{}^{81}BrF_3NNaO_4^+$, $[M+CH_3CN+Na]^+$ 454.0635, found 454.0649

IR (cm^{-1}): 2938, 1780, 1737, 1446, 1367, 1219, 1159, 1116, 1022, 956, 870, 850, 774, 730.

(E)-3-Bromo-8-hydroxy-2,6-dimethyloct-6-en-2-yl 2,2,2-trifluoroacetate **(3c)**

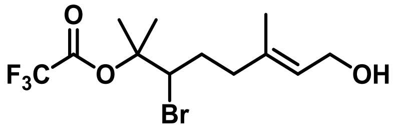

$C_{12}H_{18}BrF_3O_3$, 347.1722 g/mol

General procedure B was applied to geraniol (**1c**, 46 mg, 0.30 mmol). The crude residue was purified by flash column chromatography (PE/EtOAc 100/0 ⟶ 80/20) to afford **3c** (59 mg, 57%) as a colorless oil.

NMR ^{1}H (300 MHz, CDCl3): δ = 5.48 (bt, *J* = 6.8 Hz, =CH), 4.40 (dd, *J* = 11.3, 1.9 Hz, 1H, CH-Br), 4.16 (d, *J* = 6.8 Hz, 2H, CH_2-OH), 2.43–2.33 (m, 1H, CH_2), 2.22–2.11 (m, 1H, CH_2), 2.03–1.92 (m, 1H, CH_2), 1.88–1.76 (m, 1H, CH_2), 1.70 (s, 3H, CH_3), 1.69 (s, 3H, CH_3), 1.66 (s, 3H, CH_3-C=C).

NMR ^{13}C (75 MHz, CDCl3): δ = 156.1 (q, *J* = 42 Hz, CF_3**C**=O), 137.4 (C_q), 125.6 (CH), 114.4 (q, *J* = 287 Hz, CF_3), 89.6 (C_q), 59.4 (OCH_2), 59.2 (CH-Br), 37.5 (CH_2), 31.2 (CH_2), 24.3 (CH_3), 22.5 (CH_3), 16.2 (CH_3),

NMR ^{19}F (300 Hz, CDCl3): δ = -75.6.

HRMS (ESI): m/z calcd for $C_{12}H_{18}{}^{79}BrF_3O_3{}^{35}Cl^-$, $[M+Cl]^-$ 381.0085, found 381.0076.

IR (cm^{-1}): 3380-3340, 2991, 2927, 2857, 1781, 1670, 1463, 1446, 1370, 1222, 1165, 1117, 1004, 870, 848, 775, 730, 635, 526.

(E)-3-Bromo-2,6-dimethyl-8-((4-methylphenyl)sulfonamido)oct-6-en-2-yl 2,2,2-trifluoroacetate (3d)

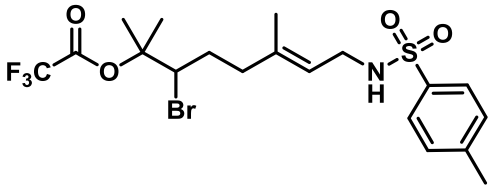

$C_{19}H_{25}BrF_3NO_4S$, 500.3712 g/mol

General procedure B was applied to *N*-tosyl geranylamine (**1d**, 36 mg, 0.117 mmol). The crude residue was purified by flash chromatography on silica gel (PE/EtOAc 85/15) to afford **3d** (49 mg, 84%) as a colorless oil.

NMR ^{1}H (300 MHz, CDCl$_3$): δ = 7.78 (d, *J* = 8.2 Hz, 2H, CH_{Ar}), 7.33 (d, *J* = 8.2 Hz, 2H, CH_{Ar}), 5.16 (tq, *J* = 7.0, 0.9 Hz, 1H, =CH), 4.50 (bs, 1H, NH), 4.35 (dd, *J* = 11.1, 1.9 Hz, 1H, CH-Br), 3.59 (t, *J* = 7.0 Hz, 2H, CH_2-NHTs), 2.46 (s, 3H, CH_3-Ar), 2.34–2.25 (m, 1H, CH_2), 2.15–2.05 (m, 1H, CH_2), 1.96–1.85 (m, 1H, CH_2), 1.81–1.79 (m, 1H, CH_2), 1.72 (s, 3H, CH_3), 1.70 (s, 3H, CH_3), 1.56 (s, 3H, CH_3-C=C).

NMR ^{13}C (75 MHz, CDCl$_3$): δ = 155.9 (q, *J* = 42.8 Hz, CF_3**C**=O), 143.5 (C_q), 138.7 (C_q), 137.0 (C_q), 129.7 (2CH), 127.2 (2CH), 120.8 (CH), 114.2 (q, *J* = 287 Hz, CF_3), 89.5 (C_q), 58.7 (CH-Br), 40.9 ($NHCH_2$), 37.1 (CH_2), 30.8 (CH_2), 24.1 (CH_3), 22.4 (CH_3), 21.6 (CH_3), 16.0 (CH_3).

NMR ^{19}F (300 Hz, CDCl3): δ = -75.5.

HRMS (ESI): m/z calcd for $C_{19}H_{25}{}^{79}BrF_3NNaO_4S^+$, $[M+Na]^+$ 522.0532, found 522.0536
m/z calcd for $C_{21}H_{28}{}^{79}BrF_3N_2NaO_4S^+$, $[M+CH_3CN+Na]^+$ 563.0797, found 563.0797
m/z calcd for $C_{19}H_{25}{}^{81}BrF_3NNaO_4S^+$, $[M+Na]^+$ 524.0511, found 524.0516
m/z calcd for $C_{21}H_{28}{}^{81}BrF_3N_2NaO_4S^+$, $[M+CH_3CN+Na]^+$ 565.0777, found 565.0784

IR (cm^{-1}): 3280, 2928, 1779, 1599, 1370, 1326, 1218, 1155, 814, 663.

3-Bromo-2-methyl-6-methyleneoct-7-en-2-yl 2,2,2-trifluoroacetate (3e)

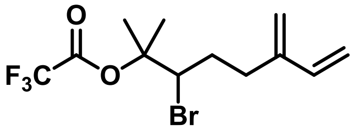

$C_{12}H_{16}BrF_3O_2$, 329.1572 g/mol

General procedure B was applied to myrcene (**1e**, 44.8 mg, 0.329 mmol). The crude residue was purified by flash chromatography on silica gel (PE 100%) to afford **3e** (72 mg, 67%) as a colorless oil. *Caution is to be taken since this product is volatile.*

NMR ^{1}H (300 MHz, CDCl$_3$): δ = 6.24 (dd, *J* = 17.7, 10.8 Hz, 1H, C**H**=CH_2), 5.14 (d, *J* = 17.7 Hz, 1H, C**H**$_2$=CH), 5.04 (d, *J* = 10.8 Hz, 1H, C**H**$_2$=CH), 5.02 (s, 1H, C**H**$_2$=C), 5.00 (s, 1H, C**H**$_2$=C), 4.35 (dd, *J* = 11.2, 1.8 Hz, 1H, CH-Br), 2.64–2.55 (m, 1H, CH_2), 2.31–2.21 (m, 1H, CH_2), 2.02–1.91 (m, 1H, CH_2), 1.87–1.74 (m, 1H, CH_2), 1.62 (s, 3H, CH_3), 1.61 (s, 3H, CH_3).

NMR ^{13}C (75 MHz, CDCl$_3$): δ = 155.8 (q, *J* = 41.7 Hz, CF_3**C**=O), 144.4 (C_q), 138.1 (CH), 117.3 (CH_2), 113.8 (CH_2), 114.2 (q, *J* = 287 Hz, CF_3), 89.2 (C_q), 59.5 (CH-Br), 31.8 (CH_2), 29.8 (CH_2), 24.0 (CH_3), 22.4 (CH_3).

NMR ^{19}F (300 Hz, CDCl3): δ = -75.6.

HRMS (ESI): no ionization could be observed for this compound.

IR (cm^{-1}): 2948, 1779, 1596, 1461, 1367, 1218, 1159, 1116, 992, 901, 872, 854, 774, 731.

5. Procedures and analytical data for hydroxy-bromination products

(E)-6-Bromo-7-hydroxy-3,7-dimethyloct-2-en-1-yl acetate **(4a)**

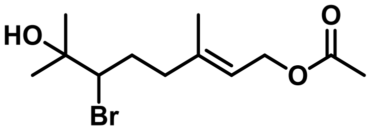

$C_{12}H_{21}BrO_3$, 293.2010 g/mol

General procedure C was applied to geranyl acetate (**1a**, 55.8 mg, 0.284 mmol). The crude product was purified by flash chromatography on silica gel (PE/EtOAc 100/0 ⟶ 90/10 ⟶ 80/20) to afford **4a** (58 mg, 70%) as a colorless oil. The chemical shifts of the product are in accordance with literature.[4]

NMR ^{1}H (300 MHz, $CDCl_3$): δ = 5.31 (tq, *J* = 7.0, 1.1 Hz, 1H, =CH), 4.51 (d, *J* = 7.0 Hz, 2H, CH_2-OAc), 3.86 (dd, *J* = 11.3, 2.1 Hz, 1H, CH-Br), 2.39–2.30 (m, 1H, CH_2), 2.14–2.04 (m, 1H, CH_2), 2.02–1.91 (m, 1H, CH_2), 1.99 (s, 3H, Ac), 1.83–1.70 (m, 1H, CH_2), 1.64 (s, 3H, CH_3-C=C), 1.28 (s, 3H, CH_3), 1.27 (s, 3H, CH_3).

NMR ^{13}C (75 MHz, $CDCl_3$): δ = 171.1 (C=O), 140.4 (C_q), 119.7 (CH), 72.5 (C_q), 70.2 (CH-Br), 61.2 (OCH_2), 38.1 (CH_2), 31.8 (CH_2), 26.6 (CH_3), 26.0 (CH_3), 21.0 (CH_3), 16.4 (CH_3).

HRMS (ESI): m/z calcd for $C_{12}H_{21}{}^{79}BrNaO_3^+$, $[M+Na]^+$ 315.0566, found 315.0575.
m/z calcd for $C_{14}H_{24}{}^{79}BrNNaO_3^+$, $[M+CH_3CN+Na]^+$ 356.0832, found 356.0838
m/z calcd for $C_{12}H_{21}{}^{81}BrNaO_3^+$, $[M+Na]^+$ 317.0546, found 317.0560.
m/z calcd for $C_{14}H_{24}{}^{81}BrNNaO_3^+$, $[M+CH_3CN+Na]^+$ 358.0812, found 358.0826

IR (cm^{-1}): 3454, 2978, 2932, 1718, 1670, 1444, 1366, 1230, 1120, 1023, 955, 908, 774, 733.

(Z)-6-Bromo-7-hydroxy-3,7-dimethyloct-2-en-1-yl acetate (4b)

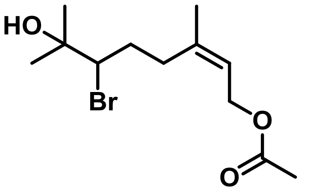

$C_{12}H_{21}BrO_3$, 293.2010 g/mol

General procedure C was applied to neryl acetate (**1b**, 58.2 mg, 0.297 mmol). The crude residue was purified by flash chromatography on silica gel (PE/EtOAc 100/0 ⟶ 90/10 ⟶ 80/20) to afford **4b** (52.6 mg, 61%) as a colorless oil.

[4] M. Nahmany, A. Melman, *Tetrahedron*, **2005**, *61*, 7481–7488.

NMR ^{1}H (300 MHz, $CDCl_3$): δ = 5.34 (bt, *J* = 7.3 Hz, 1H, =CH), 4.54 (bd, *J* = 7.3 Hz, 2H, CH_2-OAc), 3.85 (dd, *J* = 11.2, 2.0 Hz, 1H, CH-Br), 2.26 (t, *J* = 7.4 Hz, 2H, CH_2), 1.98 (s, 3H, Ac), 1.97–1.91 (m, 1H, CH_2), 1.82–1.72 (m, 1H, CH_2), 1.69 (s, 3H, CH_3-C=C), 1.28 (s, 3H, CH_3), 1.27 (s, 3H, CH_3).
NMR ^{13}C (75 MHz, $CDCl_3$): δ = 171.0 (C=O), 140.9 (C_q), 120.8 (CH), 72.4 (C_q), 70.1 (CH-Br), 61.0 (OCH_2), 32.2 (CH_2), 30.8 (CH_2), 26.6 (CH_3), 26.1 (CH_3), 23.3 (CH_3), 21.1 (CH_3).

HRMS (ESI): m/z calcd for $C_{12}H_{21}{}^{79}BrNaO_3^+$, $[M+Na]^+$ 315.0566, found 315.0583
m/z calcd for $C_{14}H_{24}{}^{79}BrNNaO_3^+$, $[M+CH_3CN+Na]^+$ 356.0832, found 356.0844
m/z calcd for $C_{12}H_{21}{}^{81}BrNaO_3^+$, $[M+Na]^+$ 317.0546, found 317.0565
m/z calcd for $C_{14}H_{24}{}^{81}BrNNaO_3^+$, $[M+CH_3CN+Na]^+$ 358.0812, found 358.0830

IR (cm^{-1}): 3453, 2974, 2936, 1718, 1668, 1445, 1378, 1231, 1120, 1023, 955, 911, 842, 774.

(E)-6-Bromo-3,7-dimethyloct-2-ene-1,7-diol **(4c)**

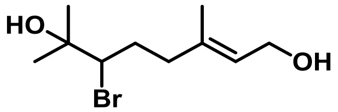

$C_{10}H_{19}BrO_2$, 251.1640 g/mol

General procedure C was applied to geraniol (**1c**, 67.7 mg, 0.439 mmol). The crude product was purified by flash chromatography on silica gel (PE/EtOAc 100/0 ⟶ 70/30) to afford **4c** (54.8 mg, 50%) as a colorless oil. The chemical shifts of the product are in accordance with literature.[5]

NMR ^{1}H (300 MHz, $CDCl_3$): δ = 5.46 (tq, *J* = 6.8, 1.1 Hz, 1H, =CH), 4.15 (d, *J* = 6.8 Hz, 2H, CH_2-OH), 3.95 (dd, *J* = 11.3, 1.9 Hz, 1H, CH-Br), 2.44–2.35 (m, 1H, CH_2), 2.21–2.10 (m, 1H, CH_2), 2.09–1.99 (m, 1H, CH_2), 1.89–1.78 (m, 1H, CH_2), 1.68 (s, 3H, CH_3-C=C), 1.35 (s, 3H, CH_3), 1.34 (s, 3H, CH_3).

NMR ^{13}C (75 MHz, $CDCl_3$): δ = 137.8 (C_q), 124.7 (CH), 72.5 (C_q), 70.1 (CH-Br), 59.2 (OCH_2), 38.0 (CH_2), 31.8 (CH_2), 26.5 (CH_3), 26.1 (CH_3), 16.2 (CH_3).

IR (cm^{-1}): 3384, 2975, 2933, 1728, 1652, 1456, 1379, 1219, 1129, 1063, 913, 776.

(E)-N-(6-Bromo-7-hydroxy-3,7-dimethyloct-2-en-1-yl)-4-methylbenzenesulfonamide (4d)

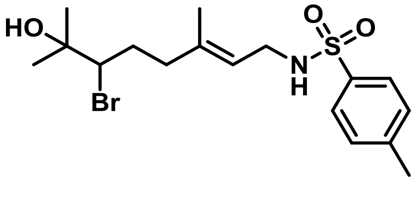

$C_{17}H_{26}BrNO_3S$, 404.3630 g/mol

General procedure C was applied to *N*-tosyl geranylamine (**1d**, 50.3 mg, 0.164 mmol). The crude residue was purified by flash chromatography on silica gel (PE/EtOAc 100/0 ⟶ 80/20 ⟶ 70/30) to afford **4d** (17.5 mg, 27%) as a colorless oil.

[5] T. Hoshino, A. Chiba, N. Abe, *Chem. Eur. J.* **2012**, *18*, 13108 – 13116.

NMR ^{1}H (300 MHz, CDCl$_3$): δ = 7.76 (d, *J* = 8.0 Hz, 2H, CH_{Ar}), 7.32 (d, *J* = 8.0 Hz, 2H, CH_{Ar}), 5.13 (bt, *J* = 7.0 Hz, 1H, =CH), 4.51 (bs, 1H, NH), 3.88 (d, *J* = 11.3 Hz, 1H, CH-Br), 3.56 (bt, *J* = 6.0 Hz, 2H, CH_2-NHTs), 2.45 (s, 3H, CH_3-Ar), 2.34–2.25 (m, 1H, CH_2), 2.11–2.01 (m, 1H, CH_2), 1.98–1.91 (m, 1H, CH_2), 1.80–1.66 (m, 1H, CH_2), 1.57 (s, 3H, CH_3-C=C), 1.34 (s, 6H, CH_3).

NMR ^{13}C (75 MHz, CDCl$_3$): δ = 143.4 (C_q), 139.2 (C_q), 137.0 (C_q), 129.7 (2CH), 127.2 (2CH), 120.1 (CH), 72.5 (C_q), 69.9 (CH-Br), 40.9 ($NHCH_2$), 37.9 (CH_2), 31.6 (CH_2), 26.4 (CH_3), 26.1 (CH_3), 21.6 (CH_3), 16.2 (CH_3).

HRMS (ESI): *m/z* calcd for $C_{17}H_{26}{}^{79}BrNNaO_3S^+$, $[M+Na]^+$ 426.0709, found 426.0711.
m/z calcd for $C_{19}H_{29}{}^{79}BrN_2NaO_3S^+$, $[M+CH_3CN+Na]^+$ 467.0974, found 467.0972.
m/z calcd for $C_{17}H_{26}{}^{81}BrNNaO_3S^+$, $[M+Na]^+$ 428.0688, found 428.0698.
m/z calcd for $C_{19}H_{29}{}^{81}BrN_2NaO_3S^+$, $[M+ CH_3CN+Na]^+$ 469.0954, found 469.0963.

IR (cm^{-1}): 3491, 3268, 2976, 2926, 1599, 1430, 1321, 1154, 1093, 1043, 907, 814, 731, 662.

3-Bromo-2-methyl-6-methyleneoct-7-en-2-ol (4e)

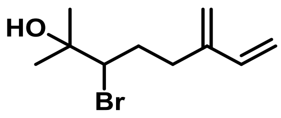

$C_{10}H_{17}BrO$, 233.1490 g/mol

General procedure C was applied to myrcene (**1e**, 50.6 mg, 0.371 mmol). The crude residue was purified by flash chromatography on silica gel (PE/EtOAc 100/0 ⟶ 95/5) to afford **4e** (18.5 mg, 21%) as a colorless oil. *Caution is to be taken since this product is volatile.*

NMR ^{1}H (300 MHz, CDCl$_3$): δ = 6.34 (dd, *J* = 17.7, 10.7 Hz, 1H, C**H**=CH_2), 5.26 (d, *J* = 17.7 Hz, 1H, C**H**$_2$=CH), 5.13 (d, *J* = 10.7 Hz, 1H, C**H**$_2$=CH), 5.09 (s, 2H, C**H**$_2$=C), 4.04 (dd, *J* = 11.2, 1.6 Hz, 1H, CH-Br), 2.73–2.64 (m, 1H, CH_2), 2.38–2.28 (m, 1H, CH_2), 2.14–2.03 (m, 1H, CH_2), 1.99–1.86 (m, 1H, CH_2), 1.36 (s, 3H, CH_3), 1.35 (s, 3H, CH_3).

NMR ^{13}C (75 MHz, CDCl$_3$): δ = 144.8 (C_q), 138.3 (CH), 116.9 (CH_2), 113.8 (CH_2), 72.5 (C_q), 71.0 (CH-Br), 32.7 (CH_2), 30.4 (CH_2), 26.7 (CH_3), 25.8 (CH_3).

HRMS (ESI): no ionization could be observed for this compound.

IR (cm^{-1}): 3430, 2977, 2931, 1595, 1460, 1367, 1221, 1157, 1115, 992, 897, 791.

6. Procedures and analytical data for trifluoroacetoxy-iodination products

***(E)-8-Acetoxy-3-iodo-2,6-dimethyloct-6-en-2-yl 2,2,2-trifluoroacetate* (5a)**

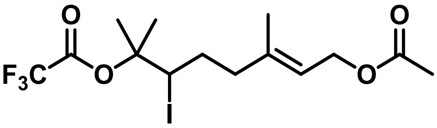

$C_{14}H_{20}F_3IO_4$, 436.2097 g/mol

General procedure D was applied to geranyl acetate (**1a**, 51.2 mg, 0.261 mmol). The crude residue was purified by flash column chromatography (PE/EtOAc 100/0 ⟶ 95/5) to afford **5a** (80 mg, 70%) as a colorless oil.

NMR ^{1}H (300 MHz, $CDCl_3$): δ = 5.43 (tq, *J* = 7.0, 1.2 Hz, 1H, =CH), 4.60 (d, *J* = 7.0 Hz, 2H, CH_2-OAc), 4.48 (dd, *J* = 10.5, 3.0 Hz, 1H, CH-I), 2.46–2.37 (m, 1H, CH_2), 2.21–2.11 (m, 1H, CH_2), 2.07 (s, 3H, Ac), 1.94 (d, *J* = 5.3 Hz, 1H, CH_2), 1.88–1.80 (m, 1H, CH_2), 1.77 (s, 3H, CH_3), 1.76 (s, 3H, CH_3), 1.71 (bs, 3H, CH_3-C=C).

NMR ^{13}C (75 MHz, $CDCl_3$): δ = 171.0 (C=O), 155.8 (q, *J* = 42.3 Hz, CF_3**C**=O), 139.5 (C_q), 120.4 (CH), 114.1 (q, *J* = 287 Hz, CF_3), 89.3 (C_q), 61.0 (OCH_2), 41.6 (CH-I), 39.1 (CH_2), 32.5 (CH_2), 25.7 (CH_3), 22.8 (CH_3), 21.0 (CH_3), 16.2 (CH_3).

NMR ^{19}F (300 Hz, $CDCl_3$): δ = -75.5.

HRMS (ESI): m/z calcd for $C_{14}H_{20}F_3INaO_4^+$, $[M+Na]^+$ 459.0250, found 459.0252.
m/z calcd for $C_{16}H_{23}F_3INNaO_4^+$, $[M+CH_3CN+Na]^+$ 500.0515, found 500.0511.

IR (cm^{-1}): 2989, 2944, 1779, 1736, 1445, 1367, 1218, 1159, 1113, 1023, 957, 870, 850, 774, 730.

(Z)-8-Acetoxy-3-iodo-2,6-dimethyloct-6-en-2-yl 2,2,2-trifluoroacetate (5b)

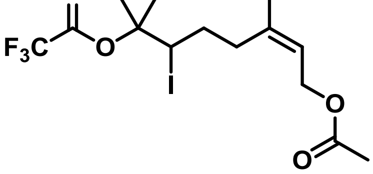

$C_{14}H_{20}F_3IO_4$, 436.2097 g/mol

General procedure D was applied to neryl acetate (**1b**, 57.2 mg, 0.291 mmol). The crude residue was purified by flash column chromatography (PE/EtOAc 100/0 ⟶ 95/05) to afford **5b** (77.8 mg, 61%) as a colorless oil.

NMR ^{1}H (300 MHz, C_6D_6): δ = 5.35 (bt, *J* = 7.3 Hz, 1H, =CH), 4.62 (d, *J* = 7.3 Hz, 2H, CH_2-OAc), 4.25 (dd, *J* = 10.5, 3.0 Hz, 1H, CH-I), 2.13–2.04 (m, 2H, CH_2), 1.73 (s, 3H, Ac), 1.52–1.48 (m, 1H, CH_2), 1.47 (s, 3H, CH_3-C=C), 1.45–1.40 (m, 1H, CH_2), 1.34 (s, 3H, CH_3), 1.31 (s, 3H, CH_3).

NMR ^{13}C (75 MHz, C_6D_6): δ = 169.9 (C=O), 155.8 (q, *J* = 41.7 Hz, CF_3**C**=O), 139.4 (C_q), 122.1 (CH), 114.8 (q, *J* = 287 Hz, CF_3), 89.1 (C_q), 60.7 (OCH_2), 42.0 (CH-I), 33.2 (CH_2), 31.9 (CH_2), 25.1 (CH_3), 22.8 (CH_3), 22.2 (CH_3), 20.5 (CH_3).

NMR ^{19}F (300 Hz, C_6D_6): δ = -75.4.

HRMS (ESI): m/z calcd for $C_{14}H_{20}F_3INaO_4^+$, $[M+Na]^+$ 459.0250, found 459.0250.
m/z calcd for $C_{16}H_{23}F_3INNaO_4^+$, $[M+CH_3CN+Na]^+$ 500.0515, found 500.0529.

IR (cm^{-1}): 2942, 1779, 1737, 1445, 1366, 1218, 1158, 1111, 1022, 956, 870, 850, 774, 729.

(E)-8-Hydroxy-3-iodo-2,6-dimethyloct-6-en-2-yl 2,2,2-trifluoroacetate **(5c)**

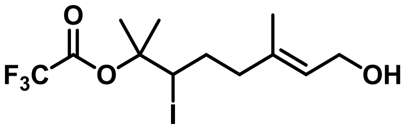

$C_{12}H_{18}F_3IO_3$, 394.1727 g/mol

General procedure D was applied to geraniol (**1c**, 60.4 mg, 0.392 mmol). The crude residue was purified by flash column chromatography (PE/EtOAc 100/0 ⟶ 80/20) to afford **5c** (35.5 mg, 23%) as a colorless oil.

NMR ^{1}H (300 MHz, C_6D_6): δ = 5.39 (bt, *J* = 6.6 Hz, =CH), 4.37 (dd, *J* = 11.3, 2.3 Hz, 1H, CH-I), 3.91 (d, *J* = 6.6 Hz, 2H, CH_2-OH), 2.15–2.06 (m, 1H, CH_2), 1.95–1.82 (m, 1H, CH_2), 1.49 (d, *J* = 4.1 Hz, 1H, CH_2), 1.47–1.38 (m, 1H, CH_2), 1.36 (s, 3H, CH_3-C=C), 1.34 (s, 3H, CH_3), 1.29 (s, 3H, CH_3).

NMR ^{13}C (75 MHz, C_6D_6): δ = 155.9 (q, *J* = 41.7 Hz, CF_3**C**=O), 135.6 (C_q), 126.6 (CH), 114.8 (q, *J* = 287 Hz, CF_3), 89.4 (C_q), 59.0 (OCH_2), 41.8 (CH-I), 39.0 (CH_2), 32.6 (CH_2), 25.2 (CH_3), 22.3 (CH_3), 15.7 (CH_3).

NMR ^{19}F (300 Hz, C_6D_6): δ = -75.4.

HRMS (ESI): *m/z* calcd for $C_{14}H_{21}F_3INNaO_3^+$, $[M+CH_3CN+Na]^+$ 458.0411, found 458.0413.

IR (cm^{-1}): 3320, 2922, 1778, 1670, 1444, 1367, 1218, 1159, 1111, 1000, 869, 846, 774, 729.

(E)-3-Iodo-2,6-dimethyl-8-((4-methylphenyl)sulfonamido)oct-6-en-2-yl 2,2,2-trifluoroacetate (5d)

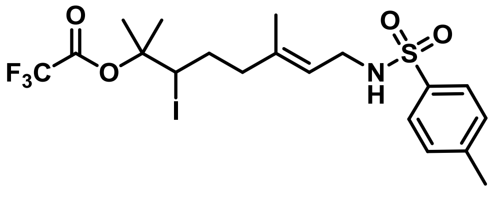

$C_{19}H_{25}F_3INO_4S$, 547.3717 g/mol

General procedure D was applied to *N*-tosyl geranylamine (**1d**, 57.9 mg, 0.188 mmol). The crude residue was purified by flash chromatography on silica gel (PE/EtOAc 100/0 ⟶ 80/20) to afford **5d** (59 mg, 57%) as a colorless oil.

NMR ^{1}H (300 MHz, C_6D_6): δ = 7.83 (d, *J* = 8.1 Hz, 2H, CH_{Ar}), 6.83 (d, *J* = 8.1 Hz, 2H, CH_{Ar}), 5.00 (bt, *J* = 7.0 Hz, 1H, =CH), 4.92 (bt, *J* = 5.7 Hz 1H, NH), 4.29 (dd, *J* = 11.2, 2.2 Hz, 1H, CH-I), 3.44 (bs, 2H, CH_2-NHTs), 2.01–1.90 (m, 1H, CH_2), 1.93 (s, 3H, CH_3-Ar), 1.78–1.65 (m, 1H, CH_2), 1.50 (d, *J* = 3.0 Hz, 1H, CH_2), 1.43–1.37 (m, 1H, CH_2), 1.35 (s, 3H, CH_3), 1.32 (s, 3H, CH_3), 1.23 (s, 3H, CH_3-C=C).

NMR ^{13}C (75 MHz, C_6D_6): δ = 155.9 (q, *J* = 41.2 Hz, CF_3**C**=O), 142.9 (C_q), 138.3 (C_q), 137.8 (C_q), 129.6 (2CH), 127.5 (2CH), 121.4 (CH), 114.8 (q, *J* = 287.6 Hz, CF_3), 89.4 (C_q), 42.1 (CH-I), 40.9 ($NHCH_2$), 38.9 (CH_2), 32.5 (CH_2), 25.1 (CH_3), 22.4 (CH_3), 21.1 (CH_3), 15.7 (CH_3).

NMR ^{19}F (300 Hz,): δ = -75.4.

HRMS (ESI): *m/z* calcd for $C_{19}H_{25}F_3INNaO_4S^+$, $[M+Na]^+$ 570.0393, found 570.0399.
m/z calcd for $C_{21}H_{28}F_3IN_2NaO_4S^+$, $[M+CH_3CN+Na]^+$ 611.0659, found 611.0665.

IR (cm^{-1}): 3278, 2925, 1778, 1369, 1327, 1218, 1157, 1094, 1043, 814, 774, 663.

3-Iodo-2-methyl-6-methyleneoct-7-en-2-yl 2,2,2-trifluoroacetate (5e)

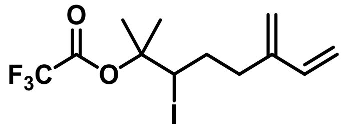

$C_{12}H_{16}F_3IO_2$, 376.1577 g/mol

General procedure D was applied to myrcene (**1e**, 59.3 mg, 0.4353 mmol). The crude residue was purified by flash chromatography on silica gel (PE 100%) to afford **5e** (80.5 mg, 49%) as a colorless oil. *Caution is to be taken since this product is volatile.*

NMR ^{1}H (300 MHz, C_6D_6): δ = 6.17 (dd, *J* = 17.7, 10.9 Hz, 1H, C**H**=CH_2), 5.07 (d, *J* = 17.7 Hz, 1H, CH_2=CH), 4.96 (d, *J* = 10.9 Hz, 1H, C**H**$_2$=CH), 4.94 (bs, 2H, CH_2=C), 4.36 (dd, *J* = 10.9, 2.4 Hz, 1H, CH-I), 2.50–2.41 (m, 1H, CH_2), 2.11–2.01 (m, 1H, CH_2), 1.58–1.49 (m, 2H, CH_2), 1.44 (d, *J* = 3.6 Hz, 1H, CH_2), 1.30 (s, 3H, CH_3), 1.24 (s, 3H, CH_3).

NMR ^{13}C (75 MHz, C_6D_6): δ = 155.8 (q, *J* = 41.2 Hz, CF_3**C**=O), 144.4 (C_q), 138.3 (CH), 117.4 (CH_2), 113.8 (CH_2), 114.8 (q, *J* = 287 Hz, CF_3), 89.1 (C_q), 42.2 (CH-I), 33.5 (CH_2), 31.7 (CH_2), 25.1 (CH_3), 22.3 (CH_3).

NMR ^{19}F (300 Hz, C_6D_6): δ = -75.4.

HRMS (ESI): no ionization could be observed for this compound.

IR (cm^{-1}): 2943, 1778, 1596, 1459, 1367, 1218, 1159, 1113, 992, 901, 870, 774, 731.

7. Procedures and analytical data for allylic-chlorination products

(E)-6-Chloro-3,7-dimethylocta-2,7-dien-1-yl acetate **(6a)**

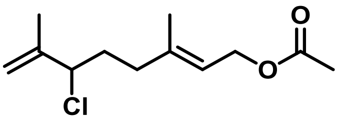

$C_{12}H_{19}ClO_2$, 230.732 g/mol

General procedure E was applied to geranyl acetate (**1a**, 47.1 mg, 0.240 mmol). The crude product was purified by flash chromatography on silica gel (PE/AcOEt 100/0 ⟶ 98/02) to afford **6a** (47 mg, 85%) as a colorless oil. The chemical shifts of the product are in accordance with literature.[6]

NMR ^{1}H (300 MHz, $CDCl_3$): δ = 5.28 (tq, *J* = 7.0, 1.3 Hz, 1H, C**H**=C), 4.94 (m, 1H, C**H**$_2$=C), 4.83 (p, *J* = 1.4 Hz, 1H, C**H**$_2$=C), 4.51 (d, *J* = 7.0 Hz, 2H, CH_2-OAc), 4.25 (t, *J* = 7.0 Hz, 1H, CH-Cl), 2.13–2.01 (m, 2H, CH_2), 1.99 (s, 3H, Ac), 1.96–1.82 (m, 2H, CH_2), 1.74 (s, 3H, C**H**$_3$-C=CH_2), 1.64 (s, 3H, C**H$_3$**-C=CH).

NMR ^{13}C (75 MHz, $CDCl_3$): δ = 171.0 (C=O), 144.2 (C_q), 140.5 (C_q), 119.4 (CH), 114.3 (CH_2), 66.1 (CH-Cl), 61.2 (OCH_2), 36.5 (CH_2), 34.4 (CH_2), 21.0 (CH_3), 17.0 (CH_3), 16.5 (CH_3).

HRMS (ESI): m/z calcd for $C_{10}H_{16}Cl^+$, [M–OAc]$^+$ 171,0935, found 171,0942.

IR (cm^{-1}): 2950, 1737, 1443, 1366, 1229, 1022, 955, 907, 791, 678.

(Z)-6-Chloro-3,7-dimethylocta-2,7-dien-1-yl acetate (6b)

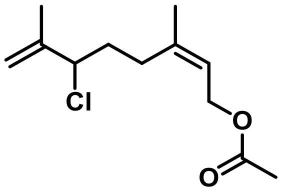

$C_{12}H_{19}ClO_2$, 230.732 g/mol

General procedure E was applied to neryl acetate (**1b**, 61 mg, 0.311 mmol). The crude residue was purified by flash chromatography on silica gel (PE/EtOAc 100/0 ⟶ 98/02) to afford **6b** (55 mg, 76%) as a colorless oil. The chemical shifts of the product are in accordance with literature.[7]

NMR ^{1}H (300 MHz, $CDCl_3$): δ = 5.44 (bt, *J* = 7.2 Hz, 1H, C**H**=C), 5.07 (bs, 1H, C**H**$_2$=C), 4.96 (bt, *J* = 1.4 Hz, 1H, C**H**$_2$=C), 4.61 (d, *J* = 7.2 Hz, 2H, CH_2-OAc), 4.36 (dd, *J* = 8.1, 6.3 Hz, 1H, CH-Cl), 2.26–2.20 (m, 2H, CH_2), 2.09 (s, 3H, Ac), 2.02–1.92 (m, 2H, CH_2), 1.86 (s, 3H, C**H**$_3$-C=CH_2), 1.81 (s, 3H, C**H$_3$**-C=CH).

NMR ^{13}C (75 MHz, $CDCl_3$): δ = 171.0 (C=O), 144.2 (C_q), 141.0 (C_q), 120.4 (CH), 114.4 (CH_2), 66.1 (CH-Cl), 60.9 (OCH_2), 34.8 (CH_2), 29.3 (CH_2), 23.3 (CH_3), 21.1 (CH_3), 17.1 (CH_3).

HRMS (ESI): m/z calcd for $C_{10}H_{16}Cl^+$, [M–OAc]$^+$ 171,0935, found 171,0938.

[6] V. P. Demertzidou, S. Pappa, V. Sarli, A. L. Zografos, *J. Org. Chem.* **2017**, *82*, 8710–8715.
[7] L. Novák, L. Poppe, C. Szántay, É. Szabó, *Synthesis*, **1985**, *10*, 939–941.

IR (cm^{-1}): 2951, 1736, 1445, 1377, 1229, 1021, 956, 907, 810.

***(E)-6-Chloro-3,7-dimethylocta-2,7-dien-1-ol* (6c)**

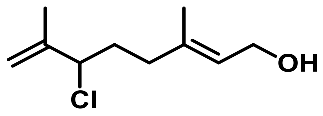

$C_{10}H_{17}ClO$, 188.6950 g/mol

General procedure E was applied to geraniol (**1c**, 65 mg, 0.421 mmol). The crude product was purified by flash chromatography on silica gel (PE/AcOEt 100/0 ⟶ 80/20) to afford **6c** (40.3 mg, 51%) as a colorless oil. The chemical shifts of the product are in accordance with literature.[8]

NMR ^{1}H (300 MHz, CDCl$_3$): δ = 5.44 (bt, *J* = 6.8 Hz, 1H, C**H**=C), 5.03 (s, 1H, C**H**$_2$=C), 4.93 (s, 1H, C**H**$_2$=C), 4.35 (t, *J* = 7.0 Hz, 1H, CH-Cl), 4.17 (bd, *J* = 6.8 Hz, 2H, CH$_2$-OH), 2.17–1.91 (m, 4H, CH$_2$), 1.83 (s, 3H, C**H**$_3$-C=CH$_2$), 1.70 (s, 3H, C**H$_3$**-C=CH).

NMR ^{13}C (75 MHz, CDCl$_3$): δ = 144.2 (C$_q$), 138.0 (C$_q$), 124.4 (CH), 114.3 (CH$_2$), 66.2 (CH-Cl), 59.3 (OCH$_2$), 36.5 (CH$_2$), 34.6 (CH$_2$), 17.0 (CH$_3$), 16.3 (CH$_3$).

HRMS (ESI): m/z calcd for $C_{20}H_{34}Cl_2NaO_2^+$, [M+Na]$^+$ 399.1828, found 399.1831.

IR (cm^{-1}): 3320, 2948, 2919, 2860, 1441, 1376, 1370, 1222, 1166, 997, 905, 790.

(E)-N-(6-Chloro-3,7-dimethylocta-2,7-dien-1-yl)-4-methylbenzenesulfonamide (6d)

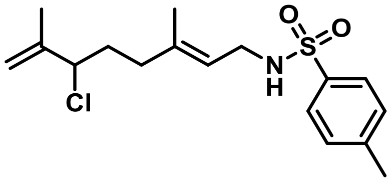

$C_{17}H_{24}ClNO_2S$, 341.894 g/mol

General procedure E was applied to *N*-tosyl geranylamine (**1d**, 54 mg, 0.176 mmol). The crude residue was purified by flash chromatography on silica gel (PE/EtOAc 100/0 ⟶ 85/15) and the collected fraction was purified by preparative TLC (Heptane/EtOAc 85/15) to afford **6d** (36.1 mg, 60%) as a colorless oil.

NMR ^{1}H (300 MHz, CDCl$_3$): δ = 7.77 (d, *J* = 8.0 Hz, 2H, CH$_{Ar}$), 7.33 (d, *J* = 8.0 Hz, 2H, CH$_{Ar}$), 5.08 (bt, *J* = 7.0 Hz, 1H, C**H**=C), 4.99 (bs, 1H, C**H**$_2$=C), 4.91 (t, *J* = 1.3 Hz, 1H, C**H**$_2$=C), 4.52 (bs, 1H, NH), 4.27 (t, *J* = 7.0 Hz, 1H, CH-Cl), 3.59 (t, *J* = 5.7 Hz, 2H, CH$_2$-NHTs), 2.46 (s, 3H, CH$_3$-Ar), 2.06–1.95 (m, 2H, CH$_2$), 1.88–1.81 (m, 2H, CH$_2$), 1.80 (s, 3H, C**H**$_3$-C=CH$_2$), 1.58 (s, 3H, C**H$_3$**-C=CH).

[8] L. Novák, L. Poppe, C. Szántay, É. Szabó, *Synthesis*, **1985**, *10*, 939–941.

NMR ^{13}C (75 MHz, CDCl$_3$): δ = 144.1 (C_q), 143.5 (C_q), 139.3 (C_q), 137.1 (C_q), 129.7 (2CH), 127.2 (2CH), 119.8 (CH), 114.4 (CH_2), 66.1 (CH-Cl), 40.9 (CH_2), 36.3 (CH_2), 34.4 (CH_2), 21.6 (CH_3), 17.0 (CH_3), 16.3 (CH_3).

HRMS (ESI): m/z calcd for $C_{17}H_{25}ClNO_2S^+$, $[M+H]^+$ 342.1289, found 342.1274
m/z calcd for $C_{19}H_{27}ClN_2NaO_2S^+$, $[M+CH_3CN+Na]^+$ 405.1374, found 405.1393
m/z calcd for $C_{34}H_{49}Cl_2N_2O_4S_2^+$, $[2M+H]^+$ 683.2505, found 683.2504

IR (cm^{-1}): 3280, 2923, 1435, 1325, 1156, 1093, 1044, 907, 814, 662.

3-Chloro-2-methyl-6-methyleneocta-1,7-diene (6e)

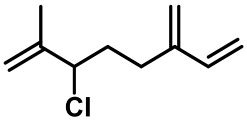

$C_{10}H_{15}Cl$, 170.680 g/mol

General procedure E was applied to myrcene (**1e**, 63 mg, 0.463 mmol). The crude residue was purified by flash chromatography on silica gel (PE 100%) to afford **6e** (45.2 mg, 57%) as a colorless oil. The chemical shifts of the product are in accordance with literature.[9] *Caution is to be taken since this product is volatile.*

NMR ^{1}H (300 MHz, CDCl$_3$): δ = 6.34 (dd, *J* = 17.7, 10.7 Hz, 1H, C**H**=CH$_2$), 5.23 (d, *J* = 17.7 Hz, 1H, C**H**$_2$=CH), 5.12–5.05 (m, 4H, C**H$_2$**=CH, C**H$_2$**=C, C**H$_2$**=CCH$_3$), 4.93 (bt, *J* = 1.5 Hz, 1H, C**H$_2$**=CCH$_3$), 4.40 (dd, *J* = 8.0, 6.3 Hz, 1H, CH-Cl), 2.47–2.37 (m, 1H, CH_2), 2.33–2.22 (m, 1H, CH_2), 2.10–1.98 (m, 2H, CH_2), 1.85 (s, 3H, CH_3).

NMR ^{13}C (75 MHz, CDCl$_3$): δ = 144.9 (C_q), 144.4 (C_q), 138.5 (CH), 116.5 (CH_2), 114.2 (CH_2), 113.6 (CH_2), 66.4 (CH-Cl), 35.1 (CH_2), 28.6 (CH_2), 17.2 (CH_3).

HRMS (ESI): no ionization could be observed for this compound.

IR (cm^{-1}): 2955, 1646, 1595, 1448, 1376, 991, 898, 796.

[9] J. A. Tunge; S. R. Mellegaard; *Org. Lett.* **2004**, *6*, 1205−1207.

8. Copies of ^{1}H and ^{13}C NMR spectra for starting materials

(E)-N-(3,7-Dimethylocta-2,6-dien-1-yl)-4-methylbenzenesulfonamide (1d)

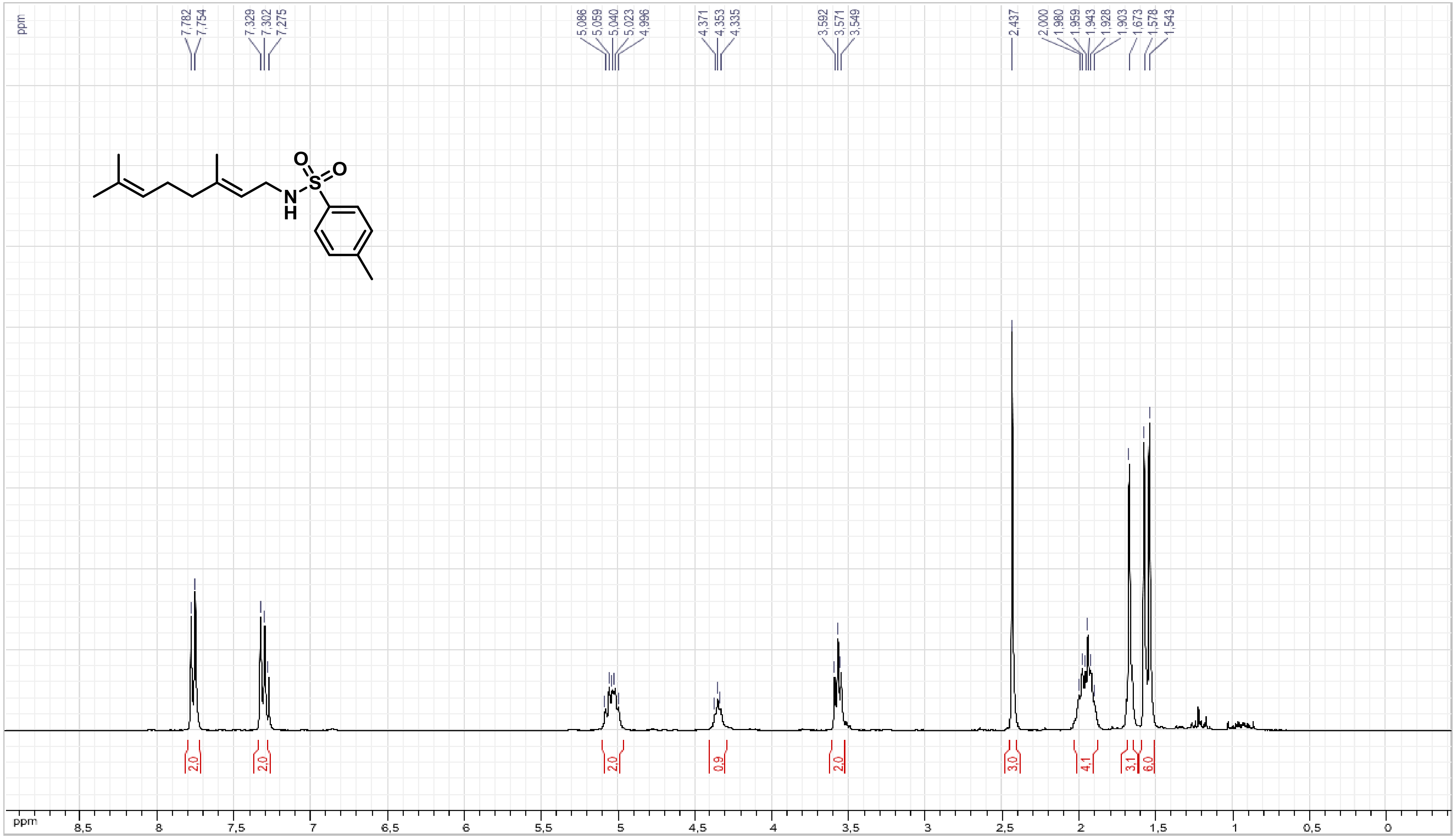

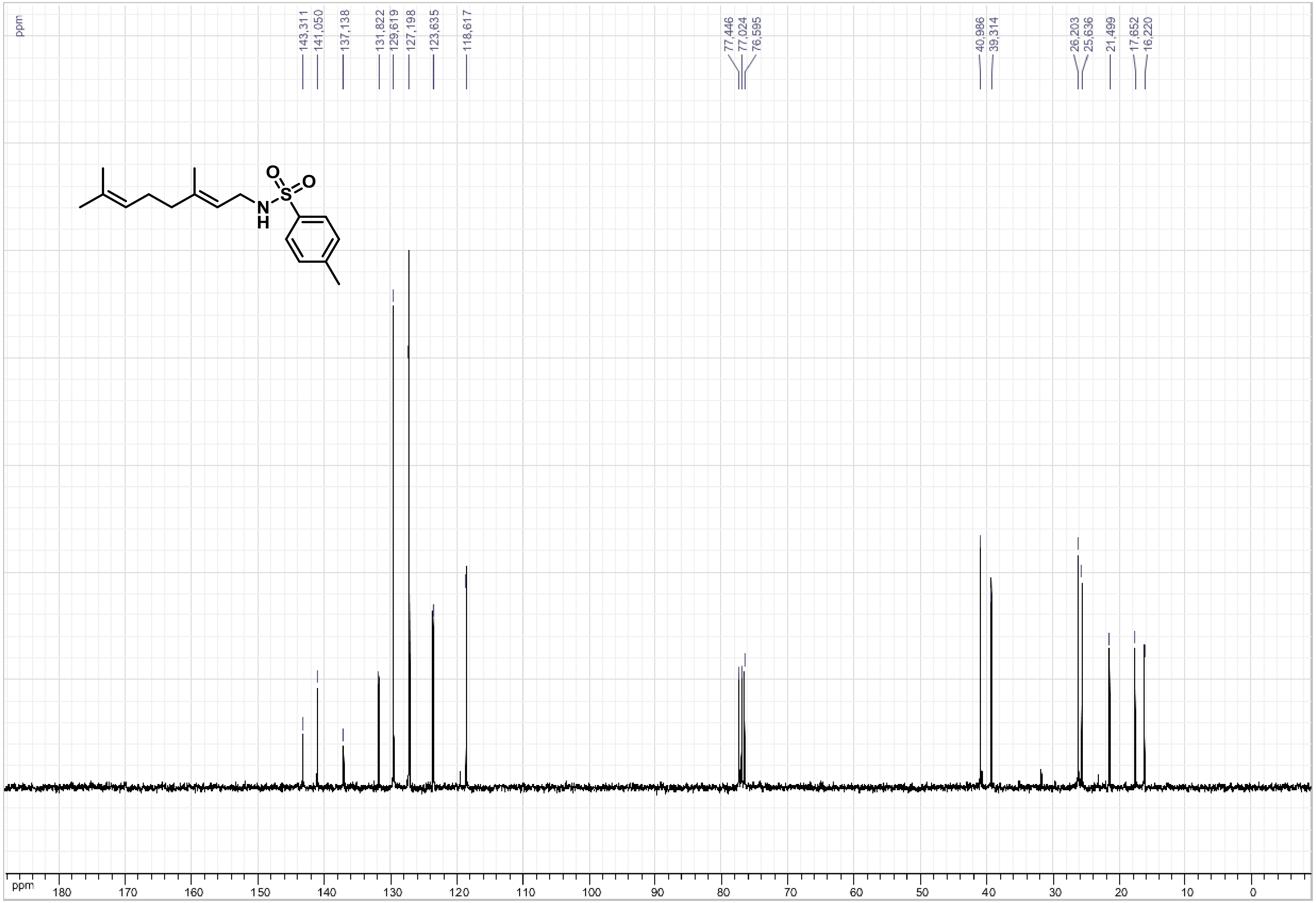
ppm
143,311
141,050
137,138
131,822
129,619
127,198
123,635
118,617
77,446
77,024
76,595
40,986
39,314
26,203
25,636
21,499
17,652
16,220
O
S
O
N
H
ppm
180
170
160
150
140
130
120
110
100
90
80
70
60
50
40
30
20
10
0

9. Copies of ^{1}H and ^{13}C NMR spectra for halogenated products

(E)-6,7-Dibromo-3,7-dimethyloct-2-en-1-yl acetate **(2a)**

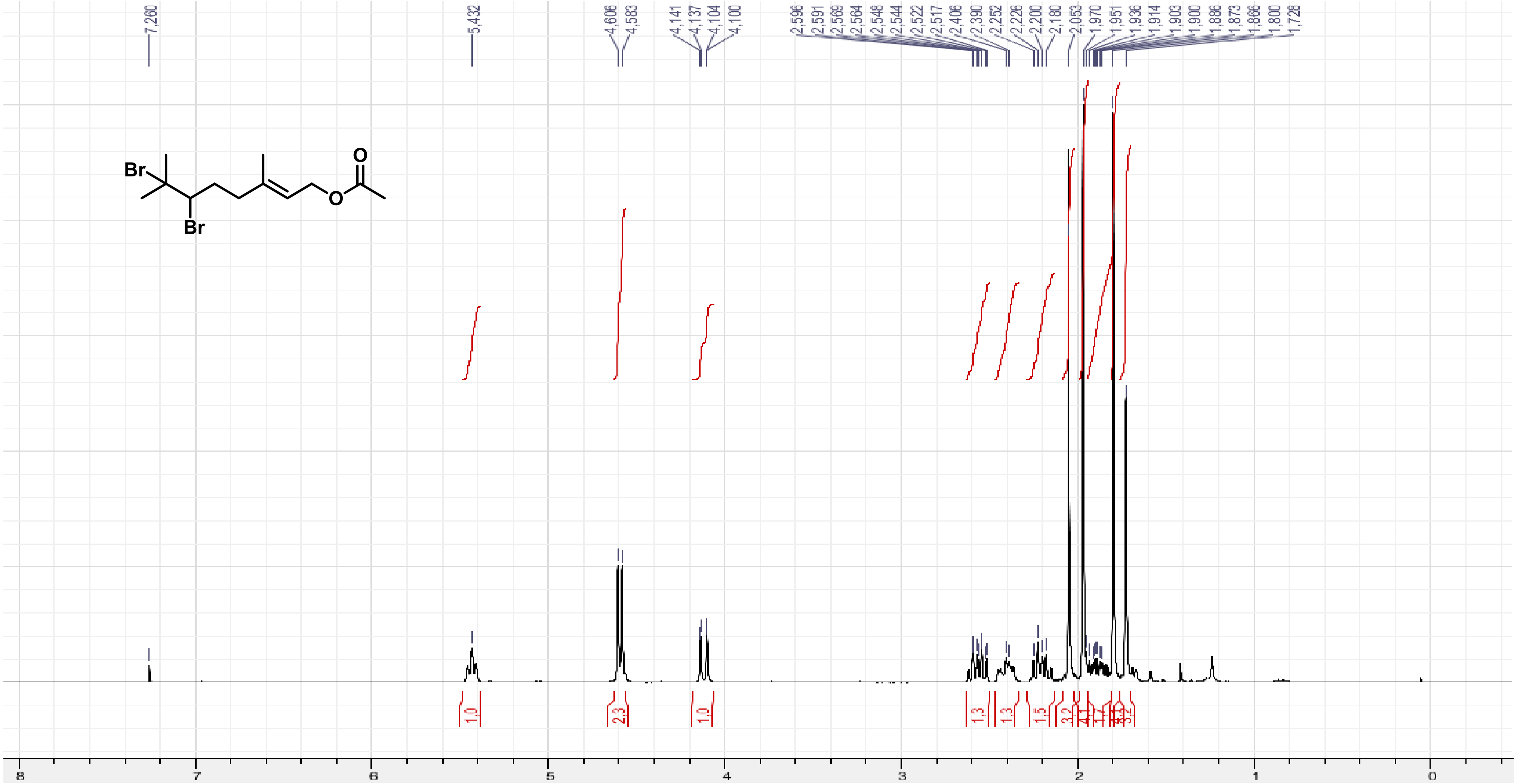

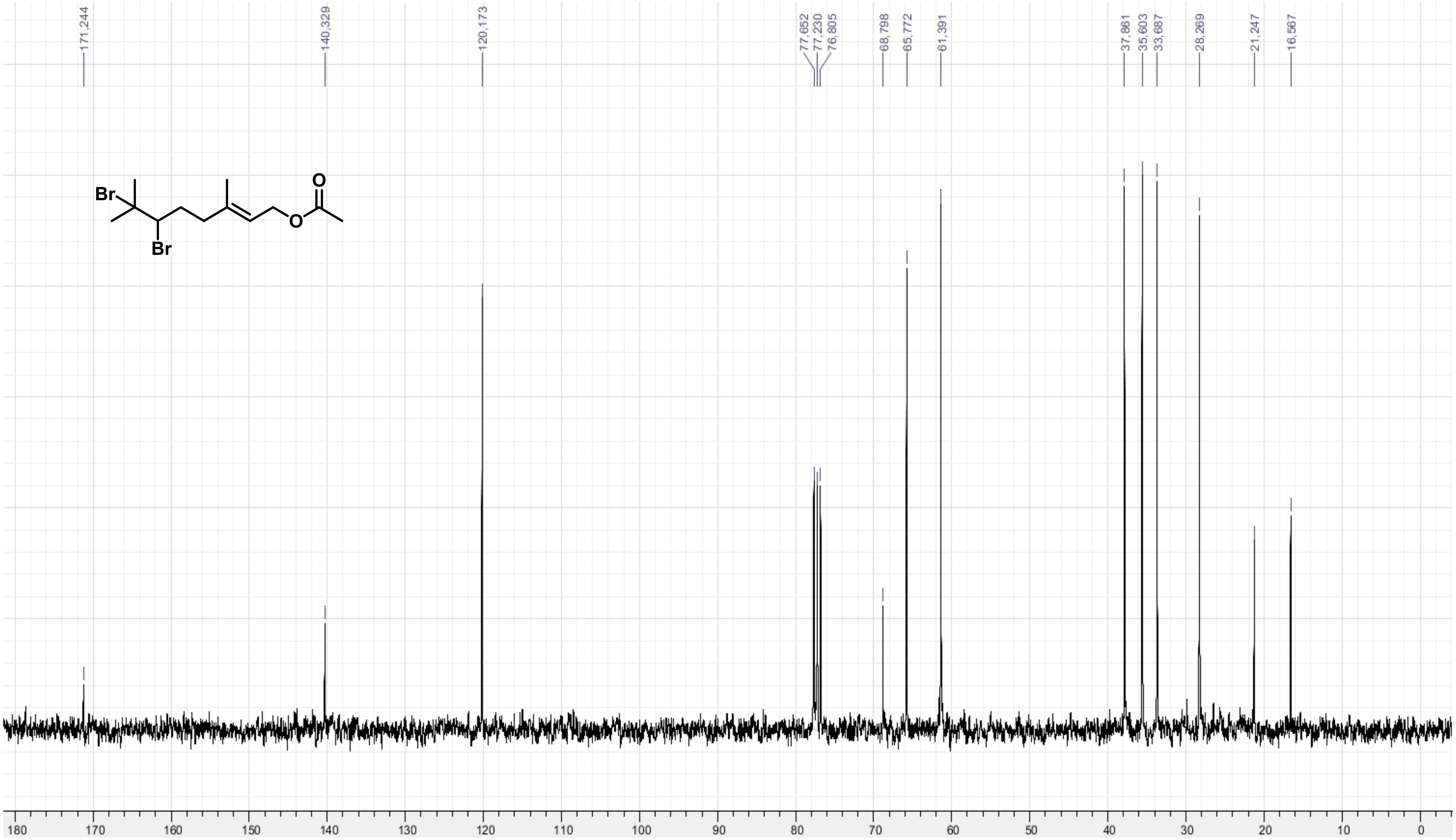
171.244
140.329
120.173
77.652
77.230
76.805
68.798
65.772
61.391
37.861
35.603
33.687
28.269
21.247
16.567
Br
Br
O
O
180
170
160
150
140
130
120
110
100
90
80
70
60
50
40
30
20
10
0

(Z)-6,7-Dibromo-3,7-dimethyloct-2-en-1-yl acetate (2b)

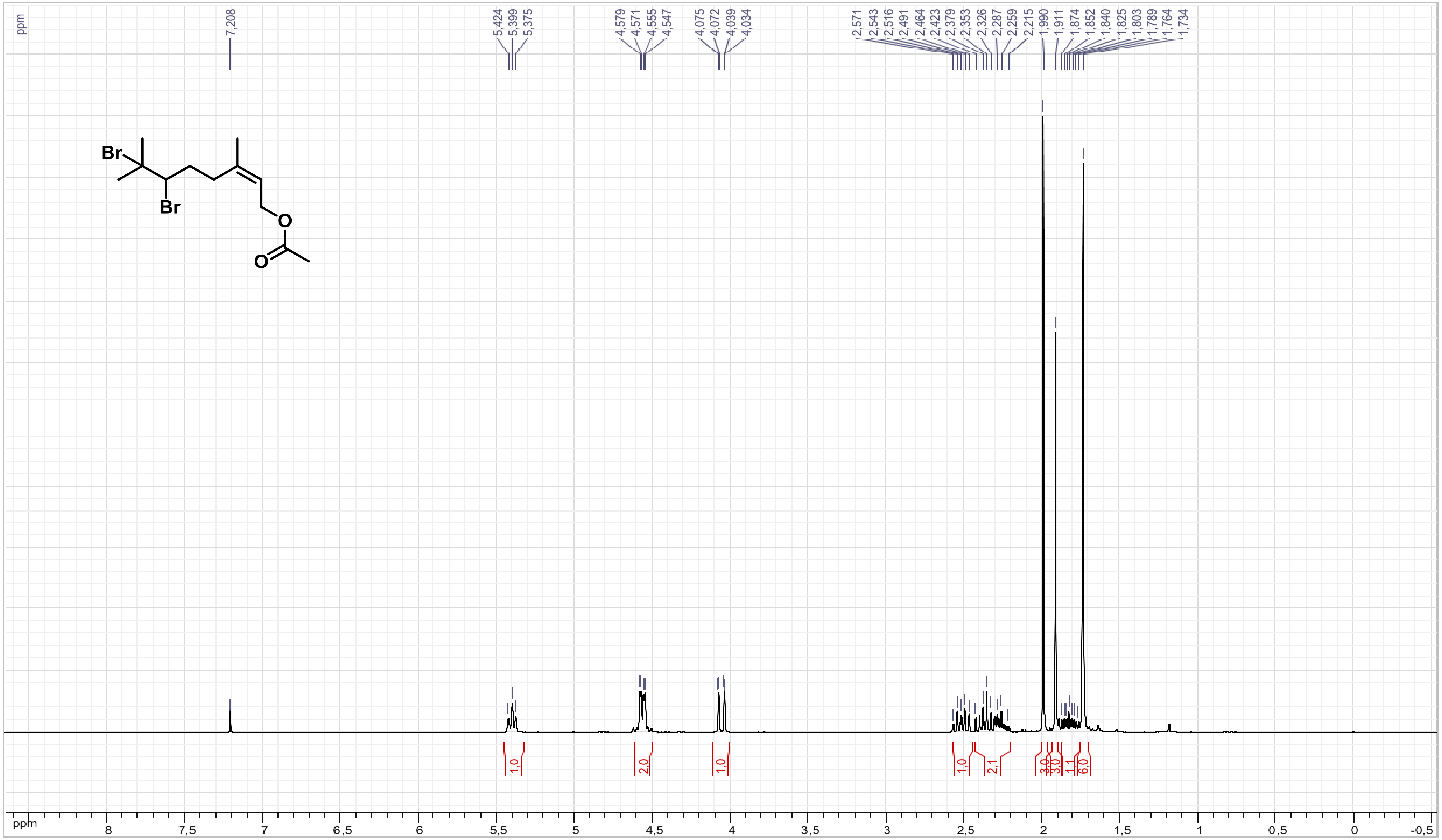

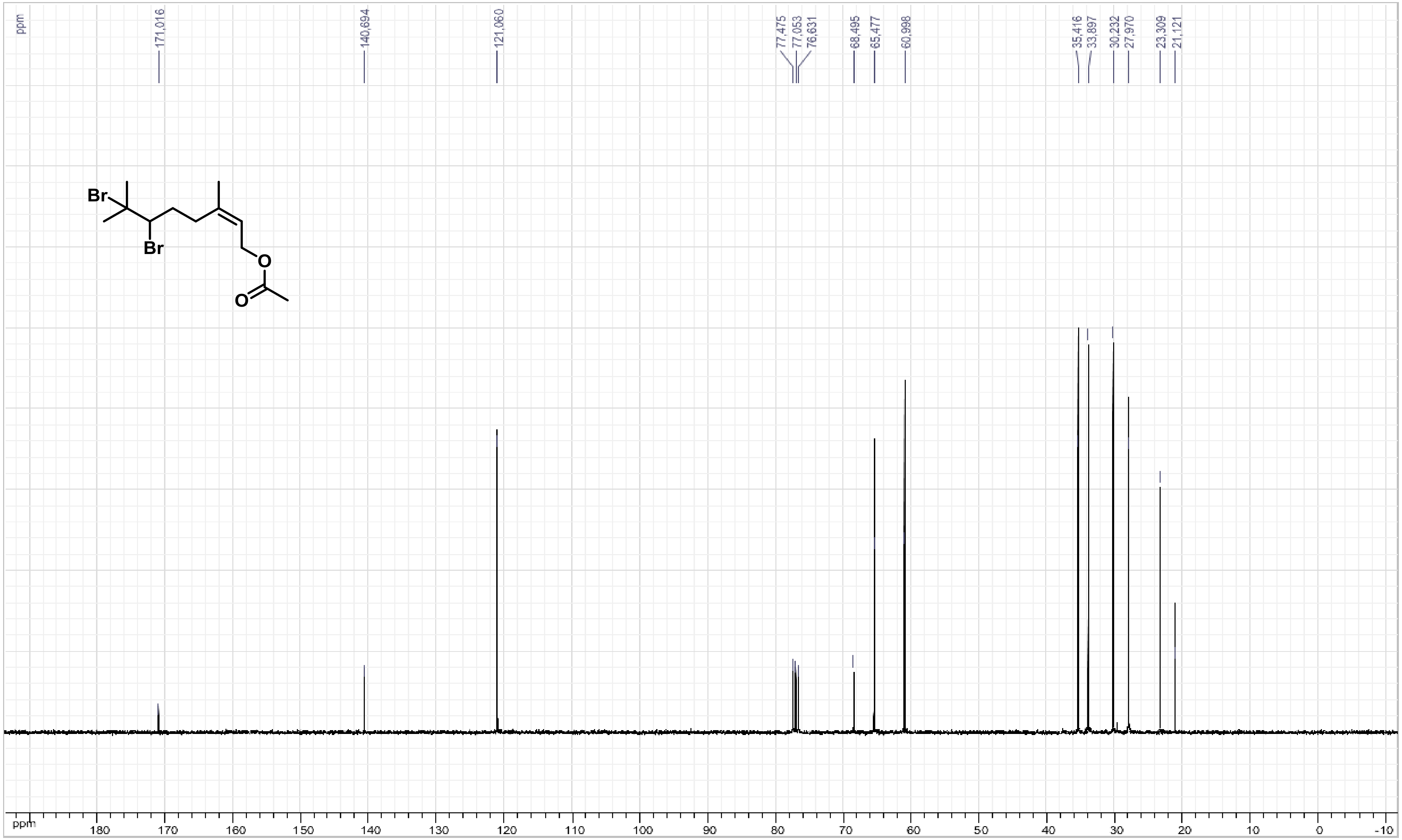
ppm
171,016
140,694
121,060
77,475
77,053
76,631
68,495
65,477
60,998
35,416
33,897
30,232
27,970
23,309
21,121
Br
Br
O
O
ppm
180 170 160 150 140 130 120 110 100 90 80 70 60 50 40 30 20 10 0 -10

(E)-6,7-Dibromo-3,7-dimethyloct-2-en-1-ol (2c)

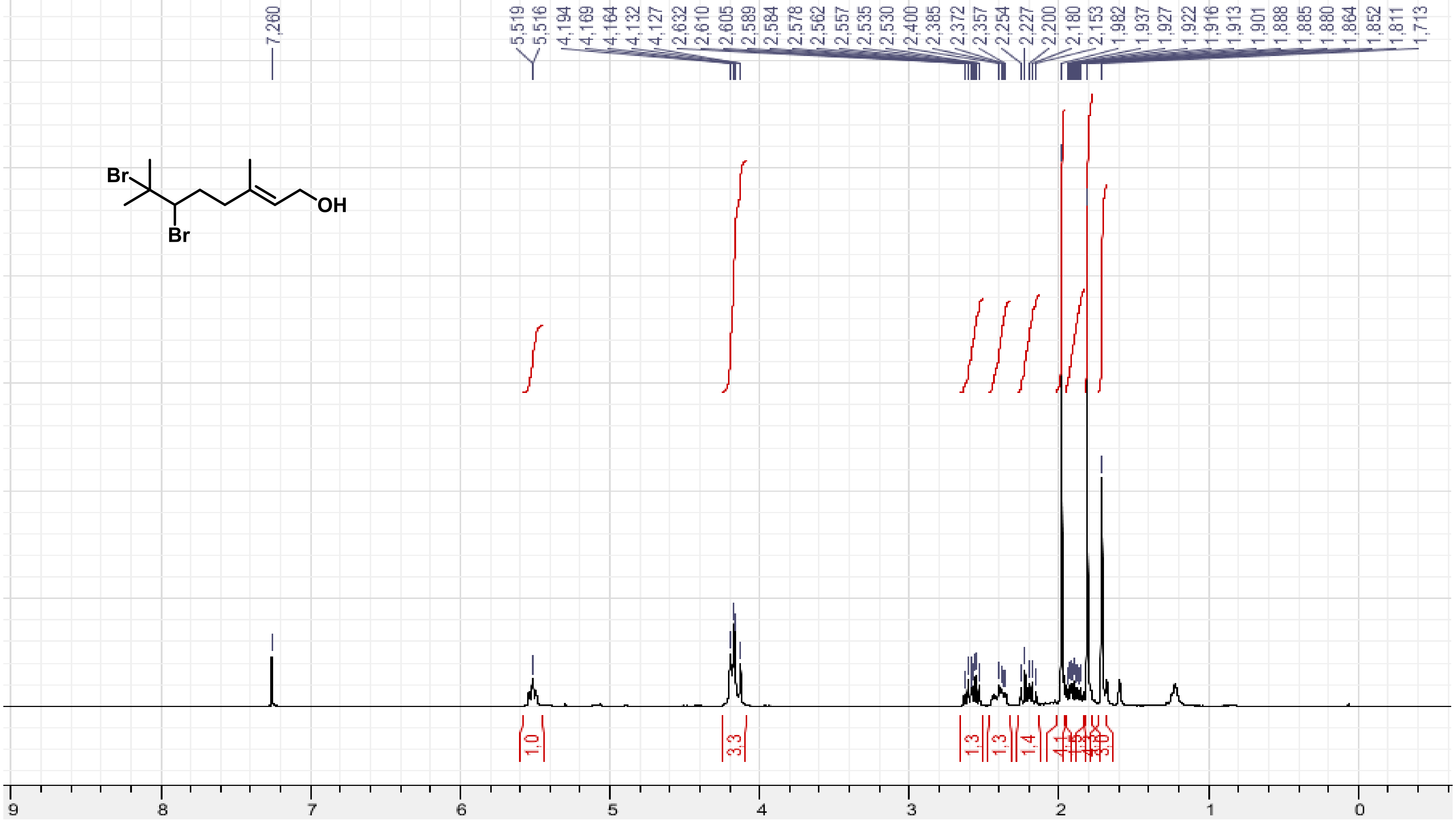

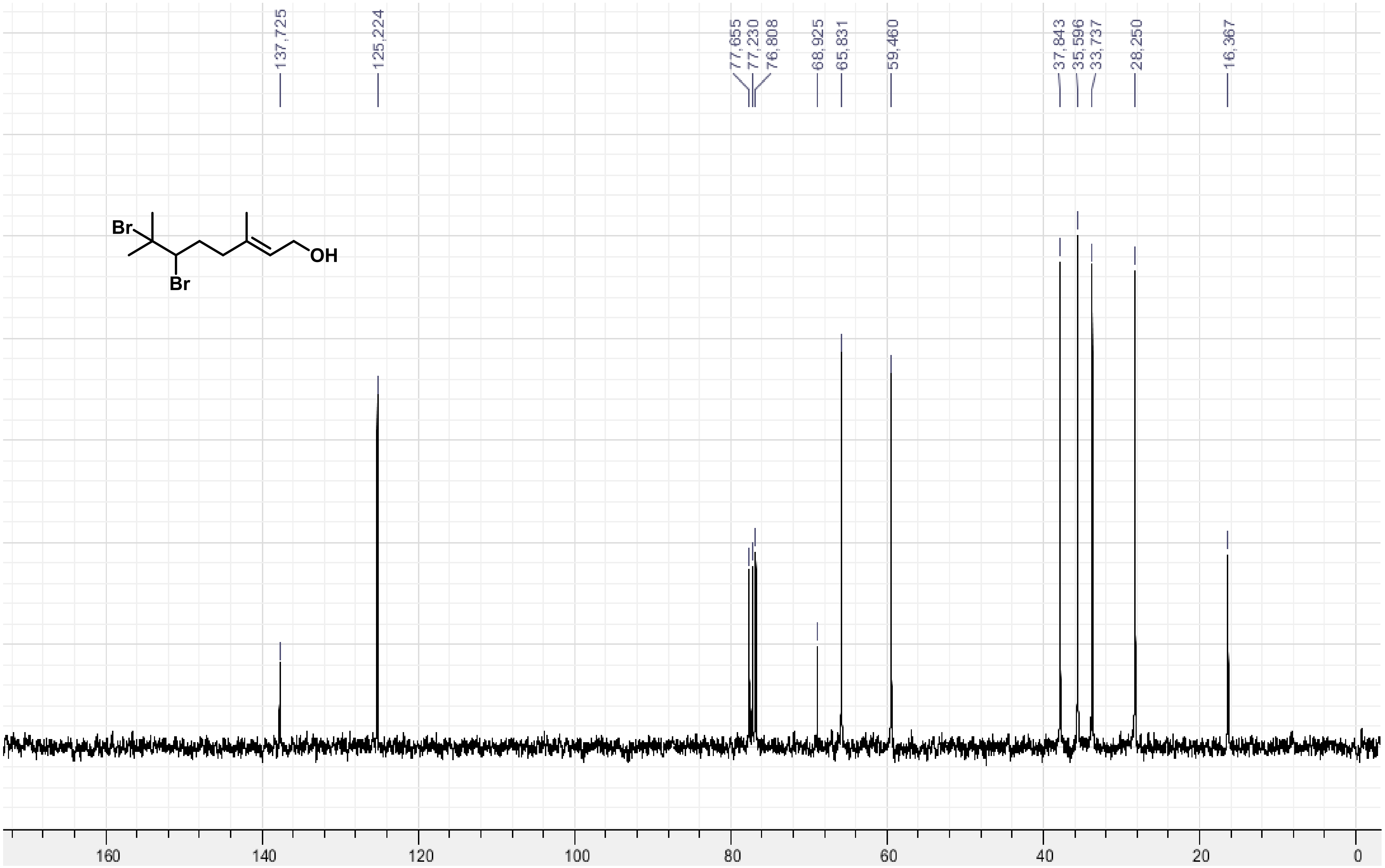
137.725
125.224
77.655
77.230
76.808
68.925
65.831
59.460
37.843
35.596
33.737
28.250
16.367
Br
Br
OH
160
140
120
100
80
60
40
20
0

(E)-N-(6,7-Dibromo-3,7-dimethyloct-2-en-1-yl)-4-methylbenzenesulfonamide (2d)

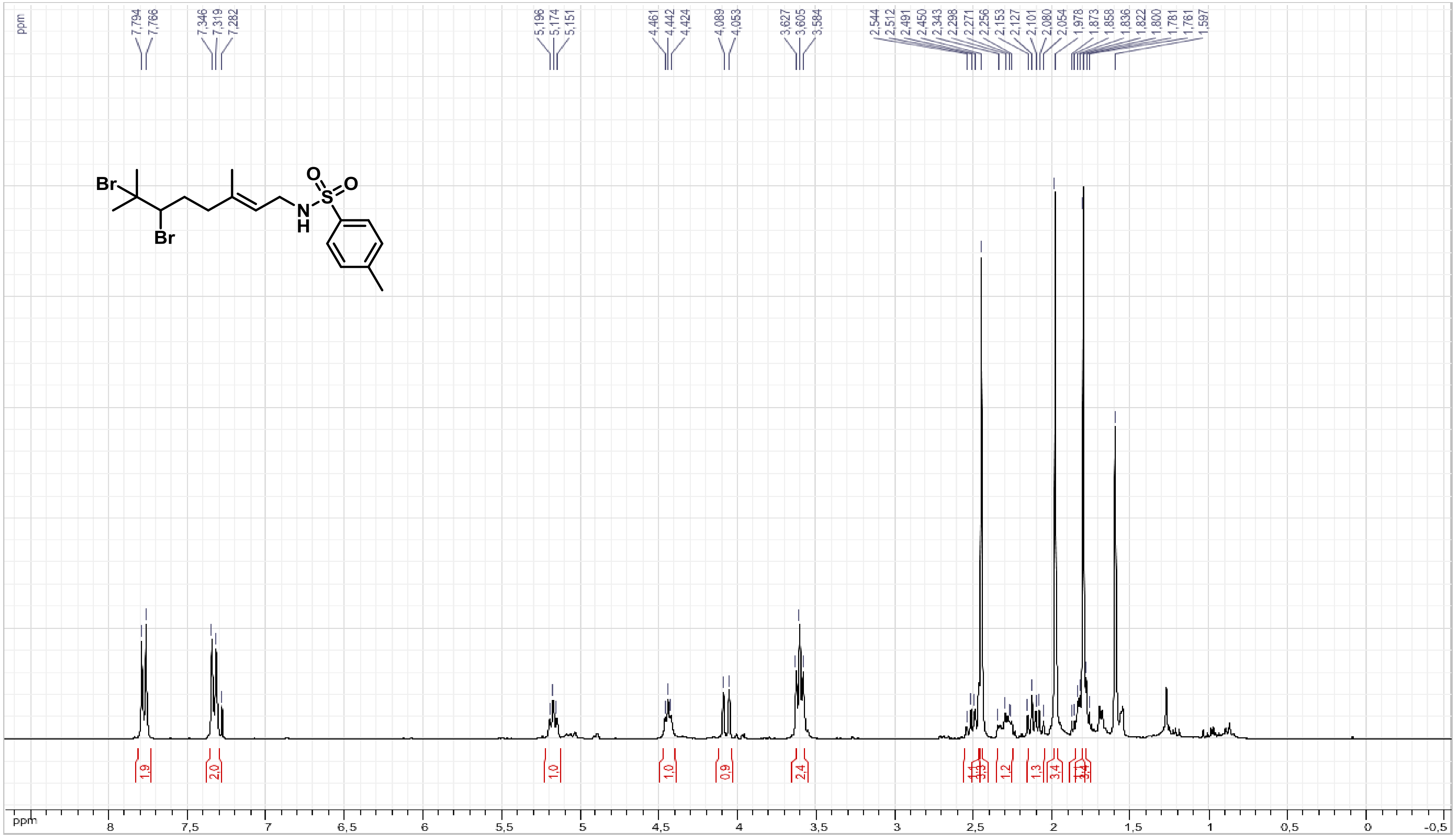

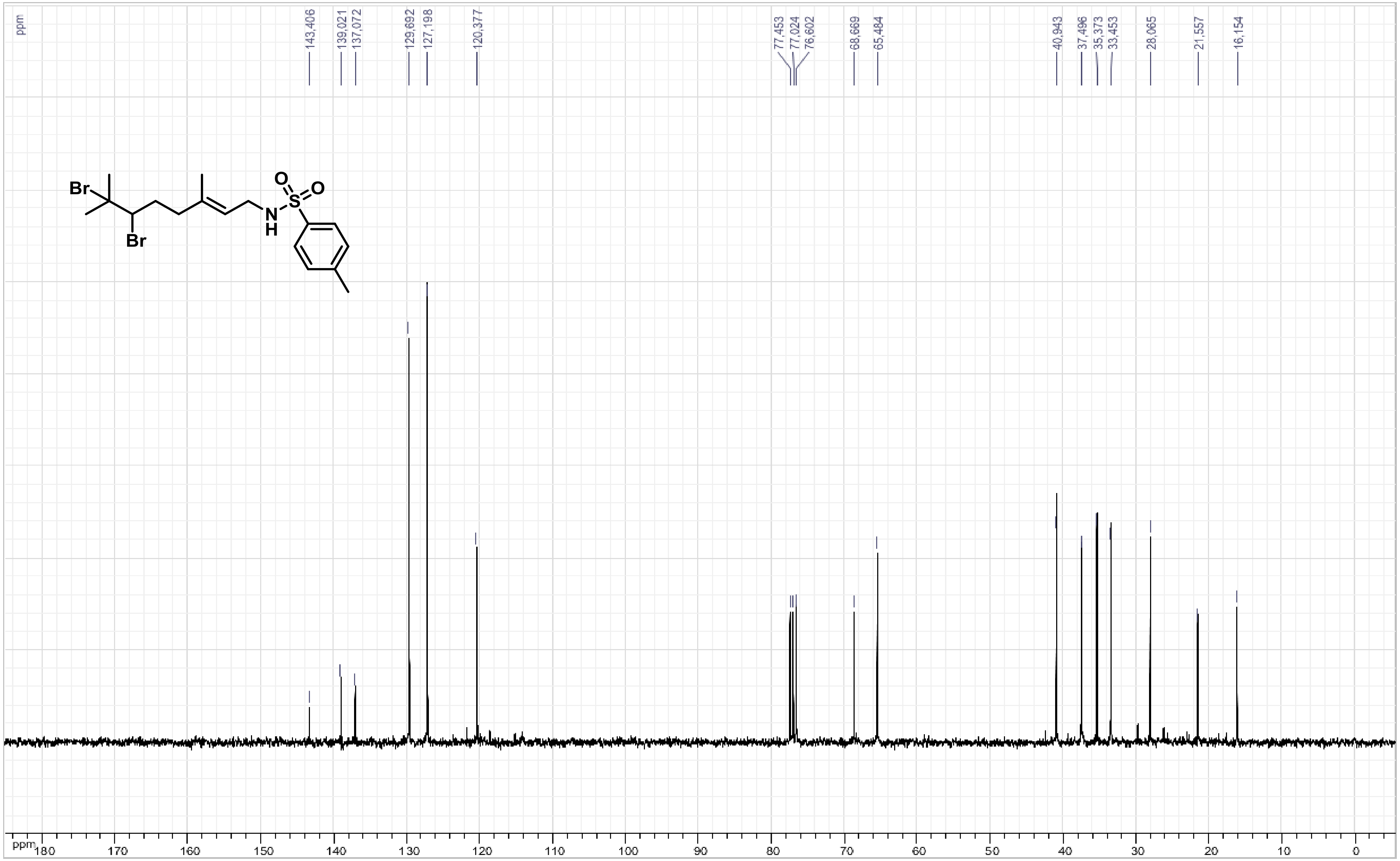
ppm
143,406
139,021
137,072
129,692
127,198
120,377
77,453
77,024
76,602
68,669
65,484
40,943
37,496
35,373
33,453
28,065
21,557
16,154
Br
Br
O
O
S
N
H
ppm 180 170 160 150 140 130 120 110 100 90 80 70 60 50 40 30 20 10 0

6,7-Dibromo-7-methyl-3-methyleneoct-1-ene (2e)

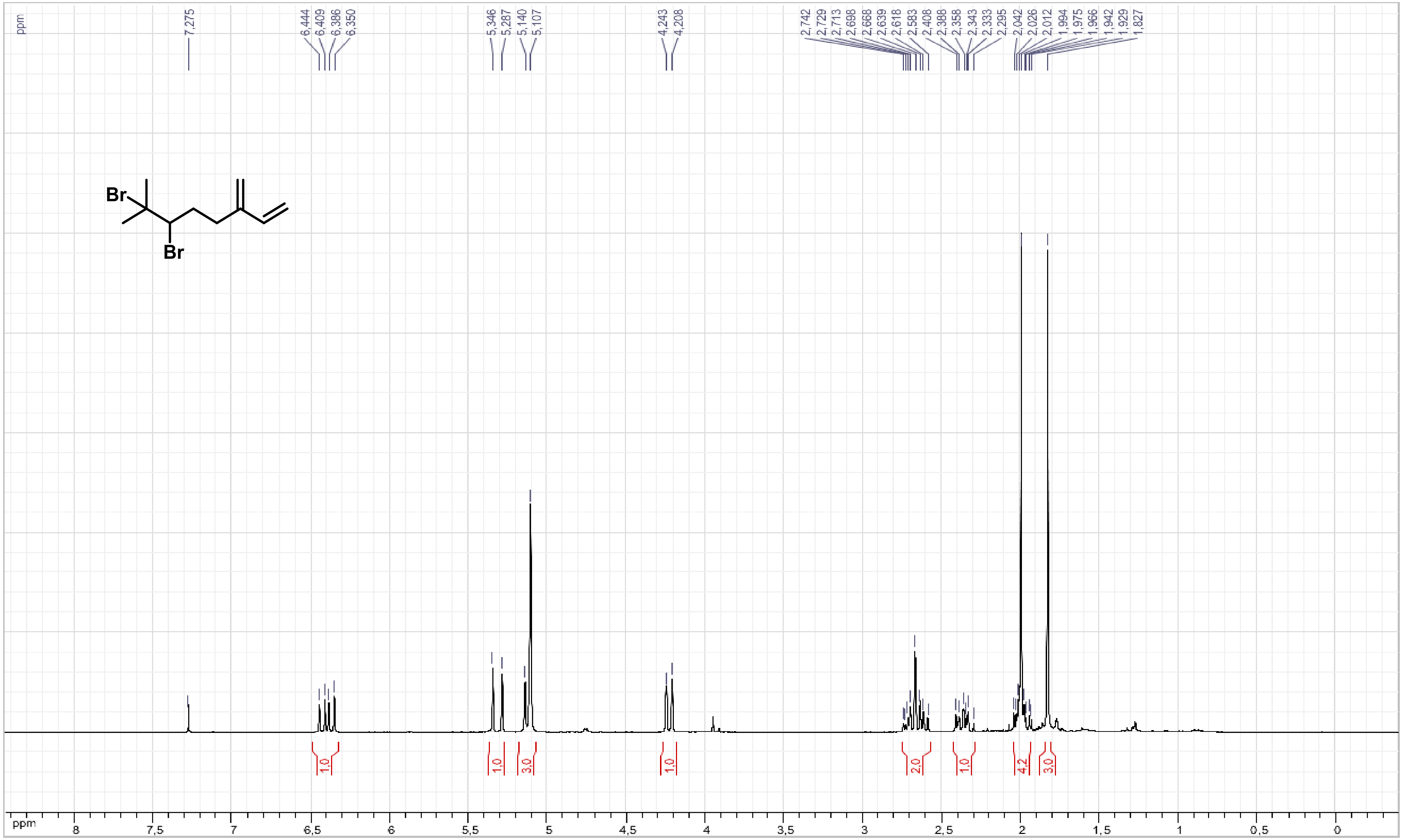

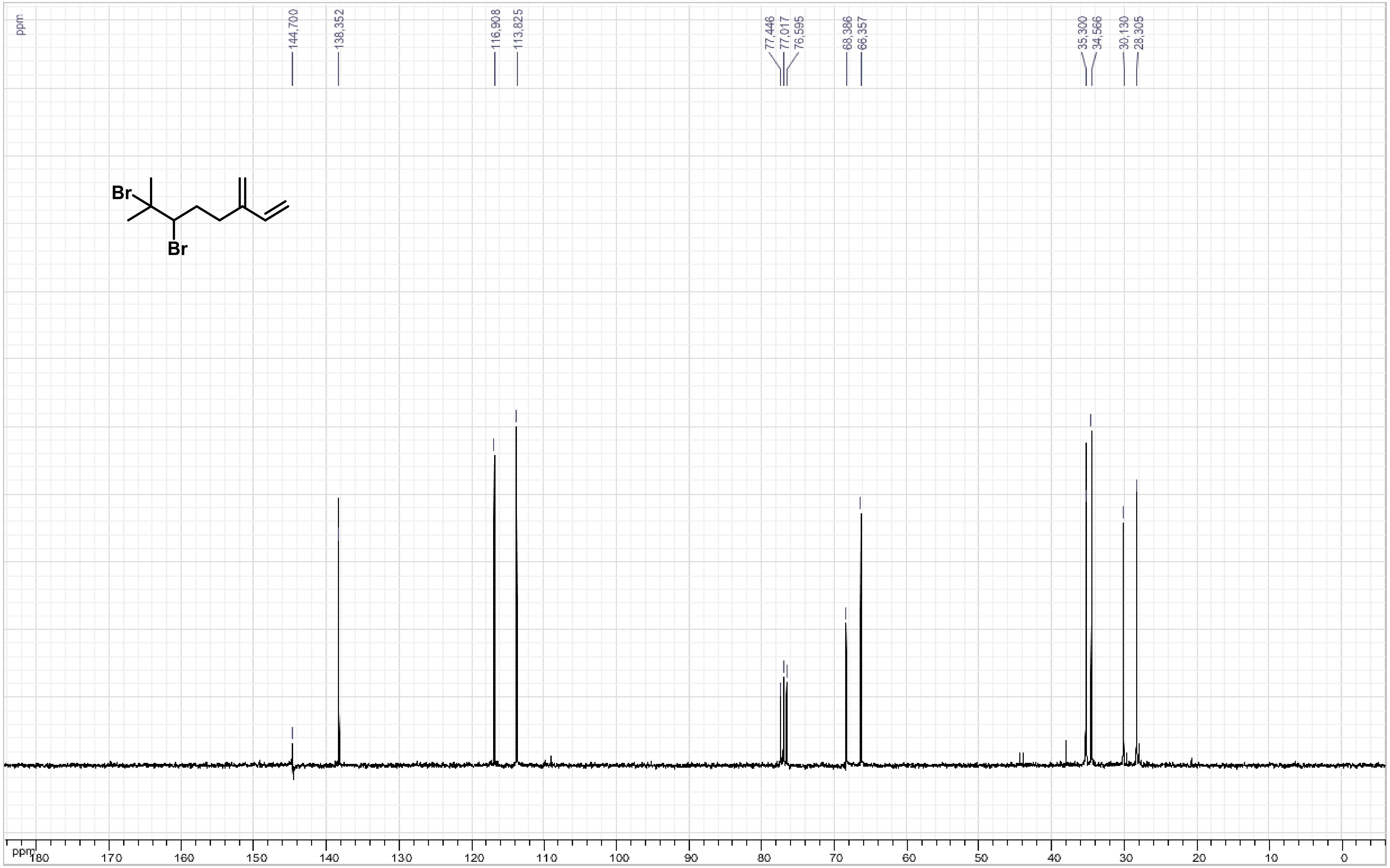
ppm
144.700
138.352
116.908
113.825
77.446
77.017
76.595
68.386
66.357
35.300
34.566
30.130
28.305
Br
Br
ppm 180 170 160 150 140 130 120 110 100 90 80 70 60 50 40 30 20 10 0

(E)-8-Acetoxy-3-bromo-2,6-dimethyloct-6-en-2-yl 2,2,2-trifluoroacetate **(3a)**

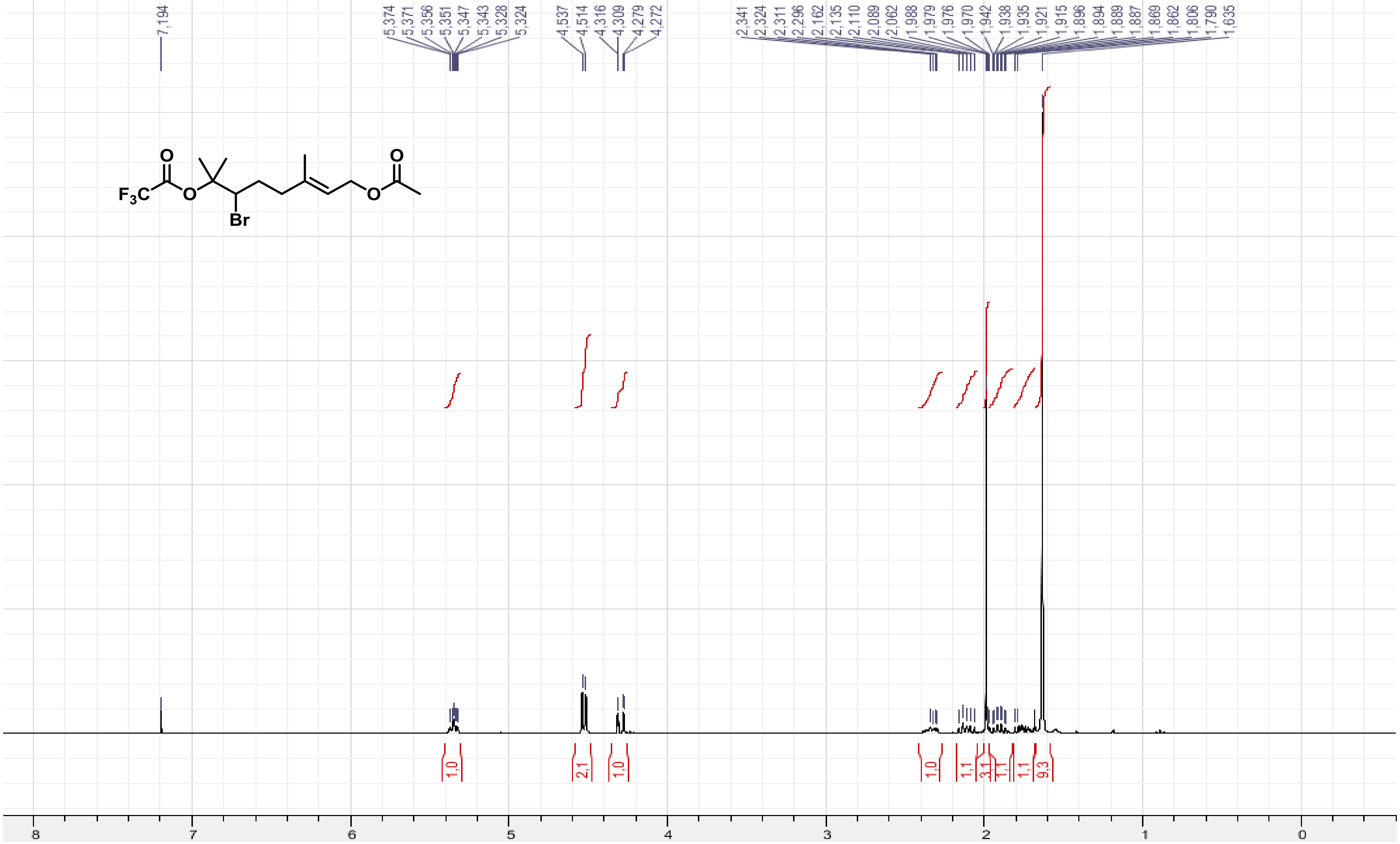

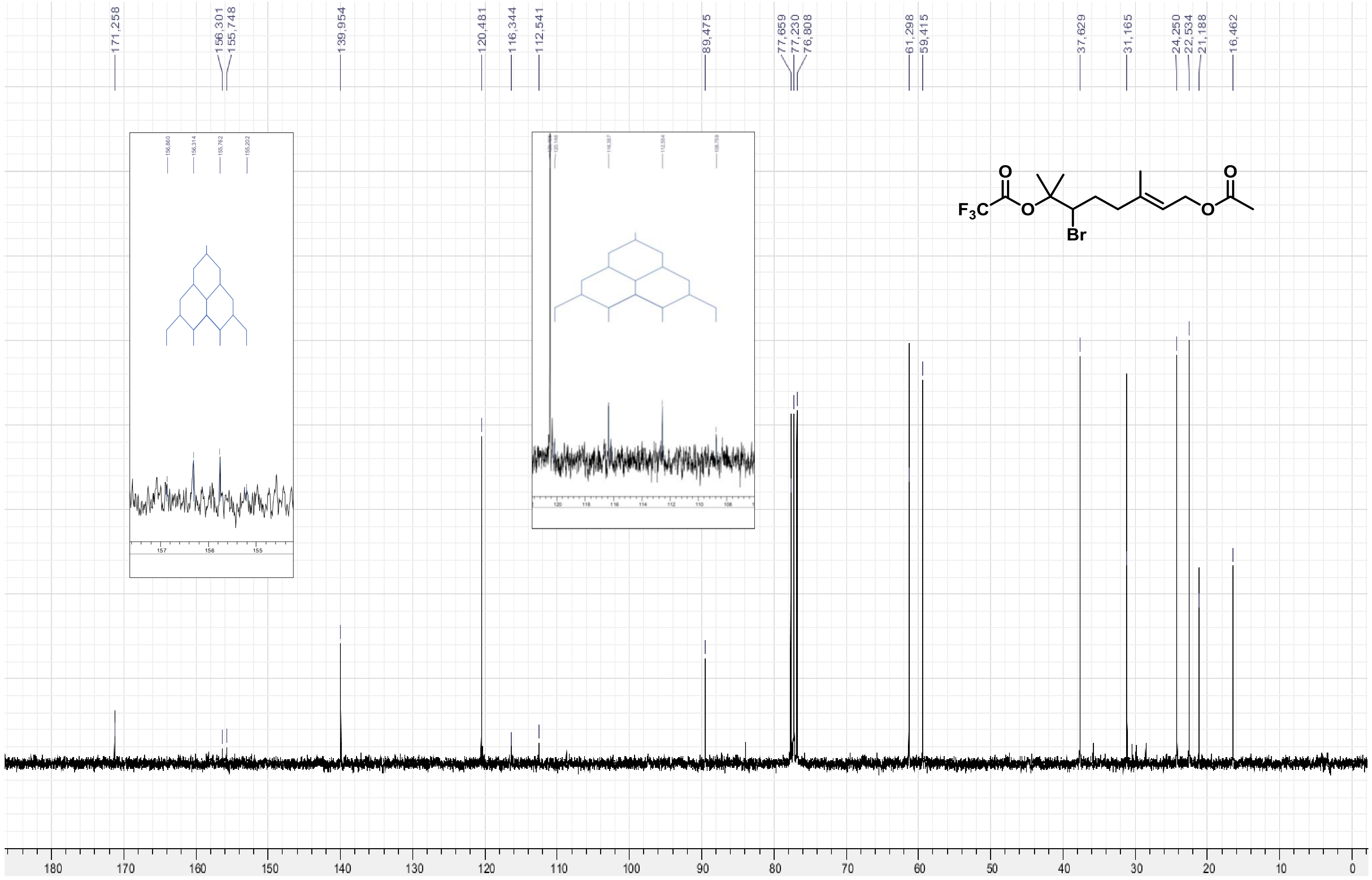

171,258
156,301
155,748
139,954
120,481
116,344
112,541
89,475
77,659
77,230
76,808
61,298
59,415
37,629
31,165
24,250
22,534
21,188
16,462
F3C
O
Br
180
170
160
150
140
130
120
110
100
90
80
70
60
50
40
30
20
10
0

(Z)-8-Acetoxy-3-bromo-2,6-dimethyloct-6-en-2-yl 2,2,2-trifluoroacetate (3b)

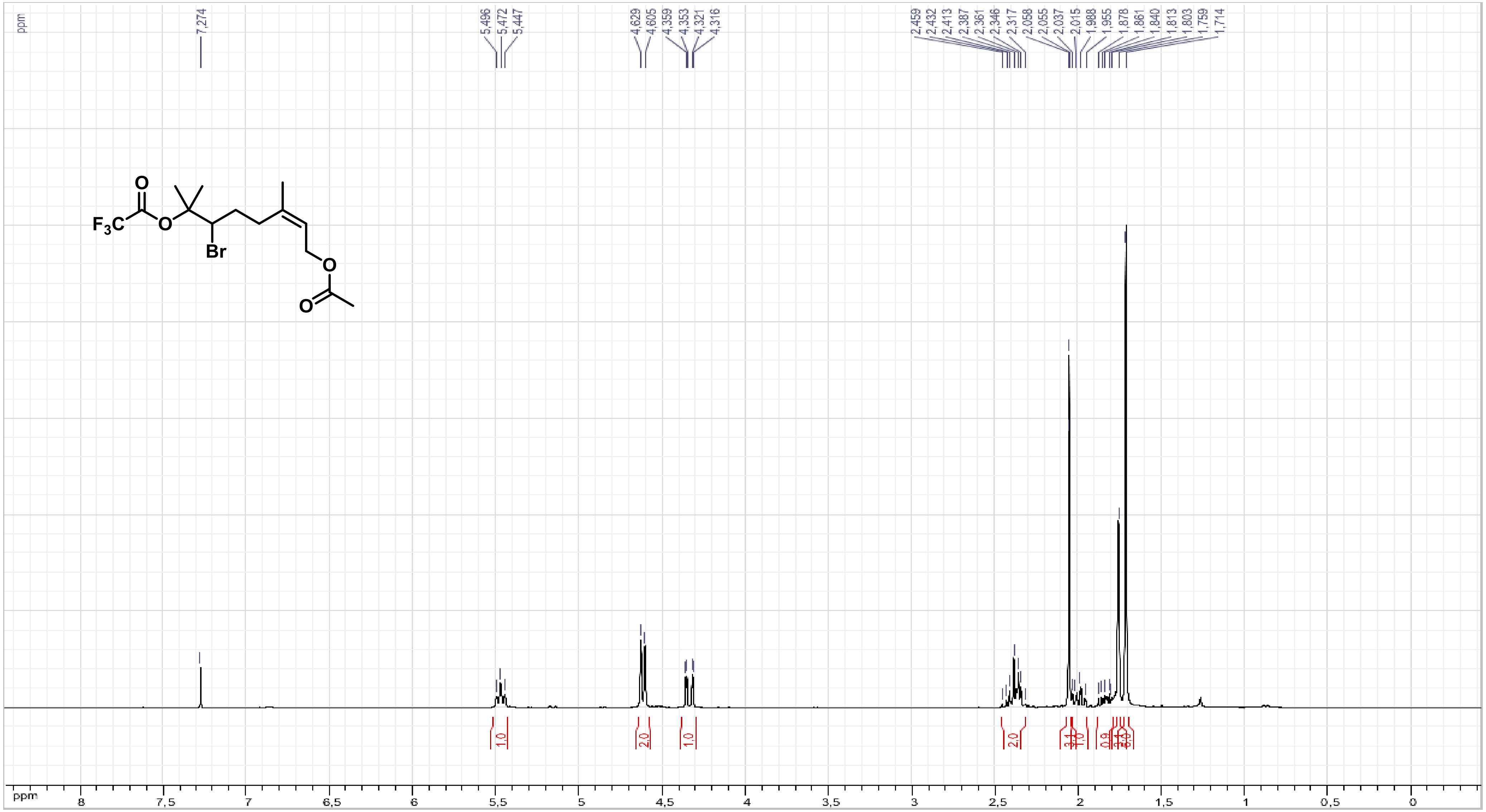

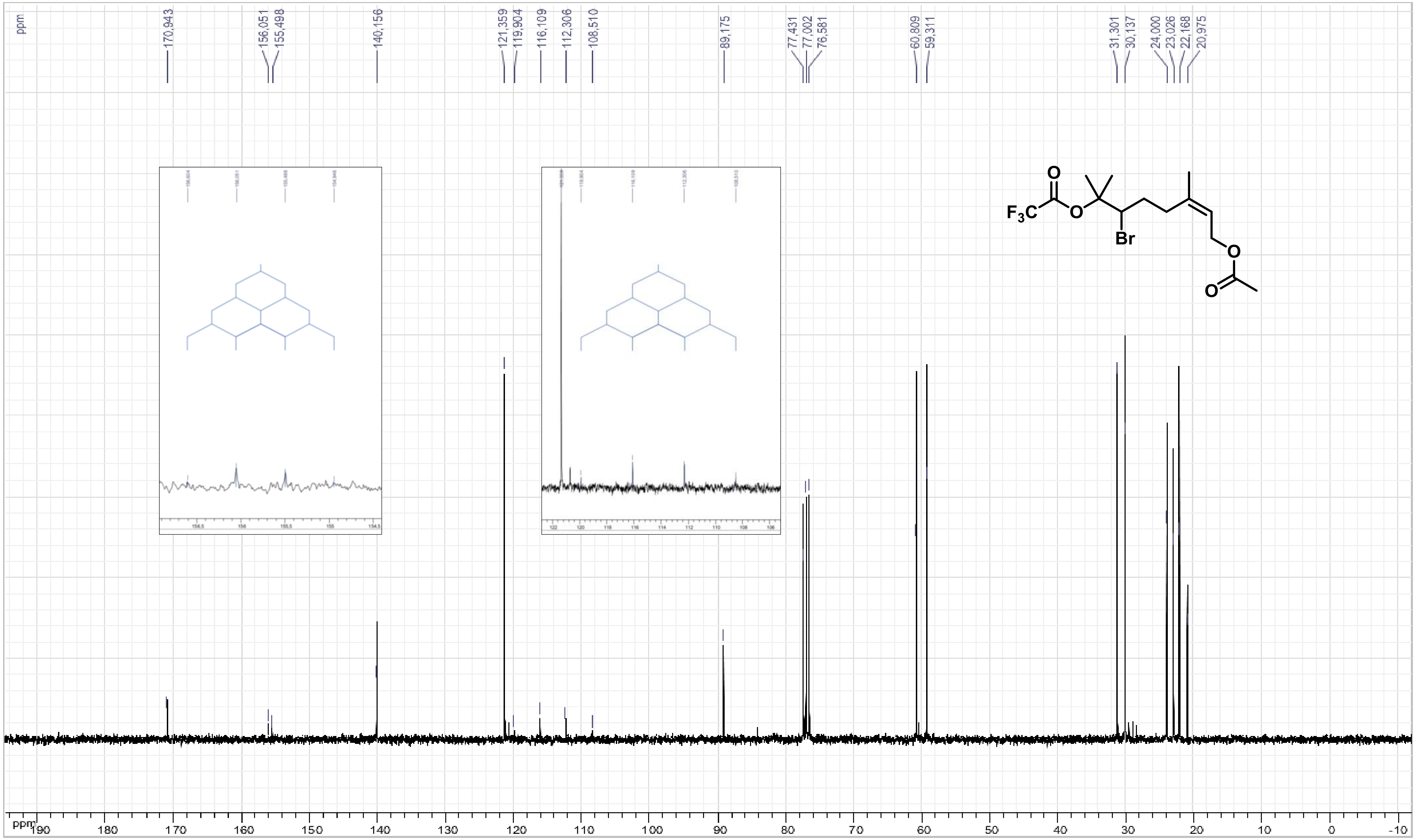
ppm
170,943
156,051
155,498
140,156
121,359
119,904
116,109
112,306
108,510
89,175
77,431
77,002
76,581
60,809
59,311
31,301
30,137
24,000
23,026
22,168
20,975
F3C
O
O
Br
O
O
ppm 190 180 170 160 150 140 130 120 110 100 90 80 70 60 50 40 30 20 10 0 -10

(E)-3-Bromo-8-hydroxy-2,6-dimethbyloct-6-en-2-yl 2,2,2-trifluoroacetate **(3c)**

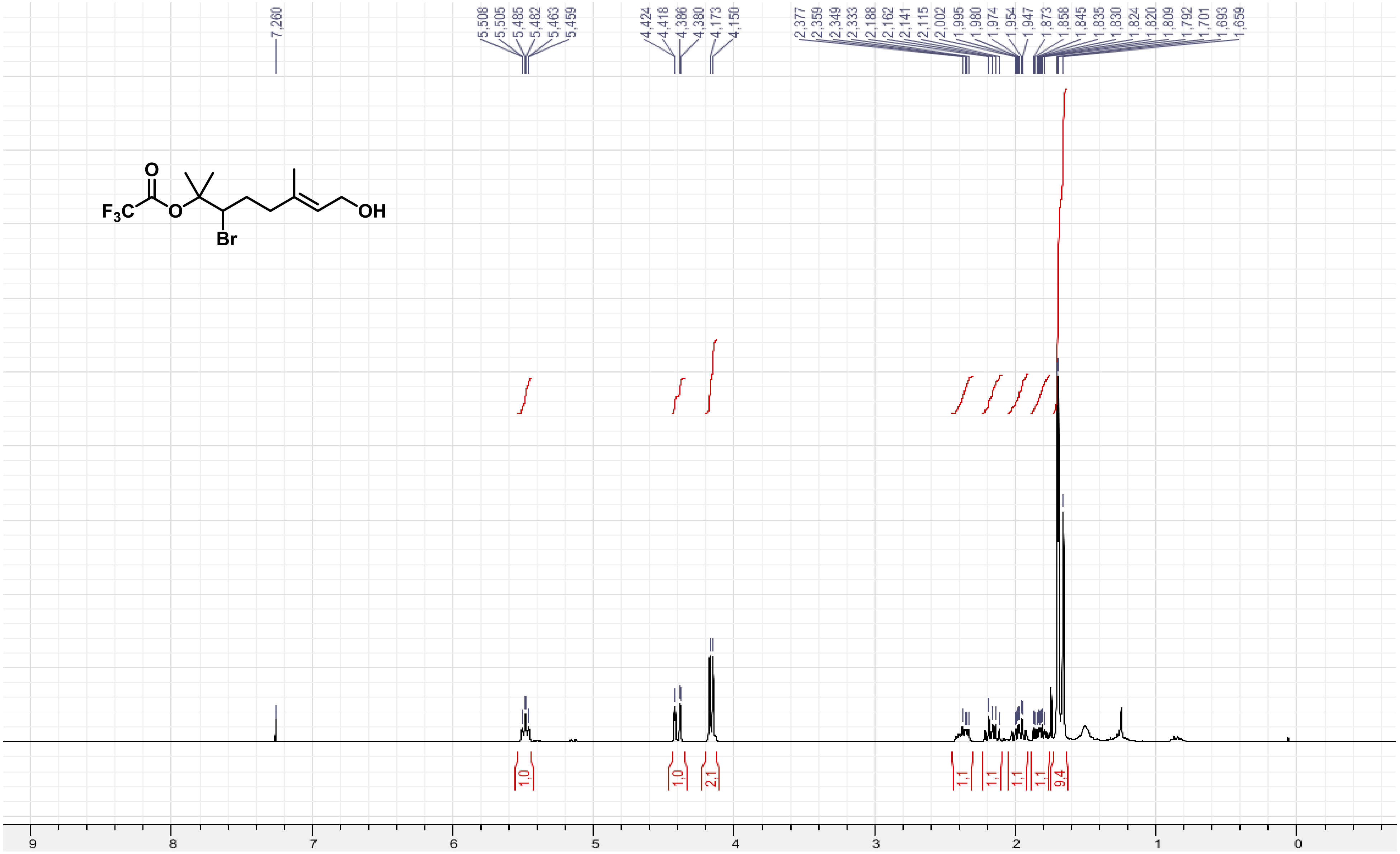

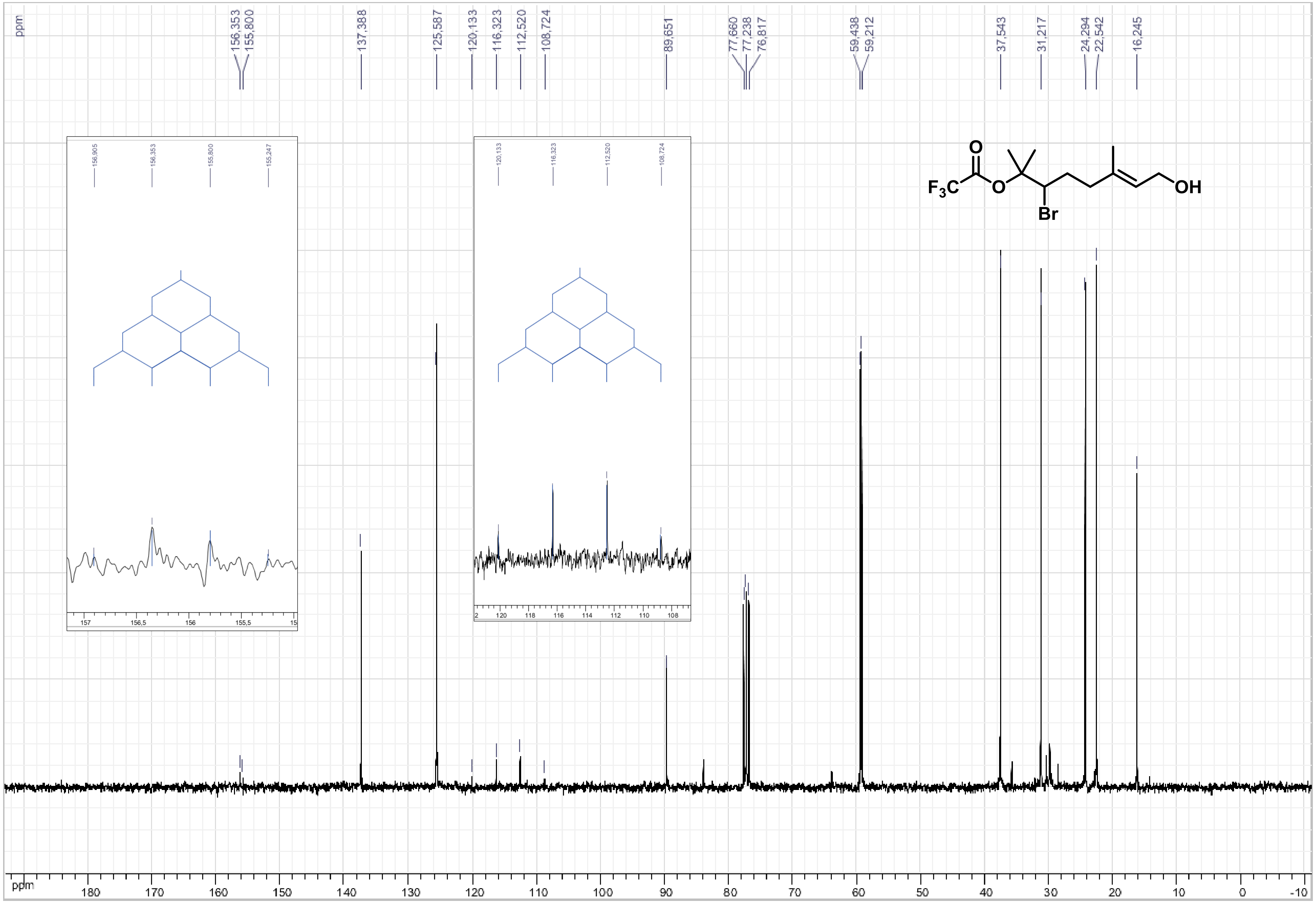

ppm
156,353
155,800
137,388
125,587
120,133
116,323
112,520
108,724
89,651
77,660
77,238
76,817
59,438
59,212
37,543
31,217
24,294
22,542
16,245
F3C
O
O
Br
OH

(E)-3-Bromo-2,6-dimethyl-8-((4-methylphenyl)sulfonamido)oct-6-en-2-yl 2,2,2-trifluoroacetate (3d)

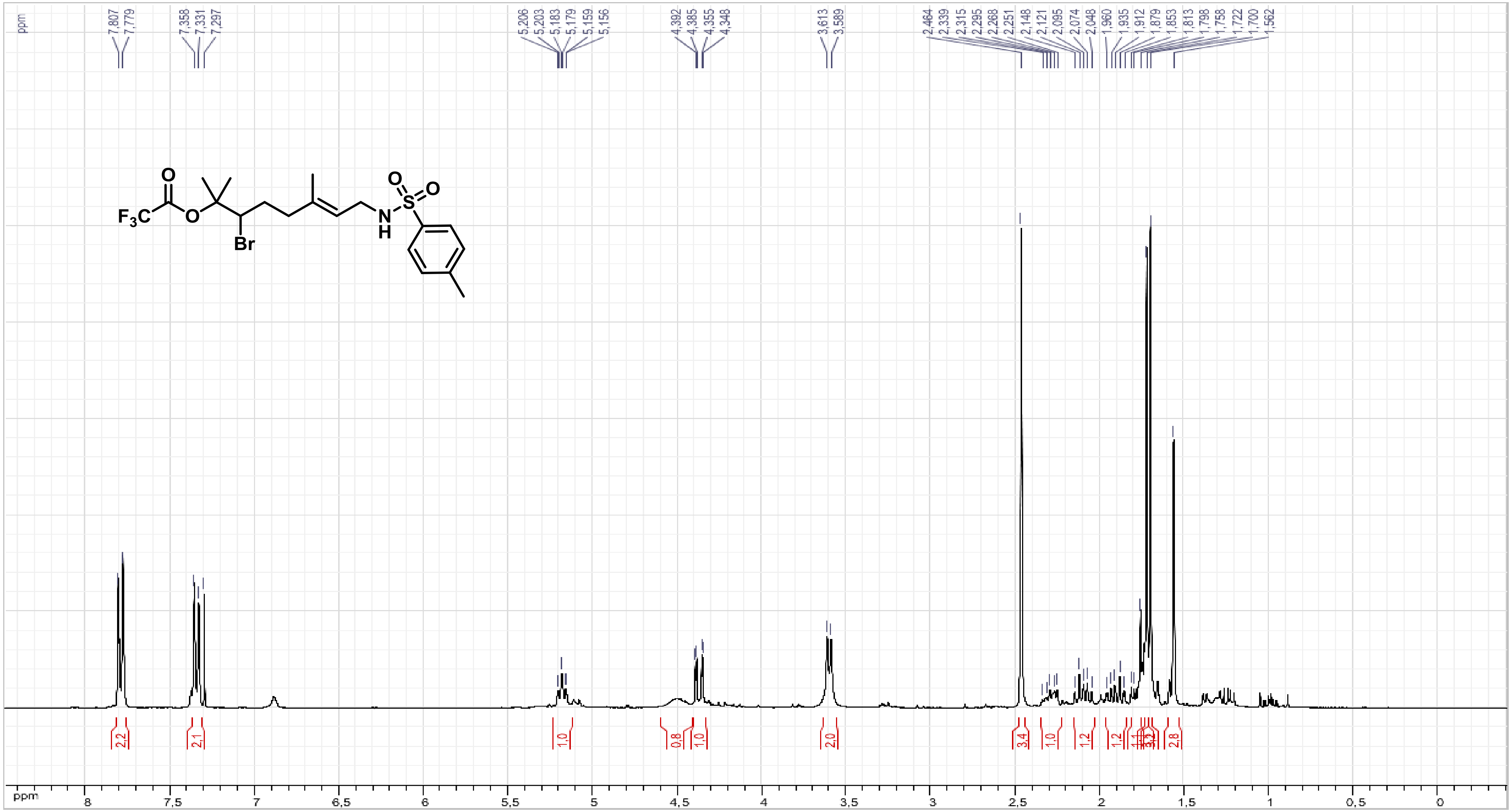

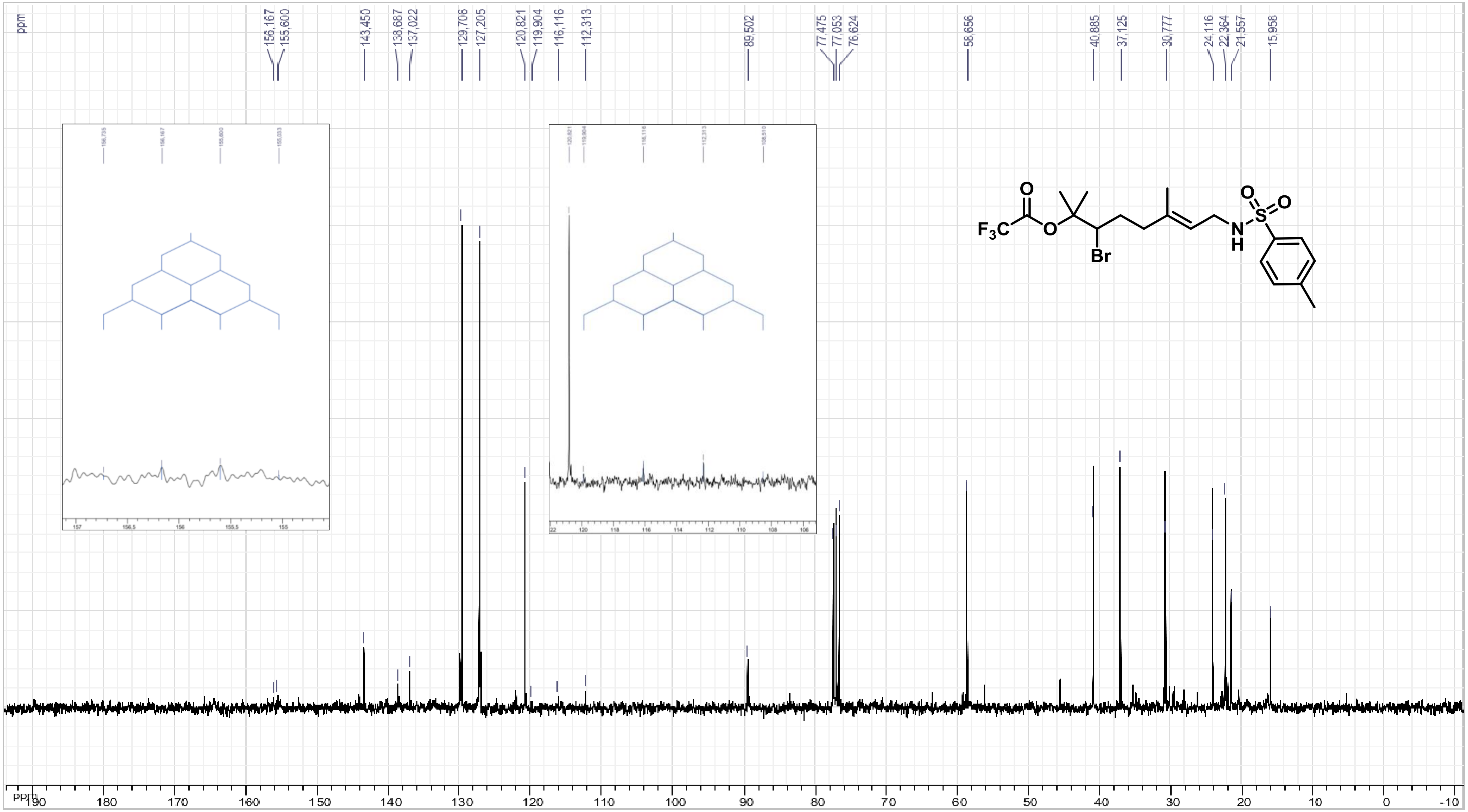
ppm
156,167
155,600
143,450
138,687
137,022
129,706
127,205
120,821
119,904
116,116
112,313
89,502
77,475
77,053
76,624
58,656
40,885
37,125
30,777
24,116
22,364
21,557
15,958
F_3C
O
O
Br
N
H
S
O
O
ppm
190
180
170
160
150
140
130
120
110
100
90
80
70
60
50
40
30
20
10
0
-10

3-Bromo-2-methyl-6-methyleneoct-7-en-2-yl 2,2,2-trifluoroacetate (3e)

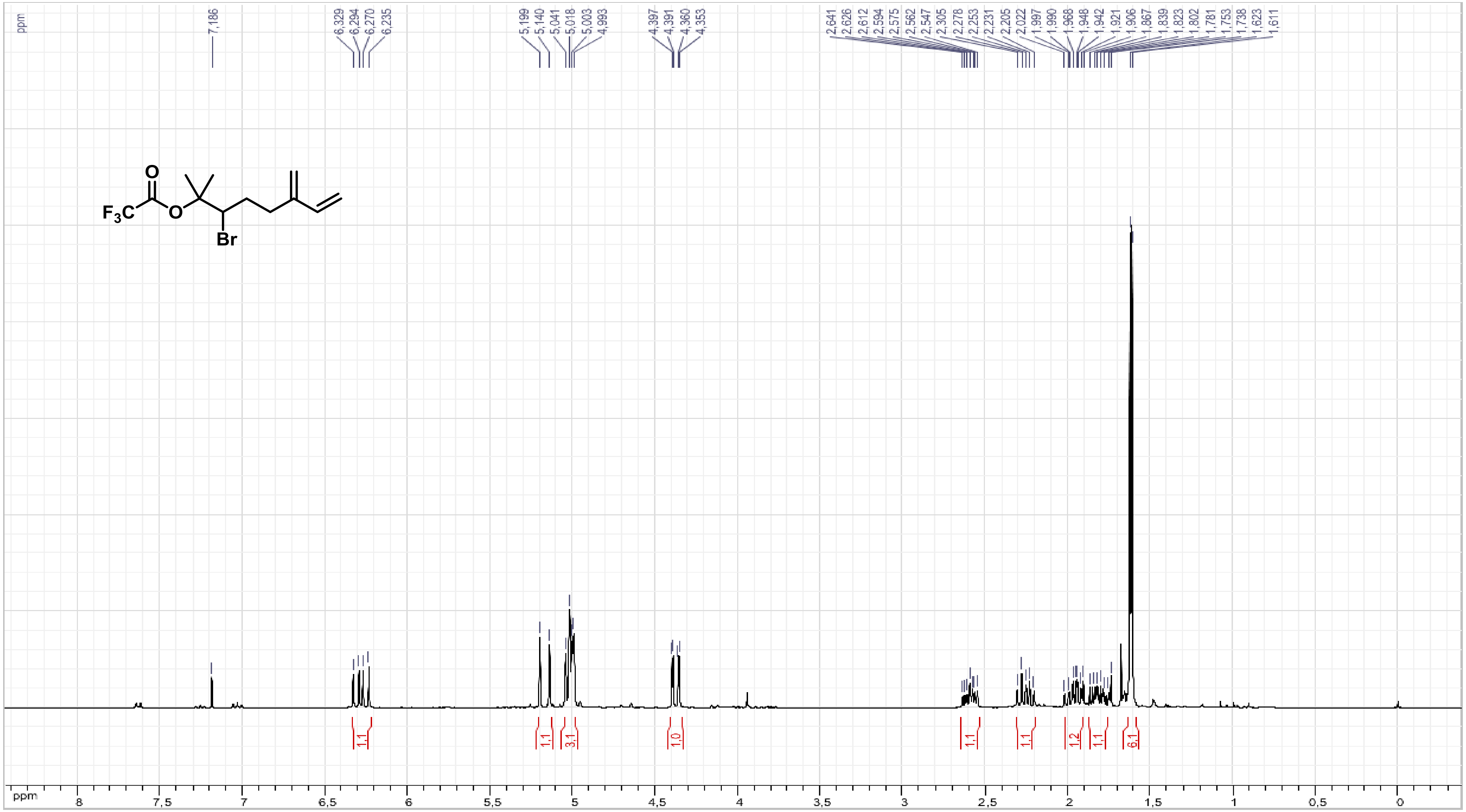

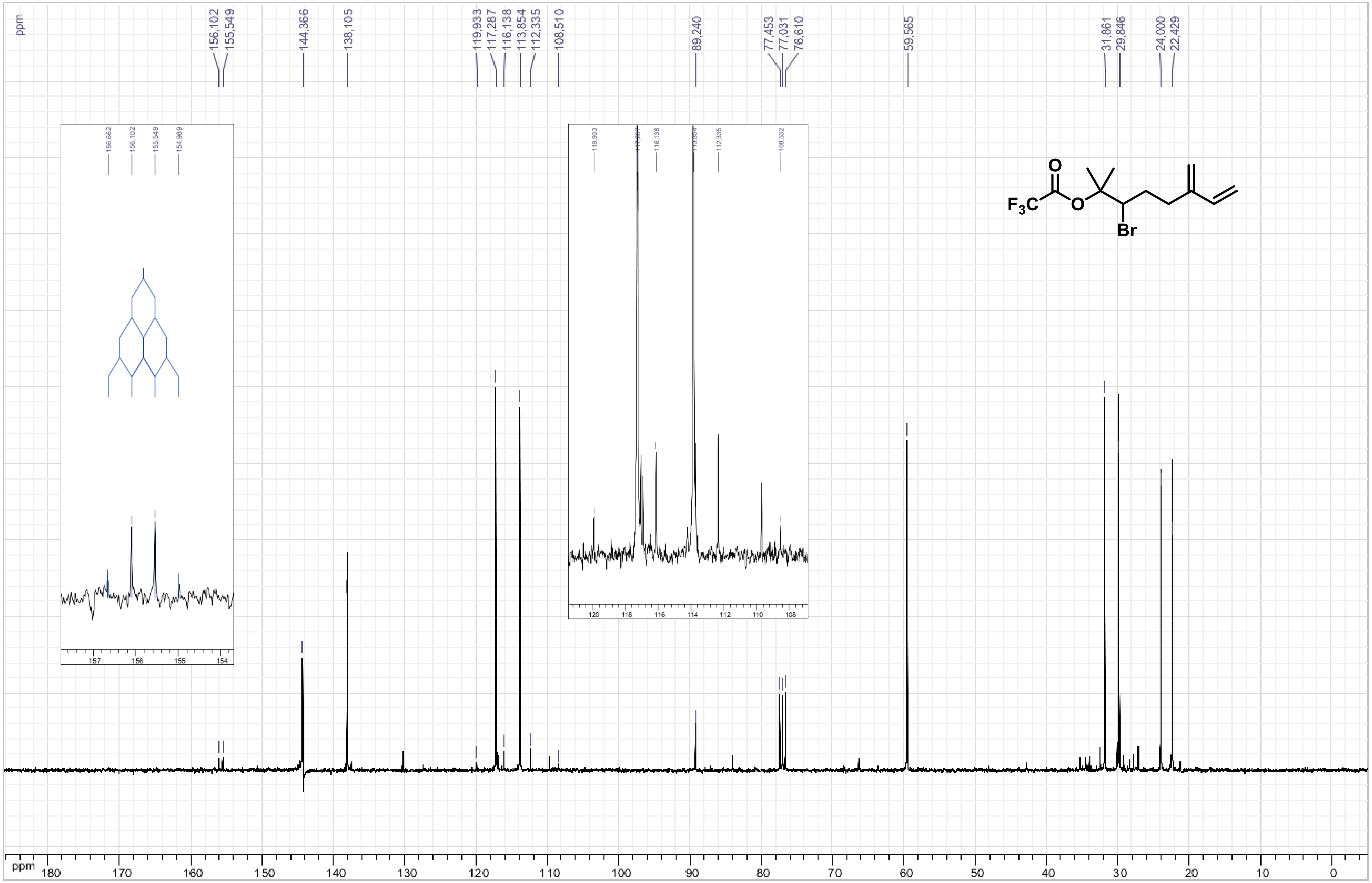
ppm
156,102
155,549
144,366
138,105
119,933
117,287
116,138
113,854
112,335
108,510
89,240
77,453
77,031
76,610
59,565
31,861
29,846
24,000
22,429
F3C
O
O
Br

***(E)-6-Bromo-7-hydroxy-3,7-dimethyloct-2-en-1-yl acetate* (4a)**

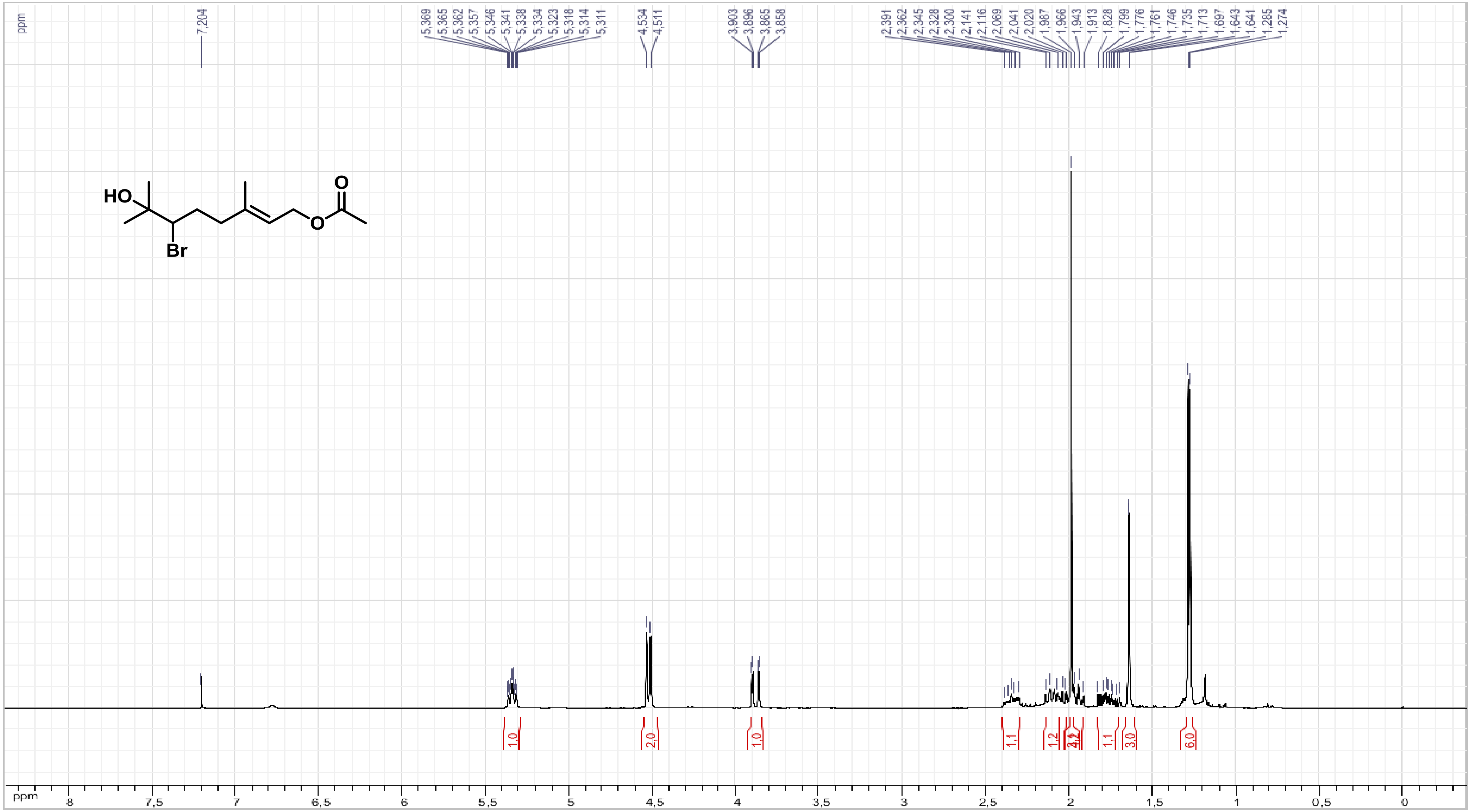

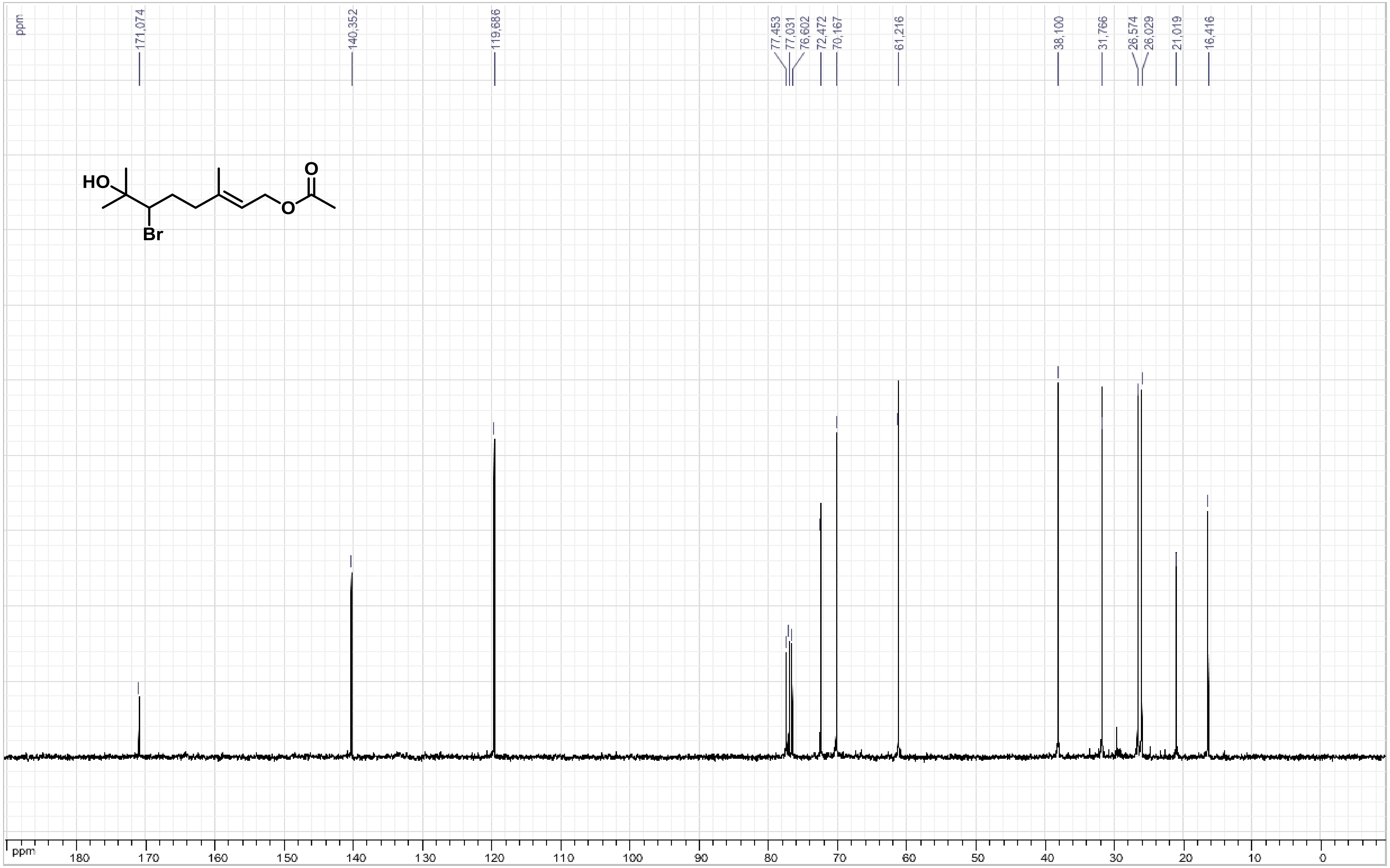
ppm
171.074
140.352
119.686
77.453
77.031
76.602
72.472
70.167
61.216
38.100
31.766
26.574
26.029
21.019
16.416
HO
Br
O
O
180
170
160
150
140
130
120
110
100
90
80
70
60
50
40
30
20
10
0

(Z)-6-Bromo-7-hydroxy-3,7-dimethyloct-2-en-1-yl acetate (4b)

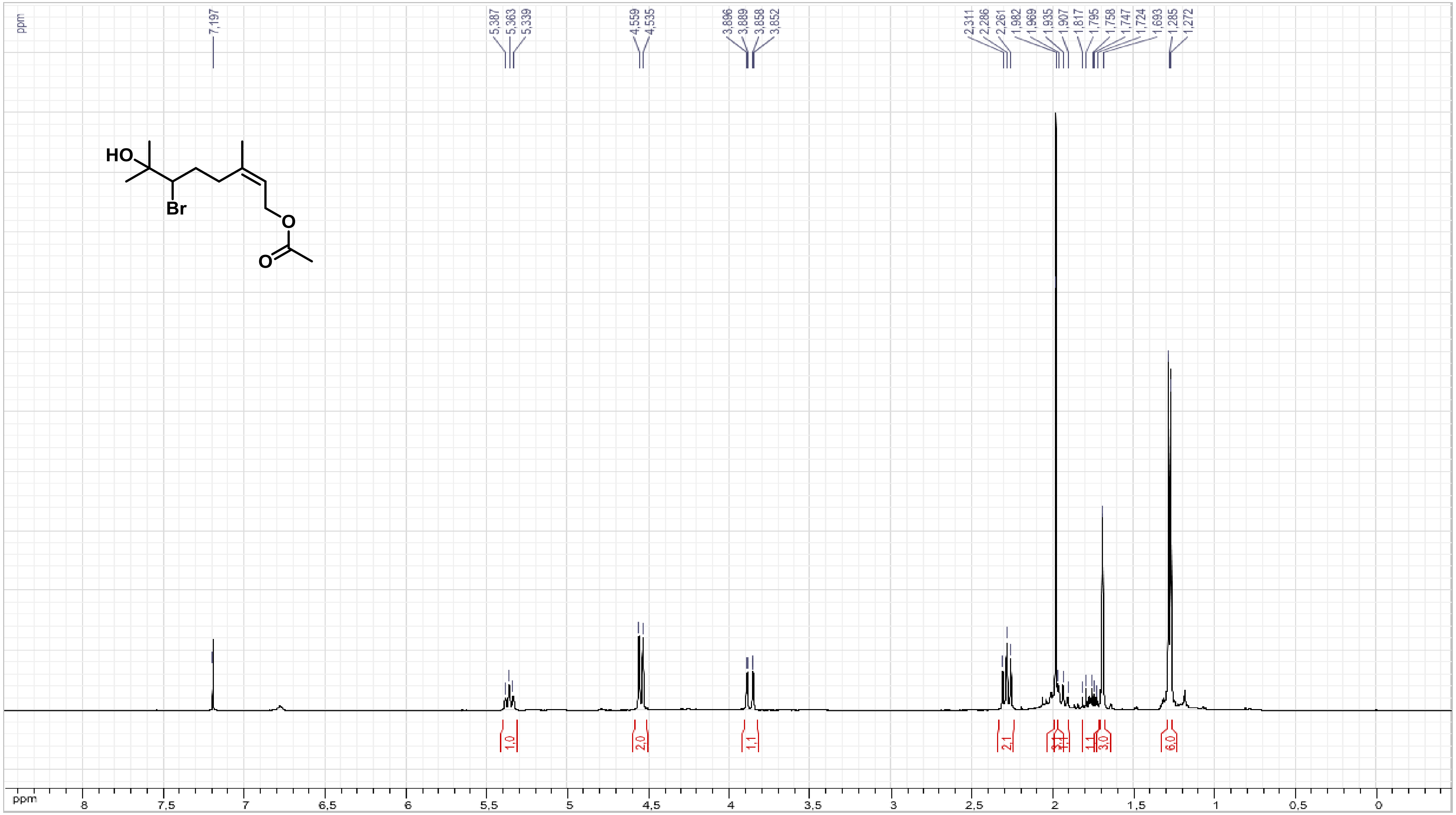

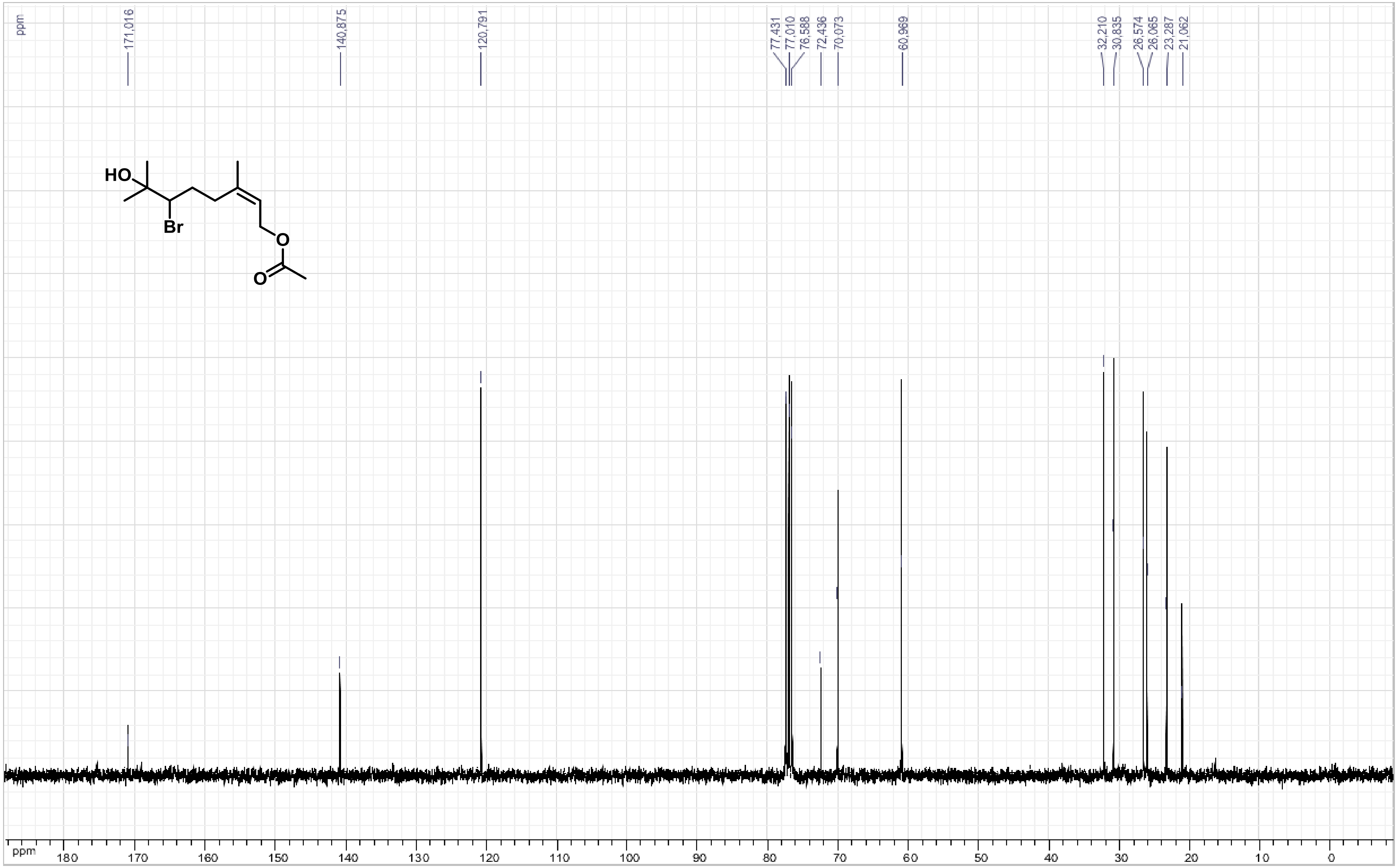
ppm
171.016
140.875
120.791
77.431
77.010
76.588
72.436
70.073
60.969
32.210
30.835
26.574
26.065
23.287
21.062
HO
Br
O
O
ppm 180 170 160 150 140 130 120 110 100 90 80 70 60 50 40 30 20 10 0

(E)-6-Bromo-3,7-dimethyloct-2-ene-1,7-diol (4c)

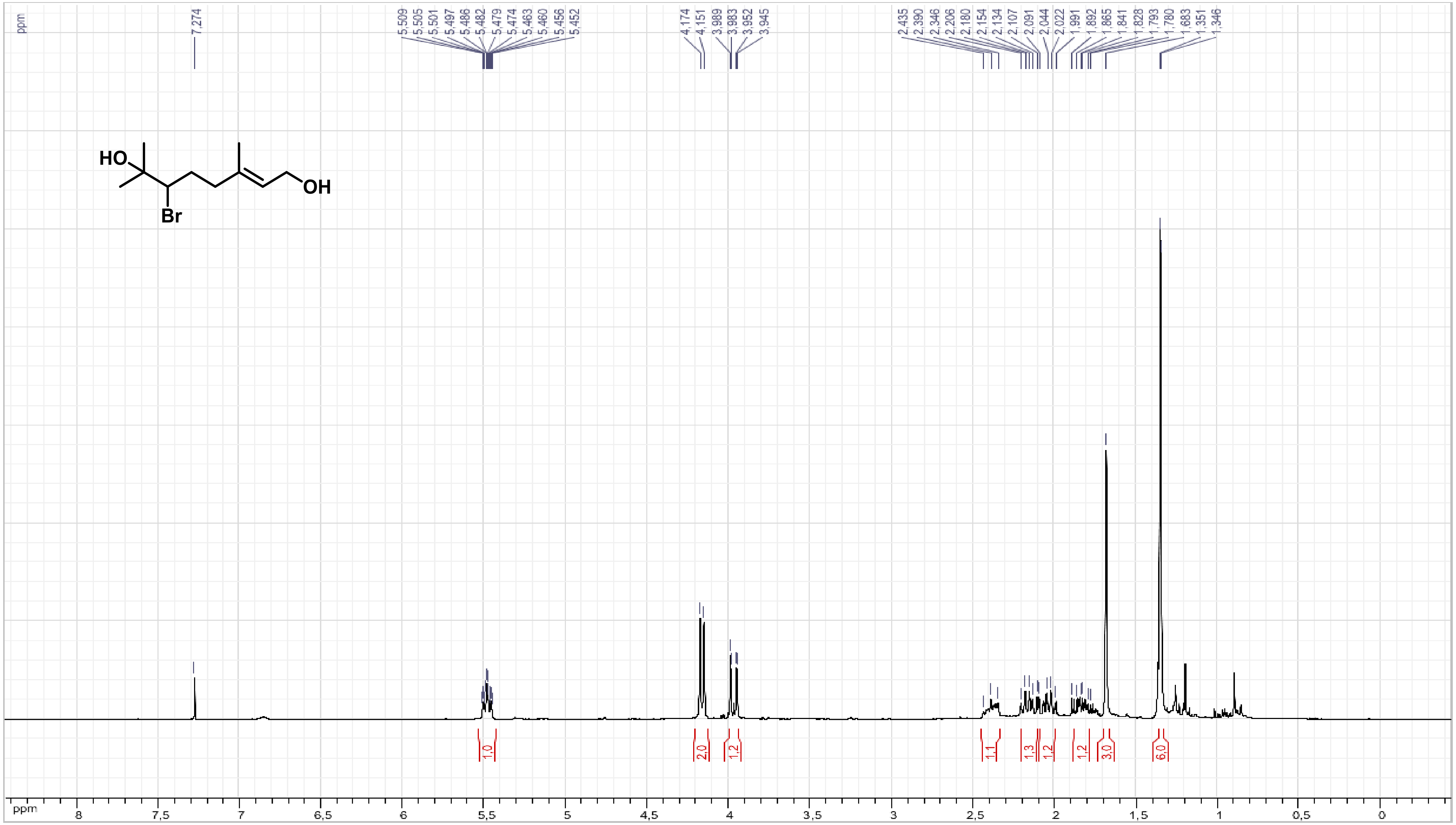

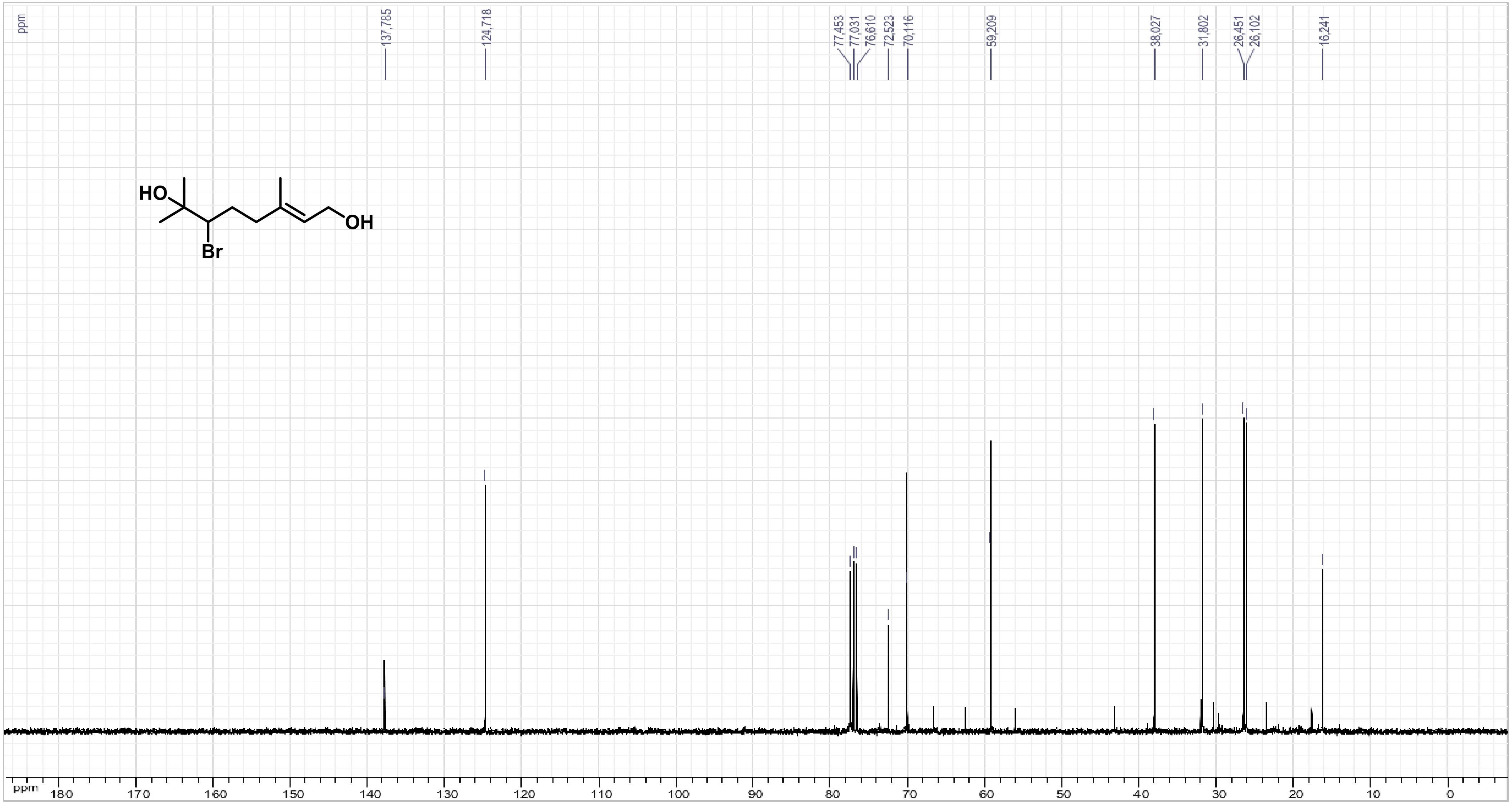

ppm
137.785
124.718
77.453
77.031
76.610
72.523
70.116
59.209
38.027
31.802
26.451
26.102
16.241
HO
OH
Br
ppm 180 170 160 150 140 130 120 110 100 90 80 70 60 50 40 30 20 10 0

(E)-N-(6-Bromo-7-hydroxy-3,7-dimethyloct-2-en-1-yl)-4-methylbenzenesulfonamide (4d)

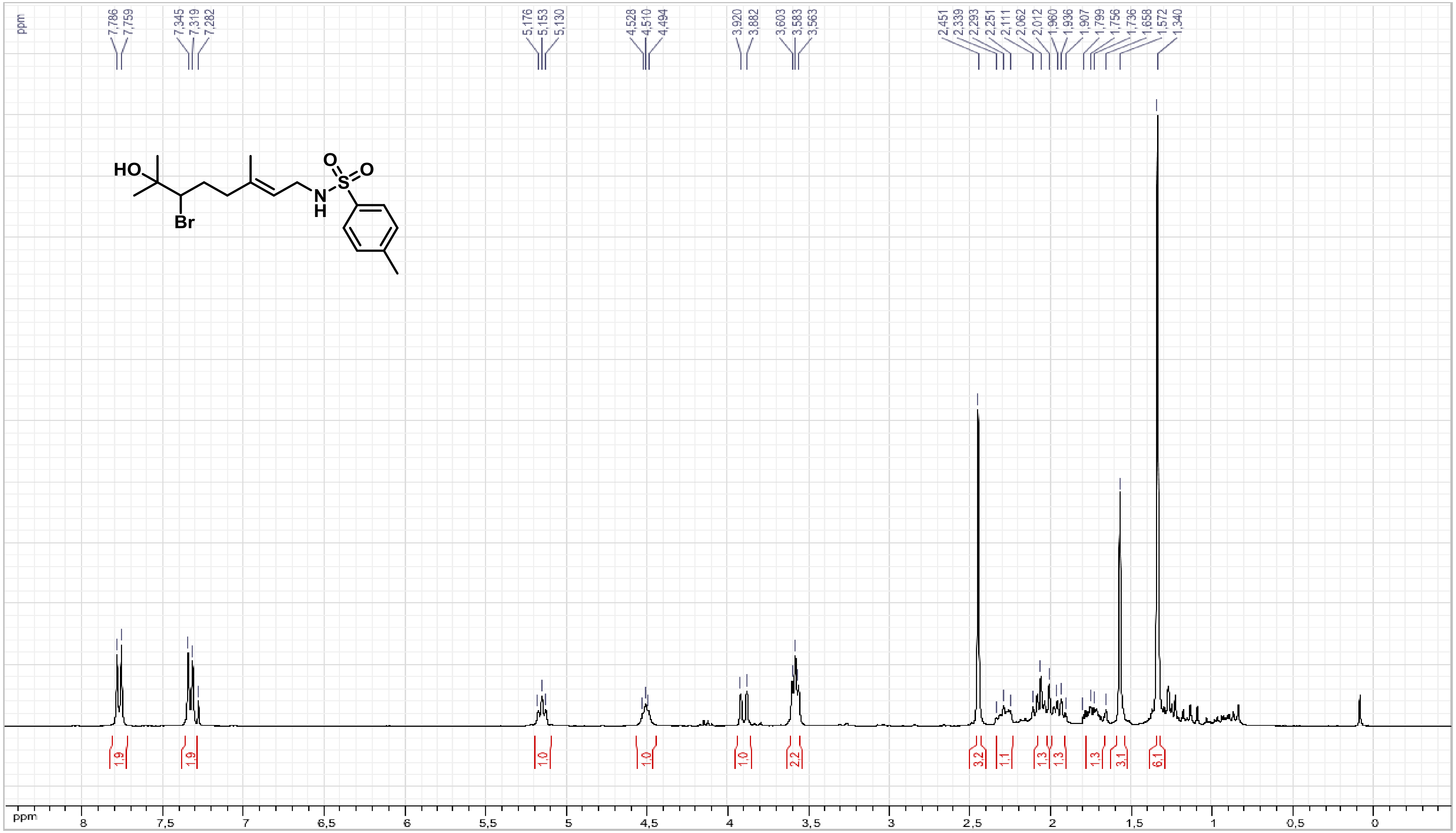

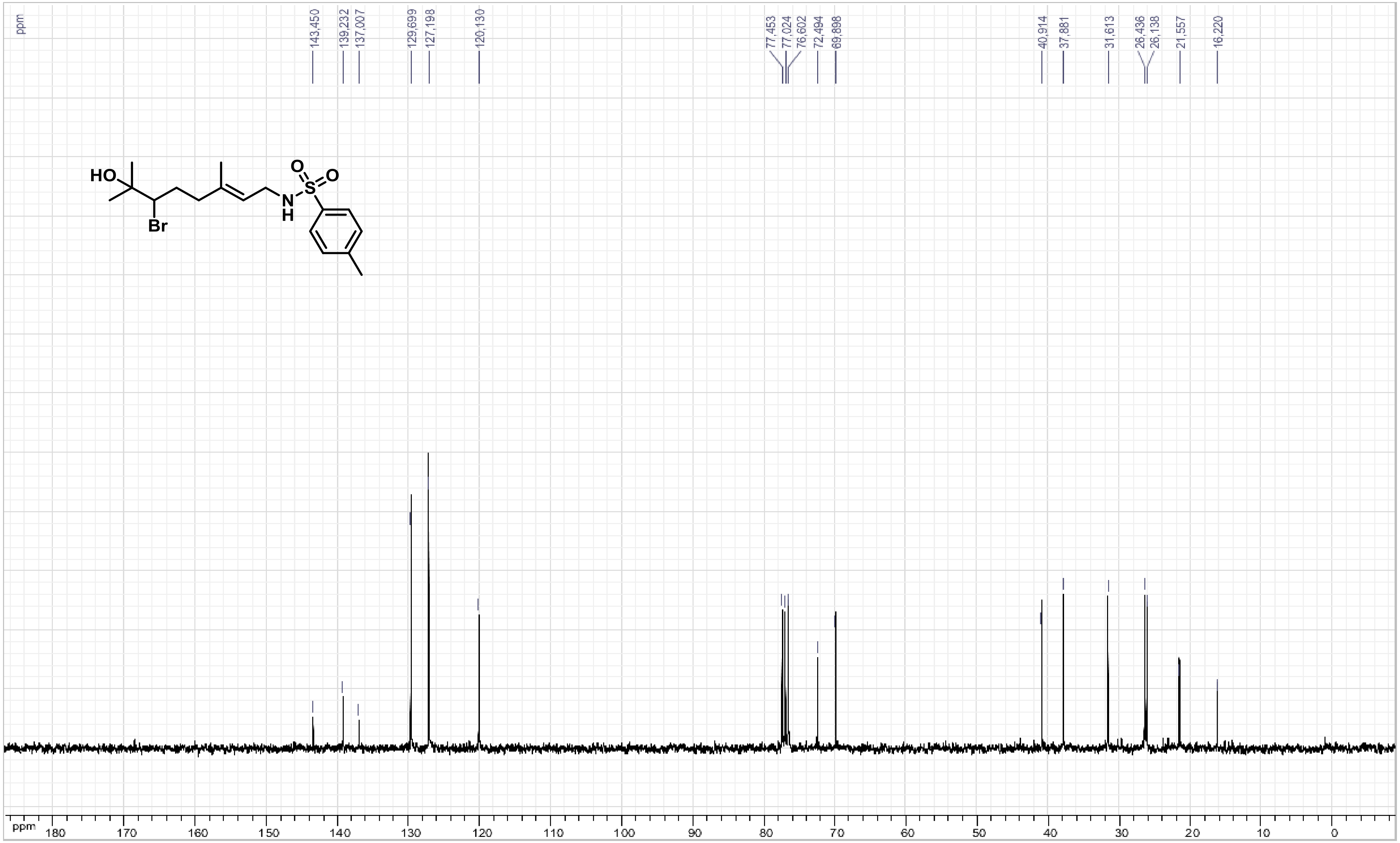

ppm
143,450
139,232
137,007
129,699
127,198
120,130
77,453
77,024
76,602
72,494
69,898
40,914
37,881
31,613
26,436
26,138
21,557
16,220
HO
Br
N
H
S
O
O
ppm
180
170
160
150
140
130
120
110
100
90
80
70
60
50
40
30
20
10
0

3-Bromo-2-methyl-6-methyleneoct-7-en-2-ol (4e)

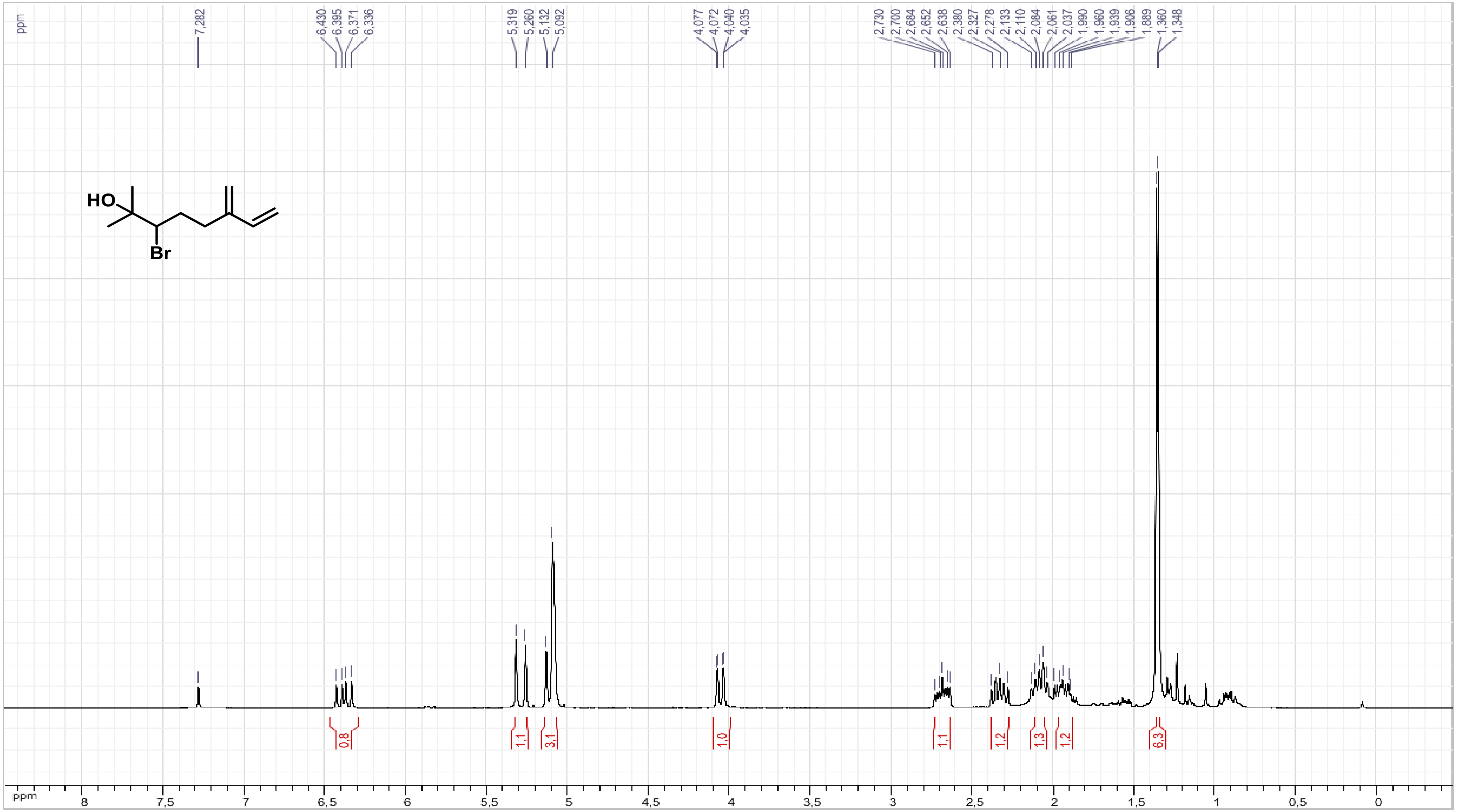

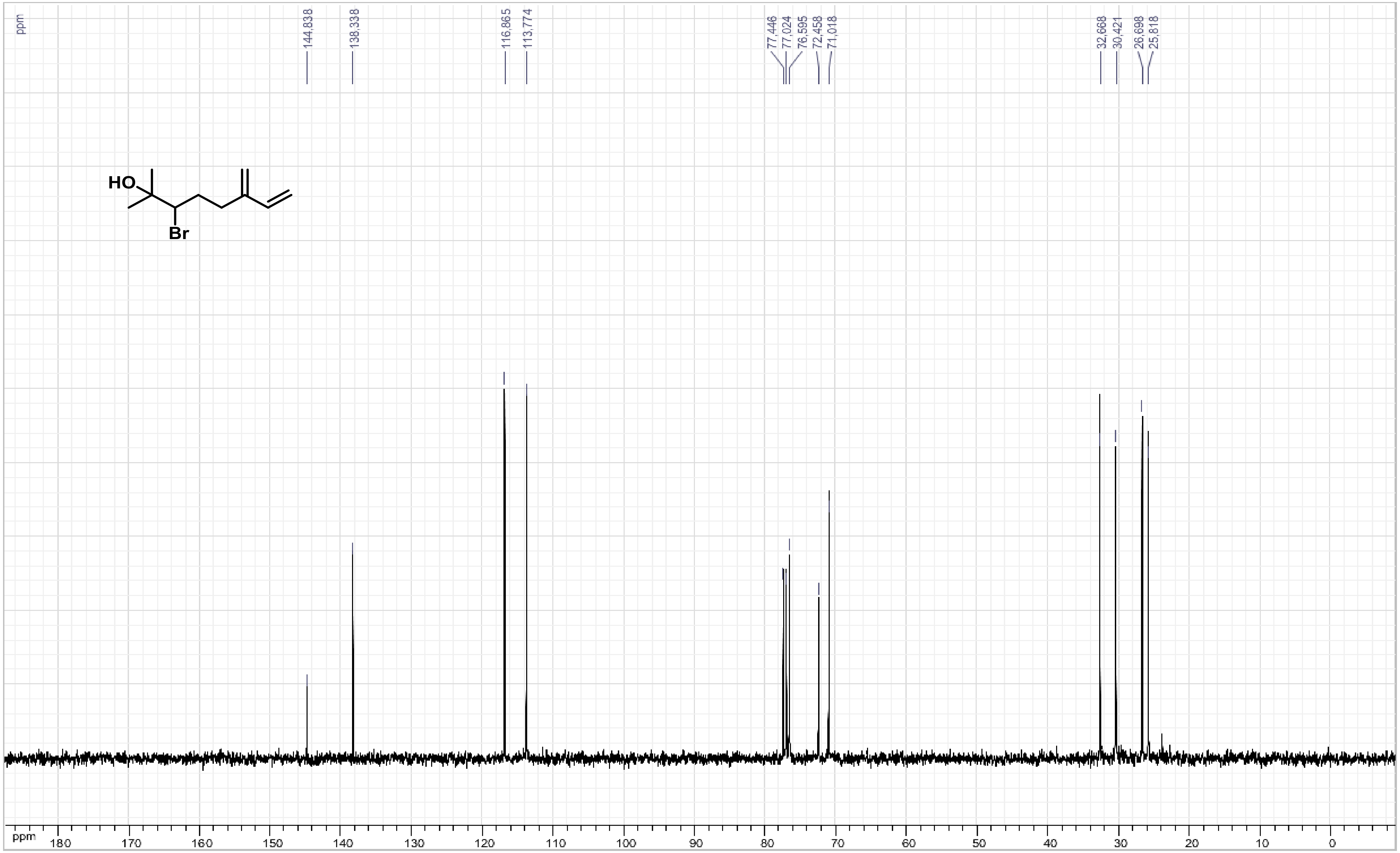

ppm
144.838
138.338
116.865
113.774
77.446
77.024
76.595
72.458
71.018
32.668
30.421
26.698
25.818
HO
Br
ppm 180 170 160 150 140 130 120 110 100 90 80 70 60 50 40 30 20 10 0

***(E)*-8-Acetoxy-3-iodo-2,6-dimethyloct-6-en-2-yl 2,2,2-trifluoroacetate** (5a)*

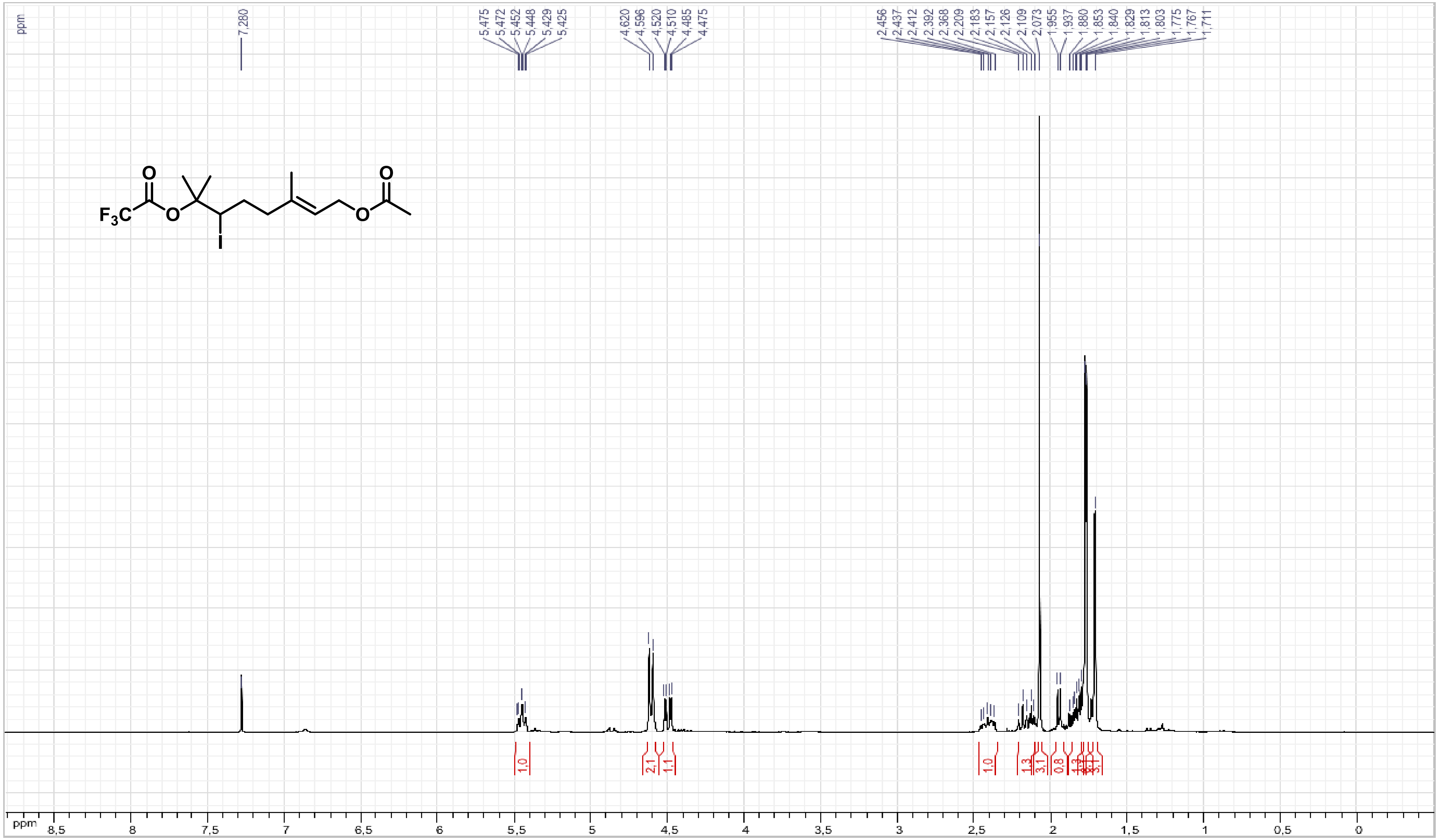

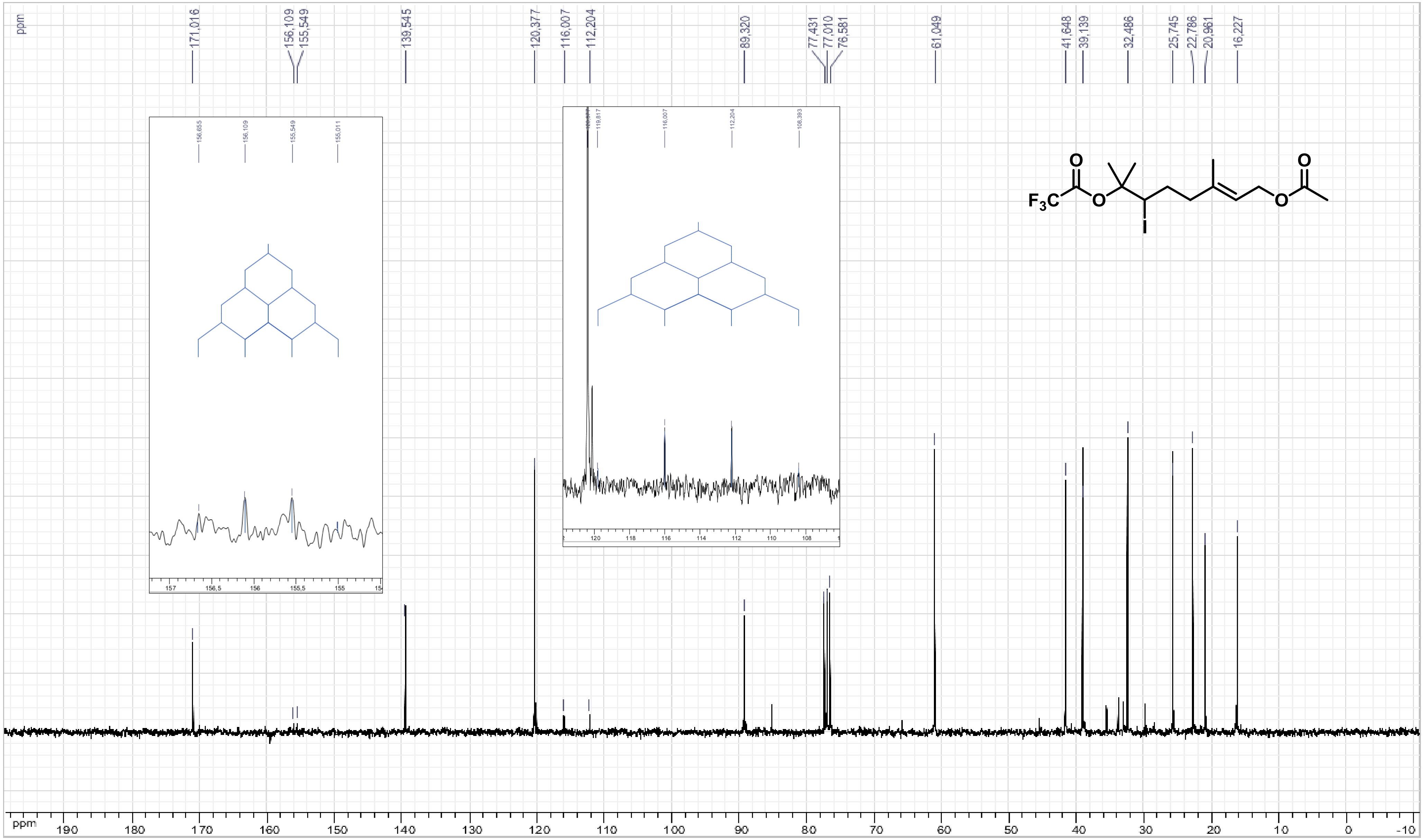
ppm
171,016
156,109
155,549
139,545
120,377
116,007
112,204
89,320
77,431
77,010
76,581
61,049
41,648
39,139
32,486
25,745
22,786
20,961
16,227
156,655
156,109
155,549
155,011
119,817
116,007
112,204
108,393
F_3C
O
O
O
O
I
ppm 190 180 170 160 150 140 130 120 110 100 90 80 70 60 50 40 30 20 10 0 -10

(Z)-8-Acetoxy-3-iodo-2,6-dimethyloct-6-en-2-yl 2,2,2-trifluoroacetate (5b)

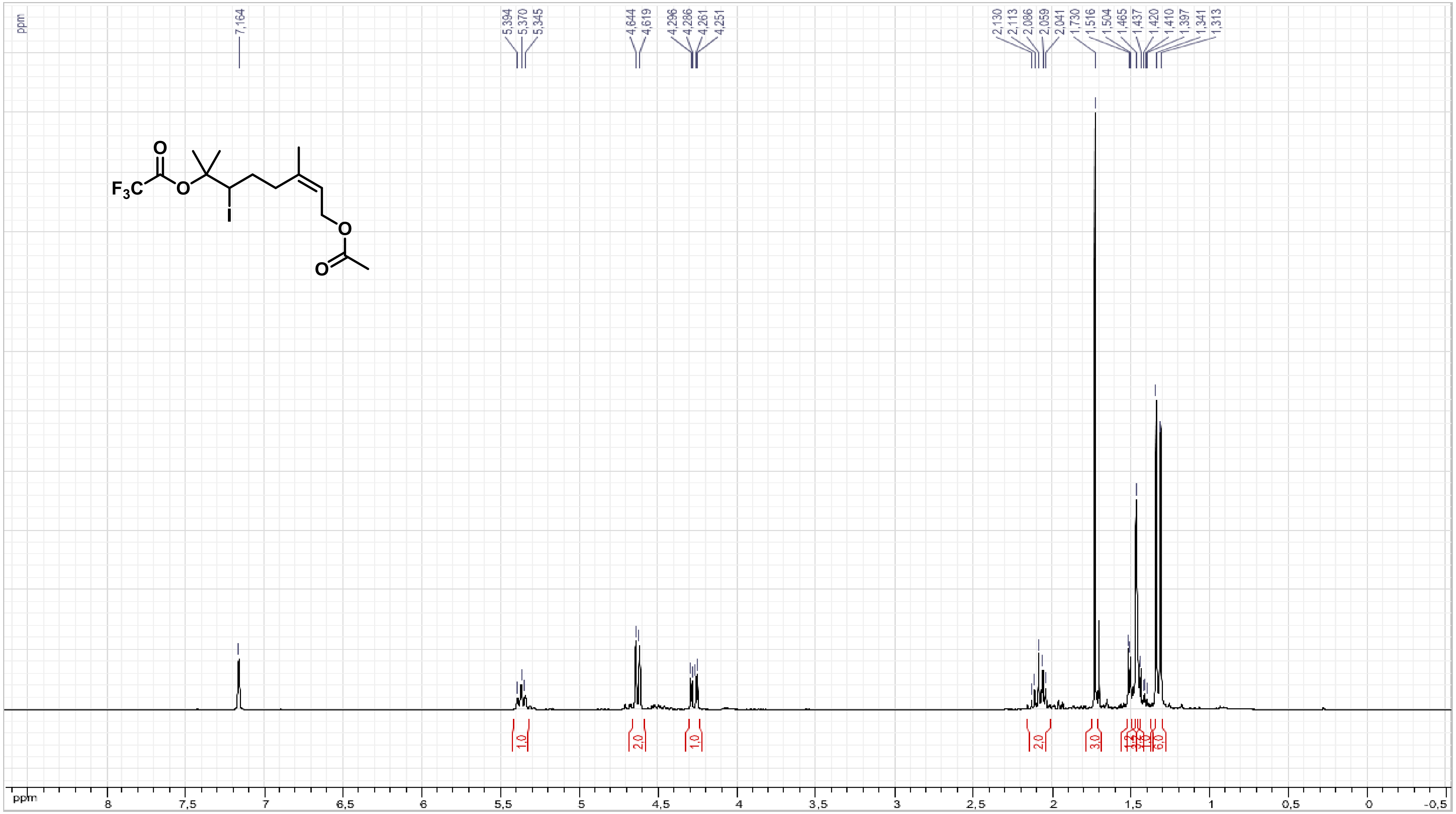

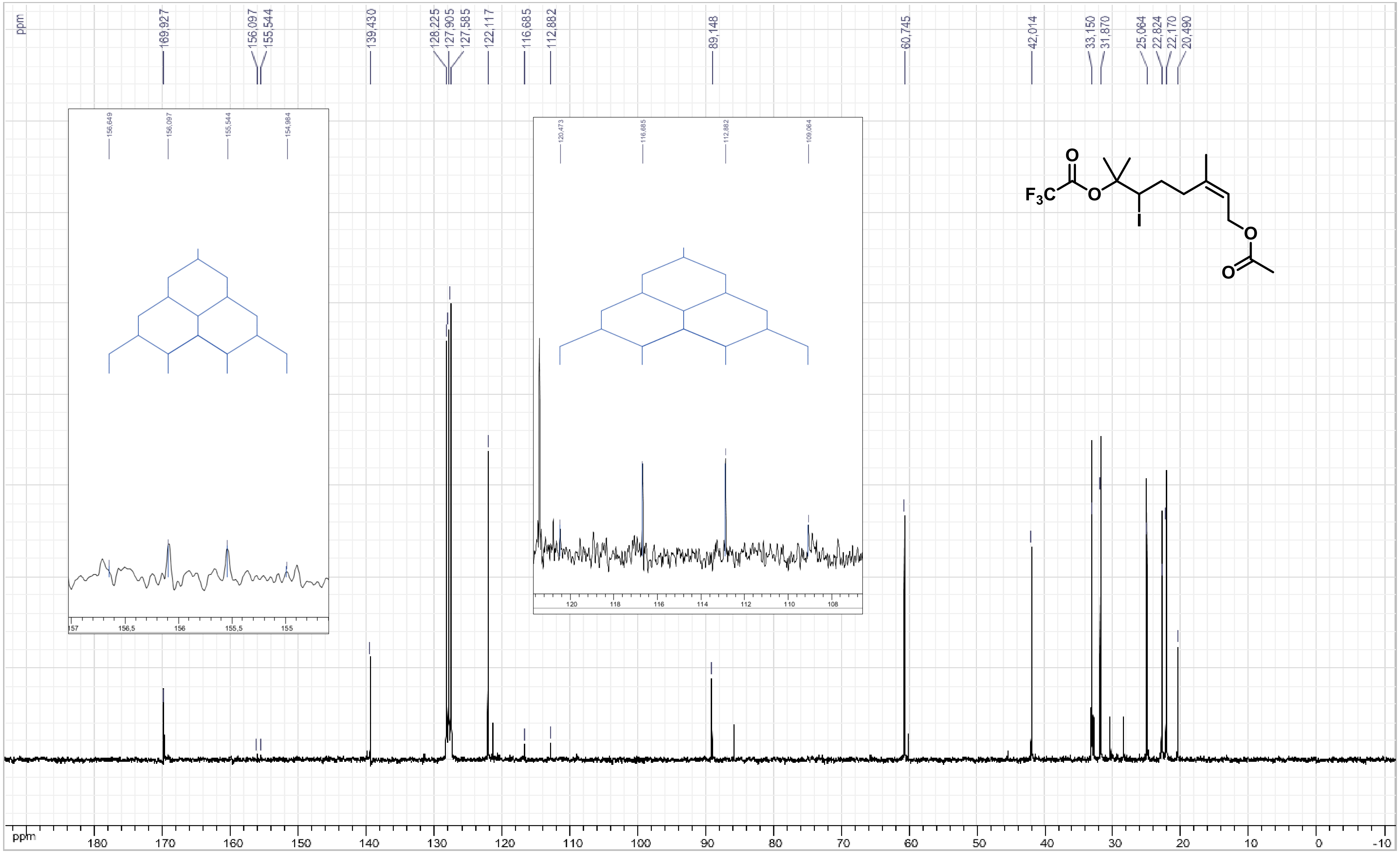
ppm
169.927
156.097
155.544
139.430
128.225
127.905
127.585
122.117
116.685
112.882
89.148
60.745
42.014
33.150
31.870
25.064
22.824
22.170
20.490
156.649
156.097
155.544
154.984
120.473
116.685
112.882
109.064
F3C
O
I
180 170 160 150 140 130 120 110 100 90 80 70 60 50 40 30 20 10 0 -10

(E)-8-Hydroxy-3-iodo-2,6-dimethyloct-6-en-2-yl 2,2,2-trifluoroacetate (5c)

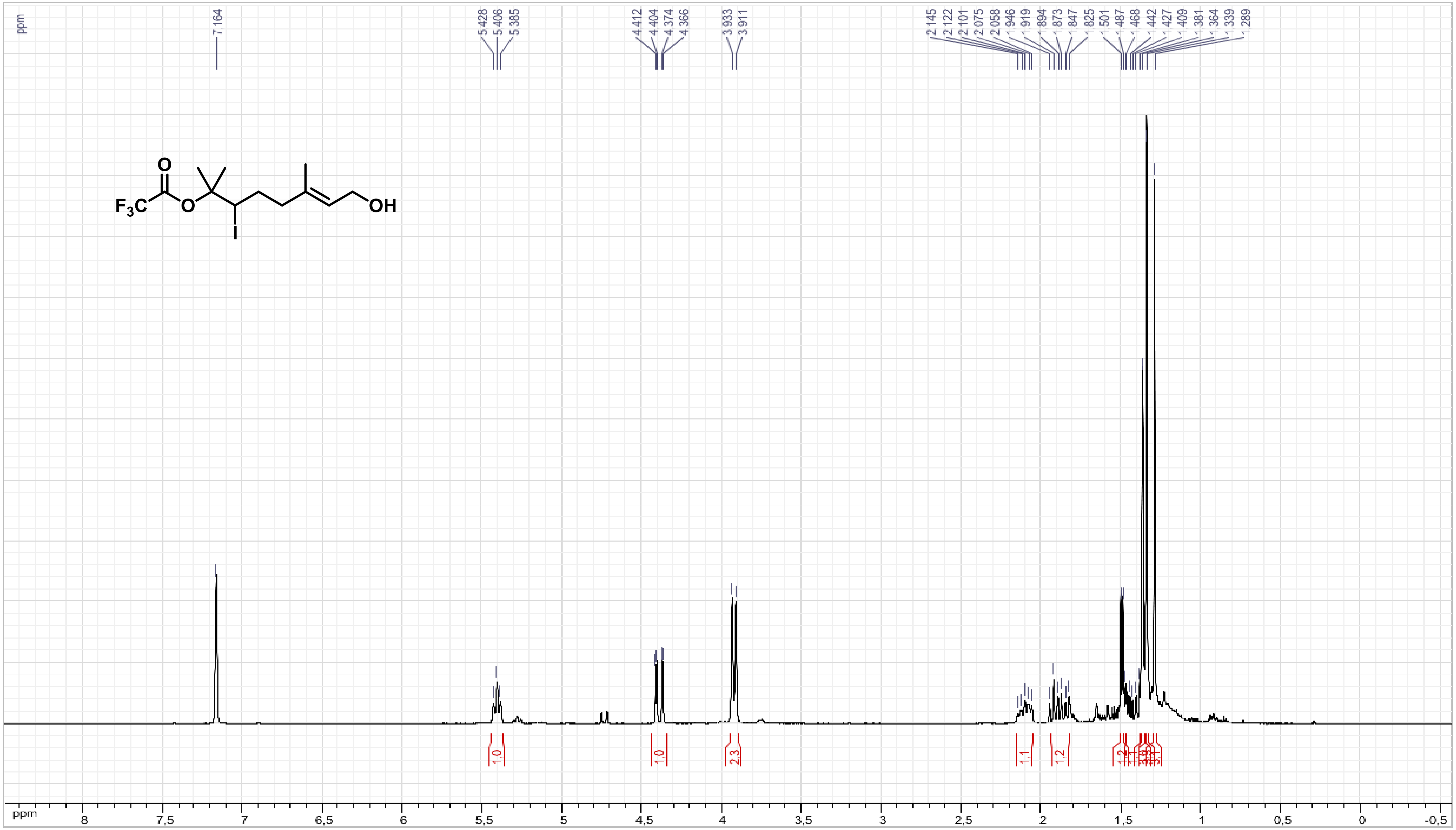

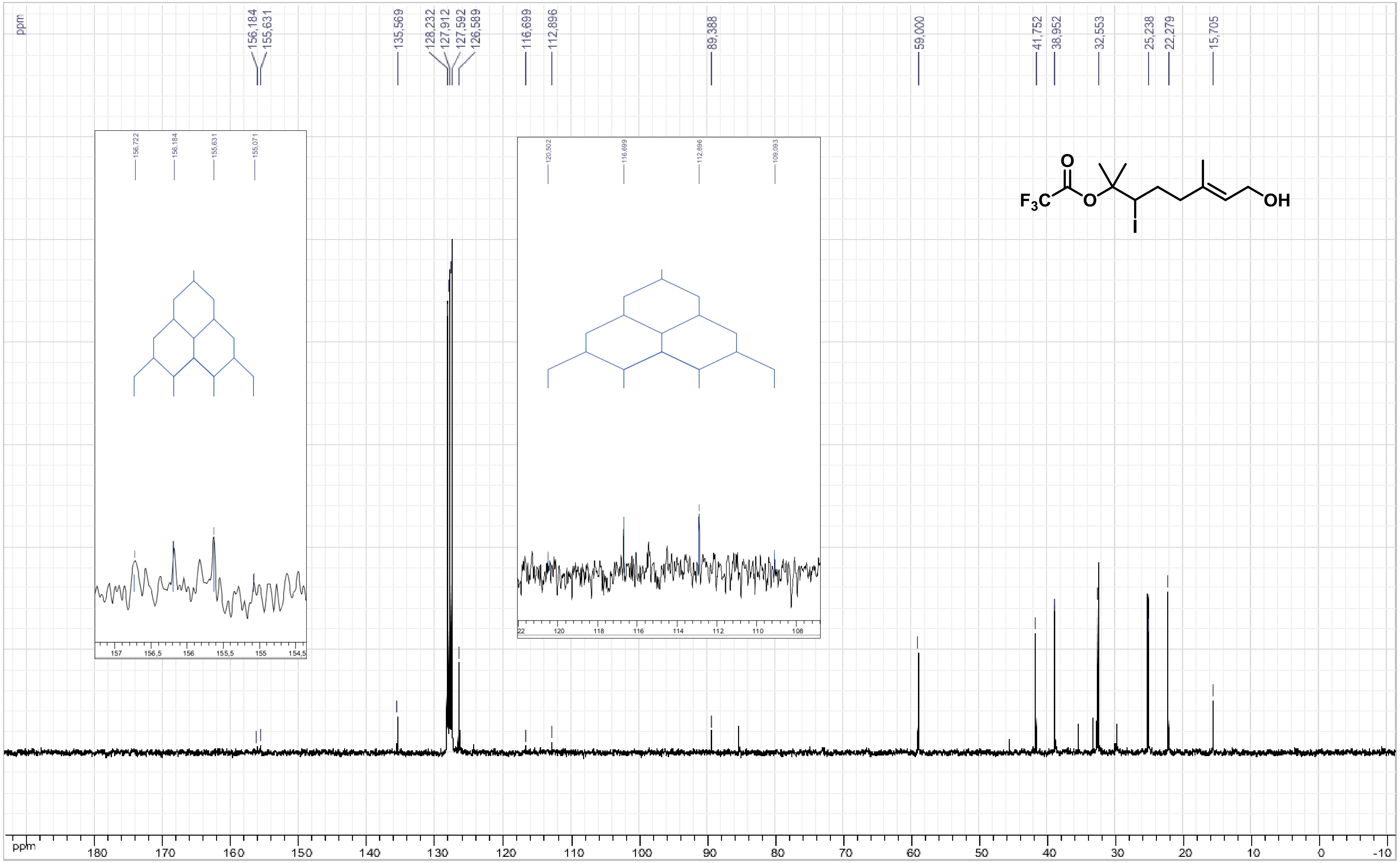

ppm
156,184
155,631
135,569
128,232
127,912
127,592
126,589
116,699
112,896
89,388
59,000
41,752
38,952
32,553
25,238
22,279
15,705
156,722
156,184
155,631
155,071
120,502
116,699
112,896
109,093
O
F_3C
O
OH
I
180
170
160
150
140
130
120
110
100
90
80
70
60
50
40
30
20
10
0
-10

(E)-3-Iodo-2,6-dimethyl-8-((4-methylphenyl)sulfonamido)oct-6-en-2-yl 2,2,2-trifluoroacetate (5d)

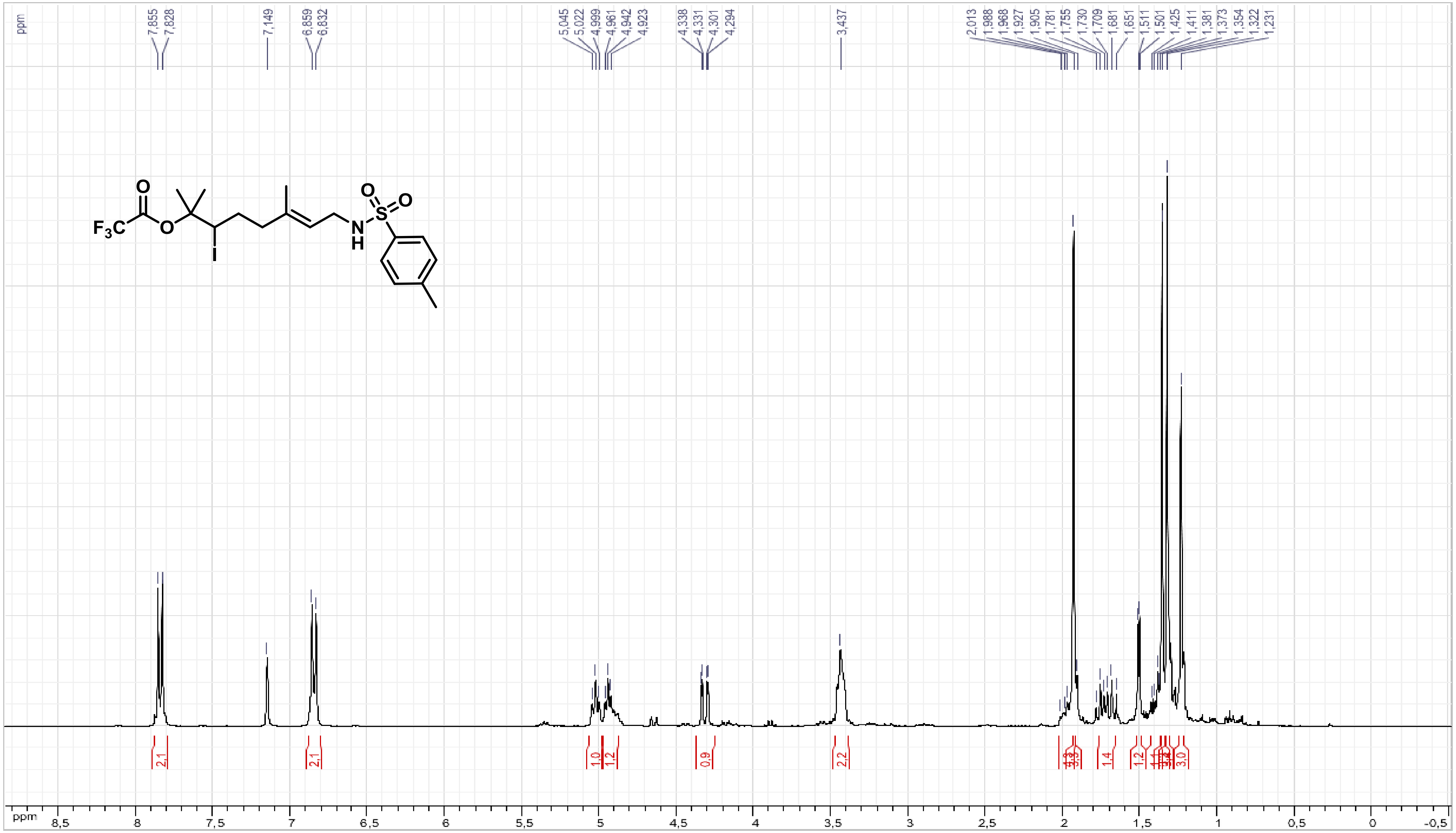

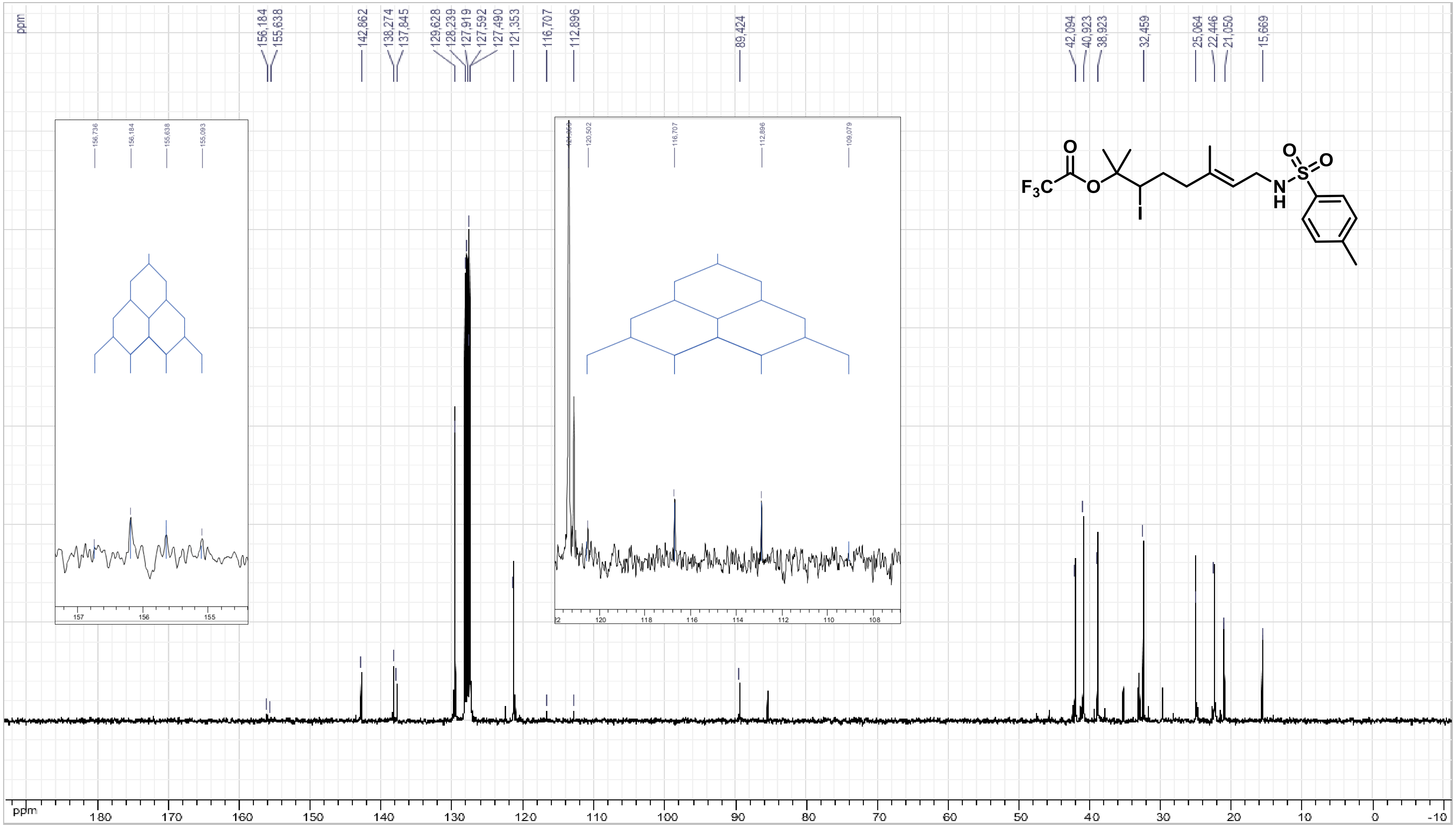
ppm
156.184
155.638
142.862
138.274
137.845
129.628
128.239
127.919
127.592
127.490
121.353
116.707
112.896
89.424
42.094
40.923
38.923
32.459
25.064
22.446
21.050
15.669
156.736
156.184
155.638
155.093
157
156
155
120.502
116.707
112.896
109.079
120
118
116
114
112
110
108
F_3C
O
O
I
N
H
S
O
O
ppm
180
170
160
150
140
130
120
110
100
90
80
70
60
50
40
30
20
10
0
-10

3-Iodo-2-methyl-6-methyleneoct-7-en-2-yl 2,2,2-trifluoroacetate (5e)

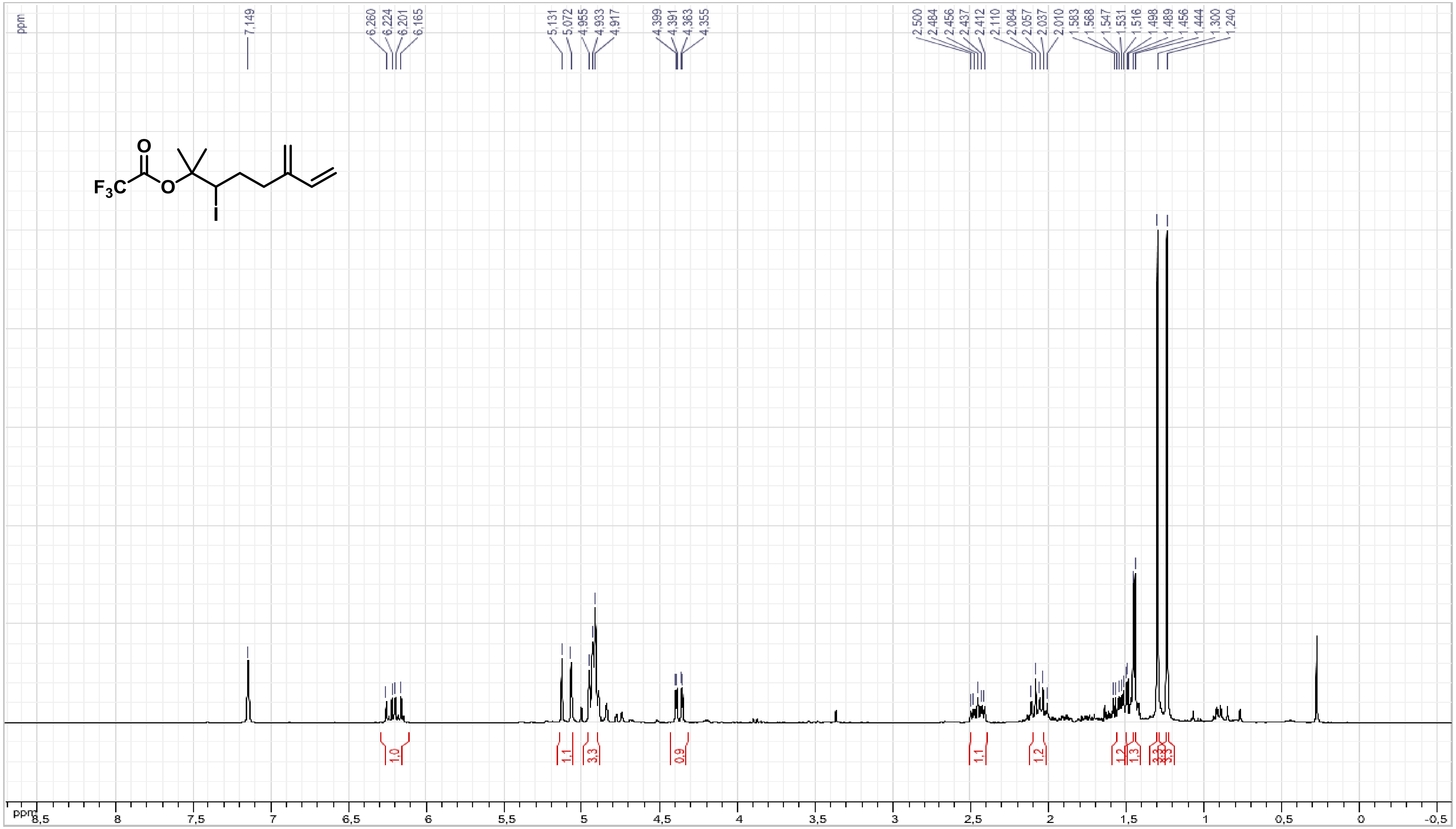

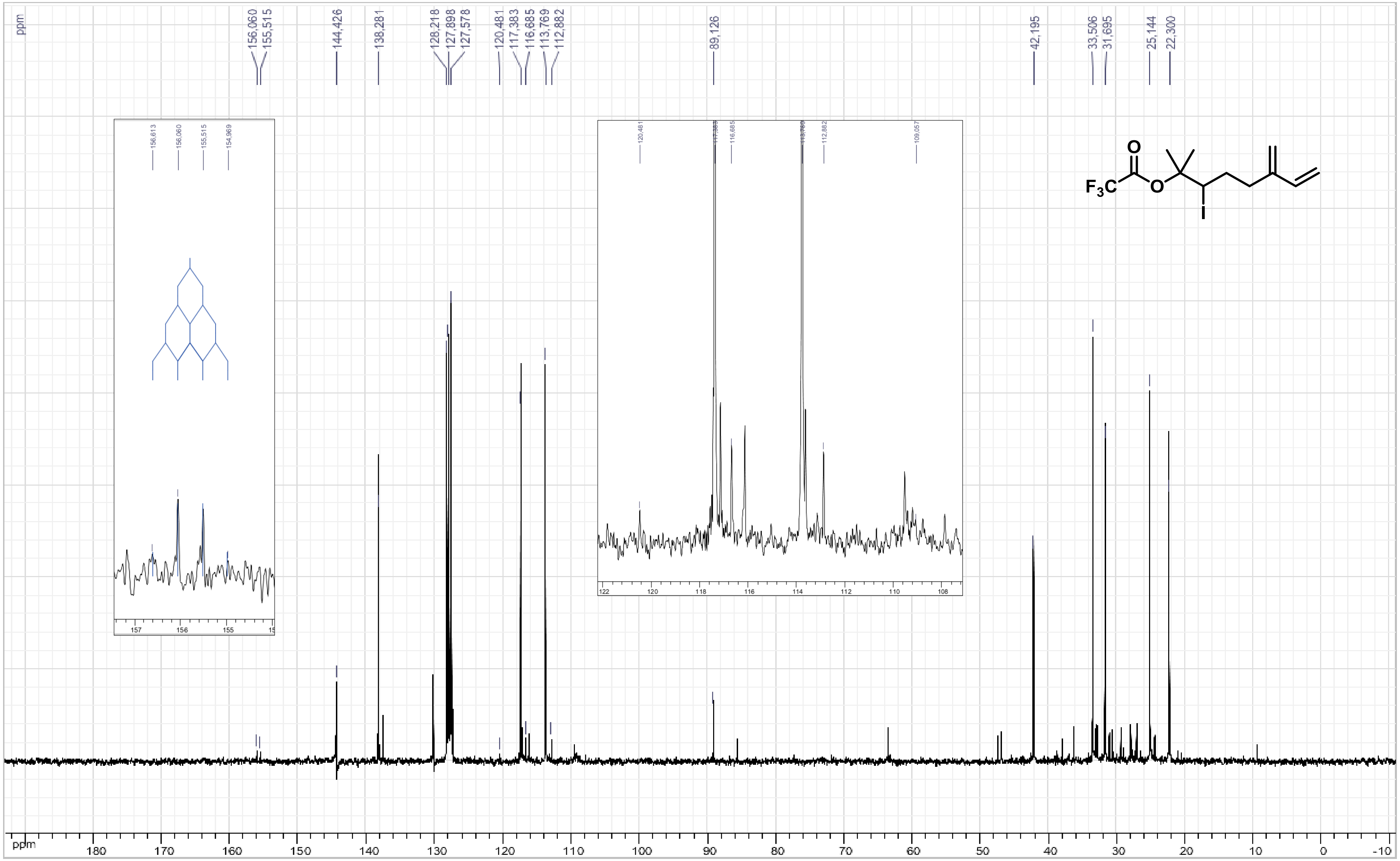
ppm
156,060
155,515
144,426
138,281
128,218
127,898
127,578
120,481
117,383
116,685
113,769
112,882
89,126
42,195
33,506
31,695
25,144
22,300
156,613
156,060
155,515
154,969
120,481
116,685
112,882
109,057
F3C
O
I
ppm
180 170 160 150 140 130 120 110 100 90 80 70 60 50 40 30 20 10 0 -10

(E)-6-Chloro-3,7-dimethylocta-2,7-dien-1-yl acetate (6a)

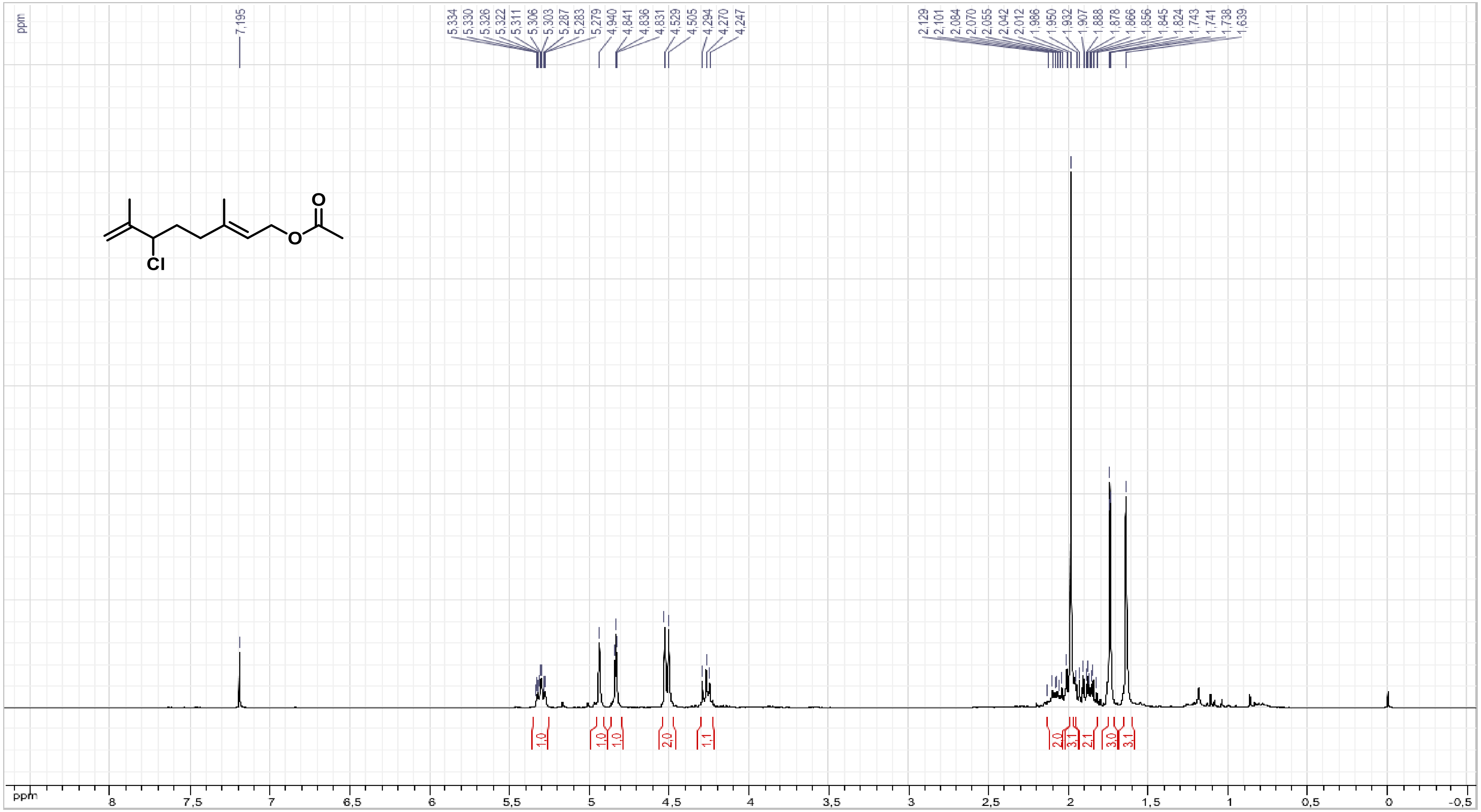

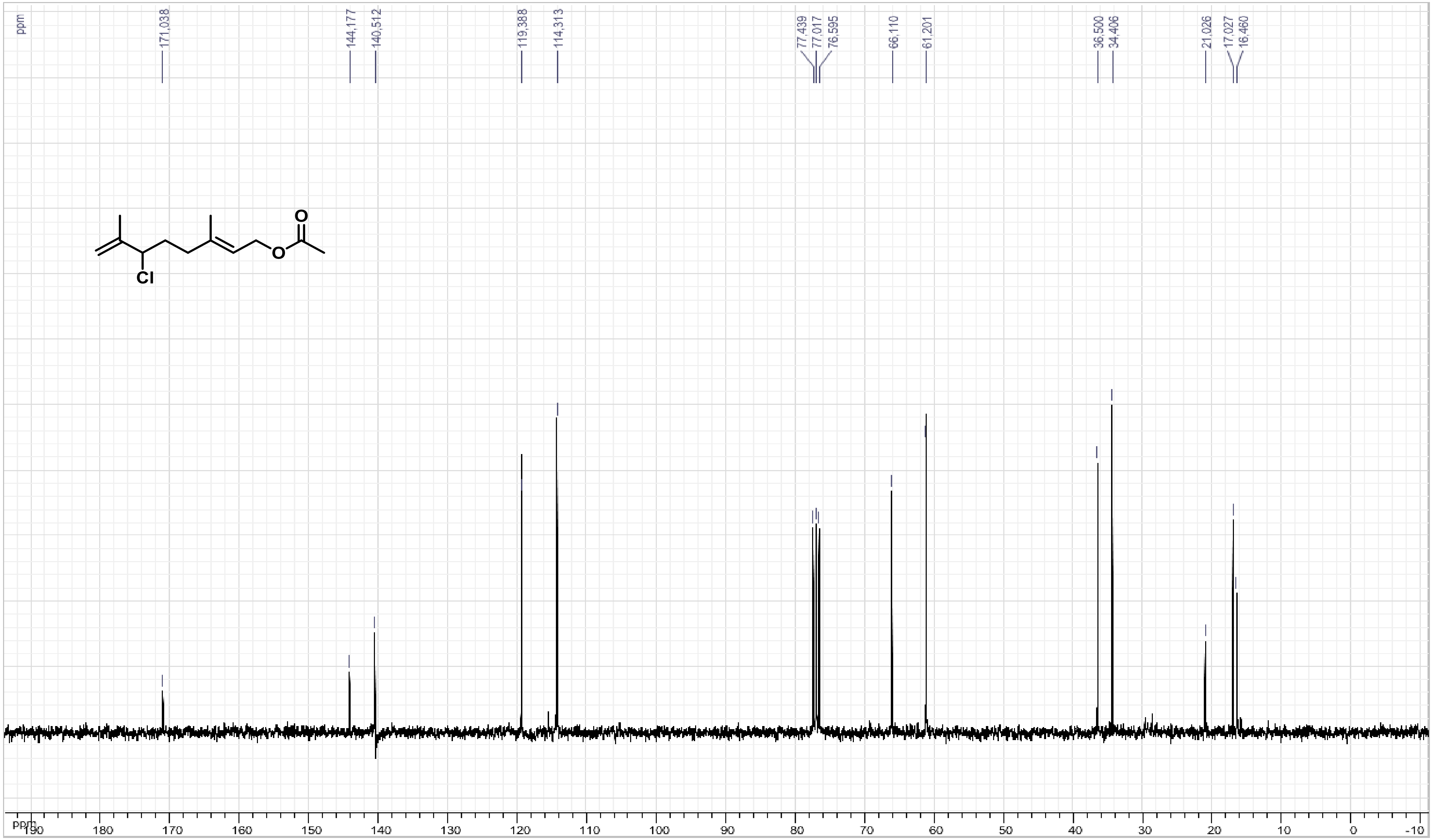
ppm
171.038
144.177
140.512
119.388
114.313
77.439
77.017
76.595
66.110
61.201
36.500
34.406
21.026
17.027
16.460
O
O
Cl
ppm 190 180 170 160 150 140 130 120 110 100 90 80 70 60 50 40 30 20 10 0 -10

(Z)-6-Chloro-3,7-dimethylocta-2,7-dien-1-yl acetate (6b)

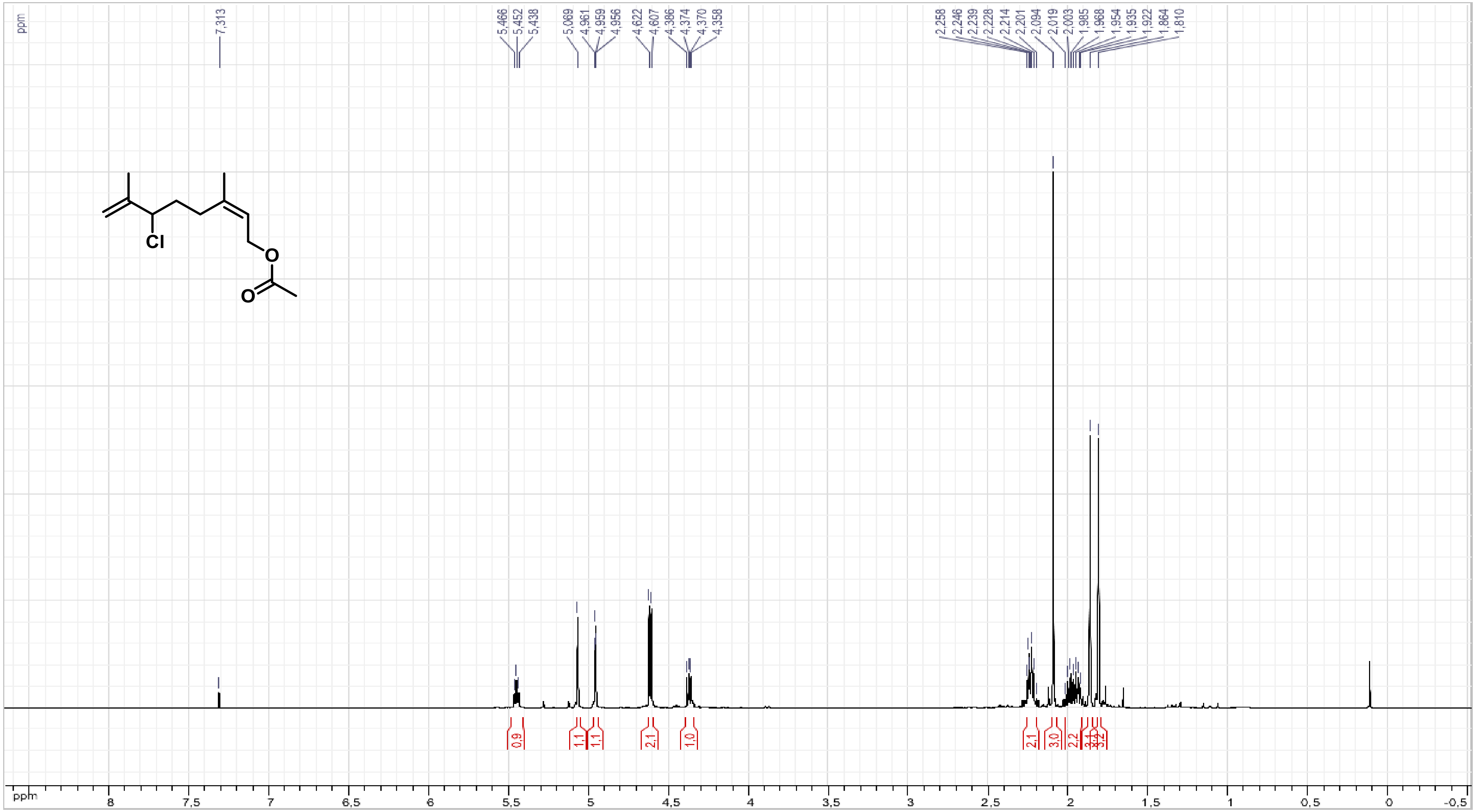

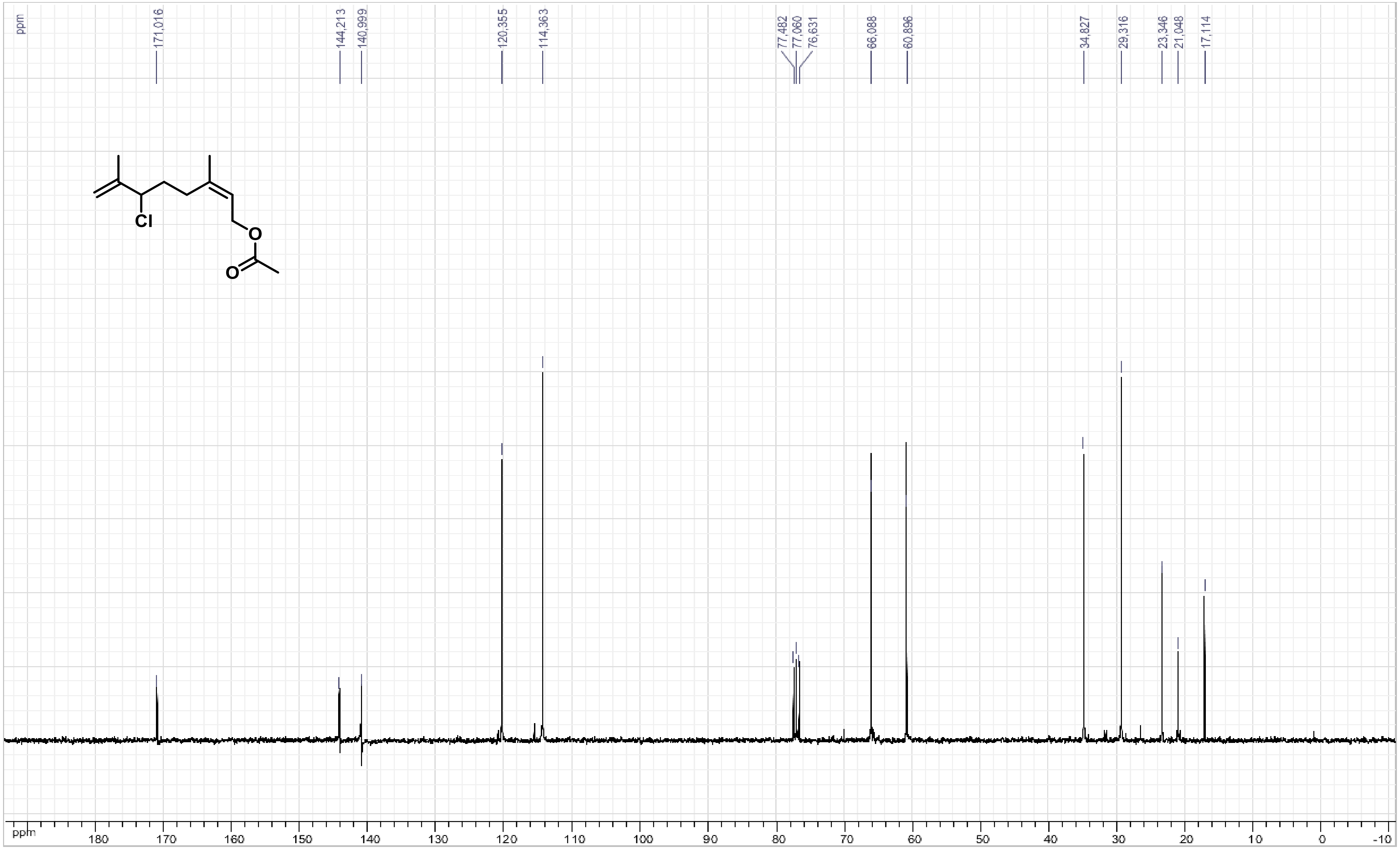
ppm
171,016
144,213
140,999
120,355
114,363
77,482
77,060
76,631
66,088
60,896
34,827
29,316
23,346
21,048
17,114
Cl
O
O
ppm
180
170
160
150
140
130
120
110
100
90
80
70
60
50
40
30
20
10
0
-10

***(E)*-6-Chloro-3,7-dimethylocta-2,7-dien-1-ol** (6c)

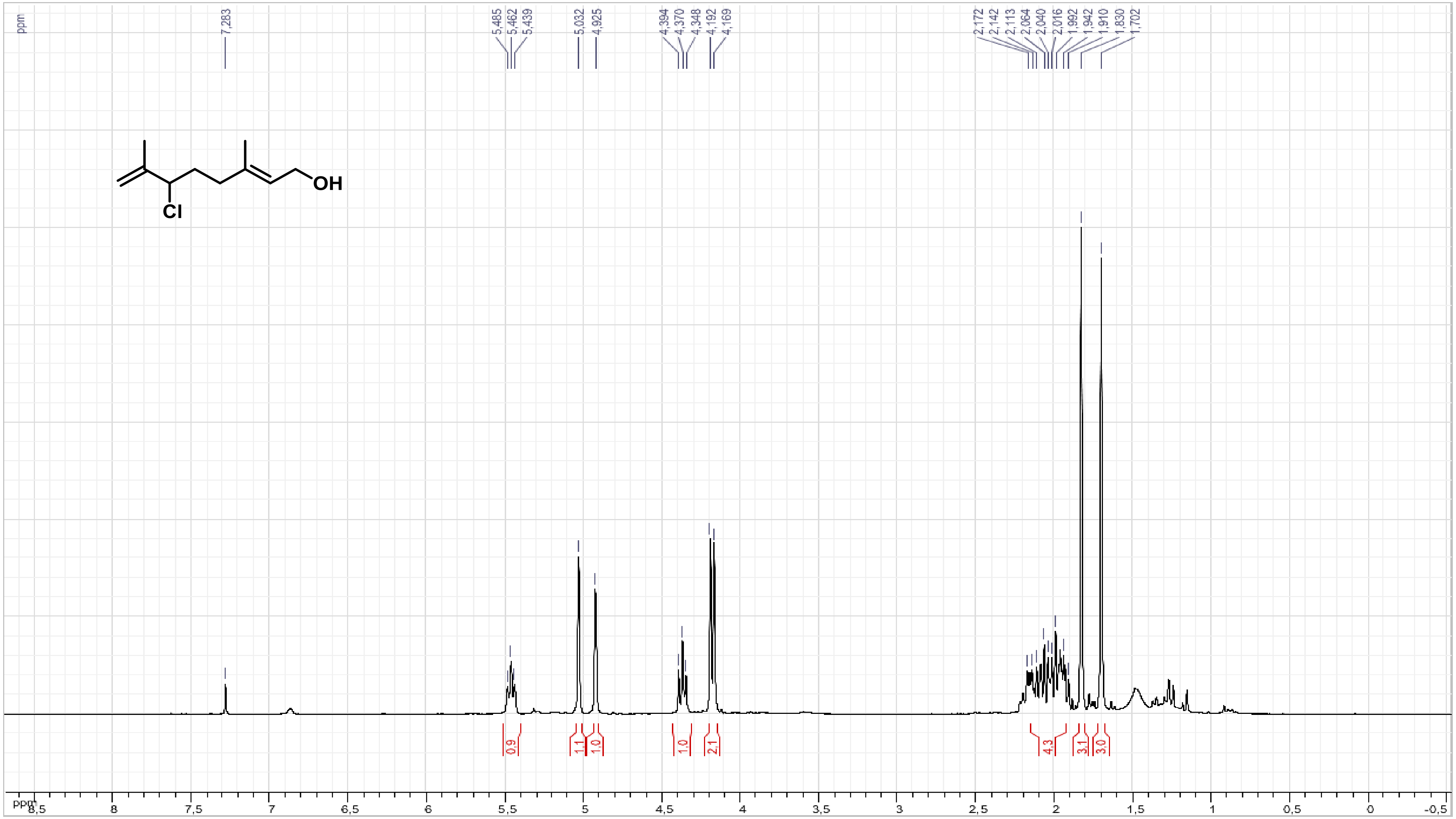

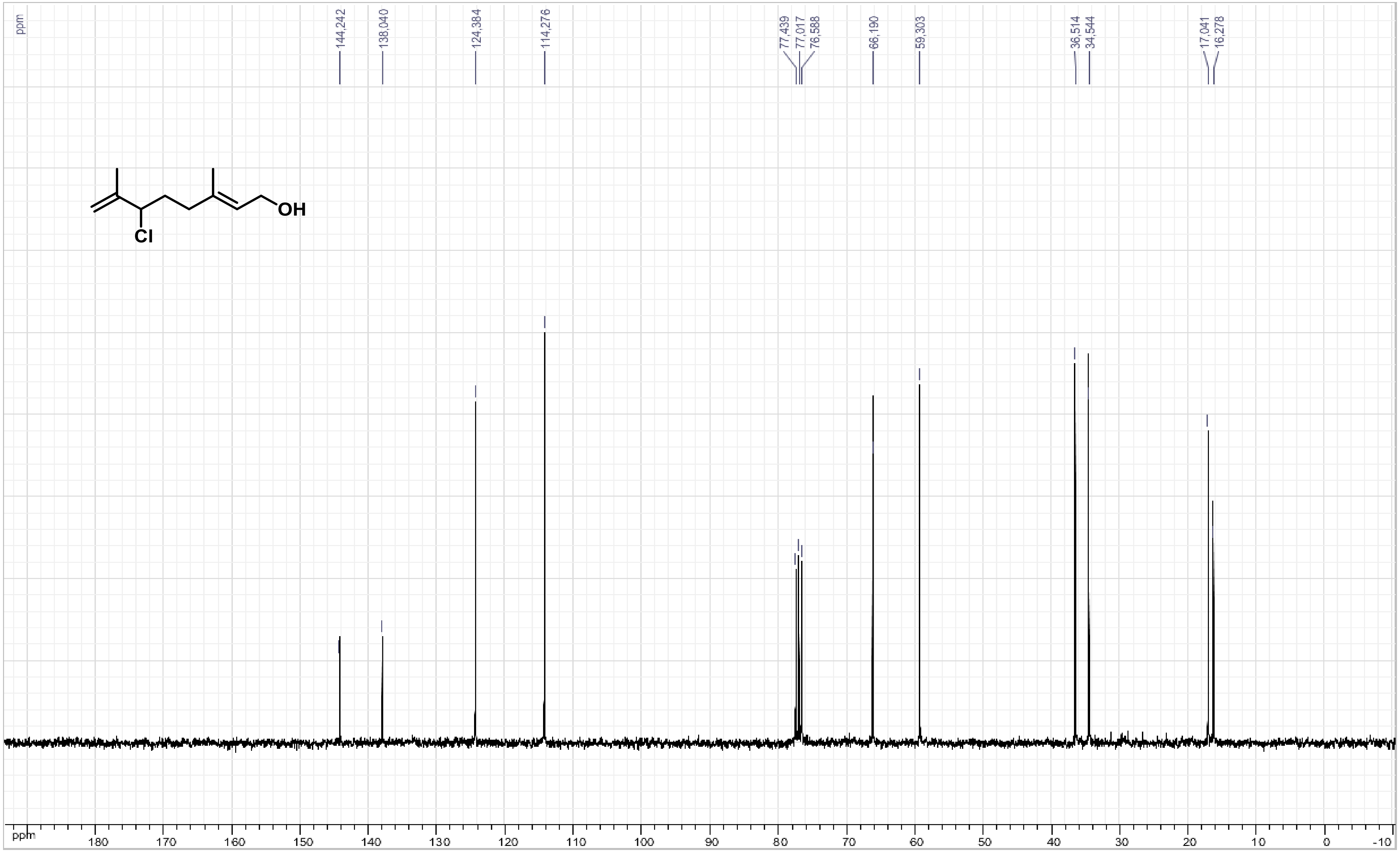
ppm
144,242
138,040
124,384
114,276
77,439
77,017
76,588
66,190
59,303
36,514
34,544
17,041
16,278
OH
Cl
ppm
180
170
160
150
140
130
120
110
100
90
80
70
60
50
40
30
20
10
0
-10

(E)-N-(6-Chloro-3,7-dimethylocta-2,7-dien-1-yl)-4-methylbenzenesulfonamide (6d)

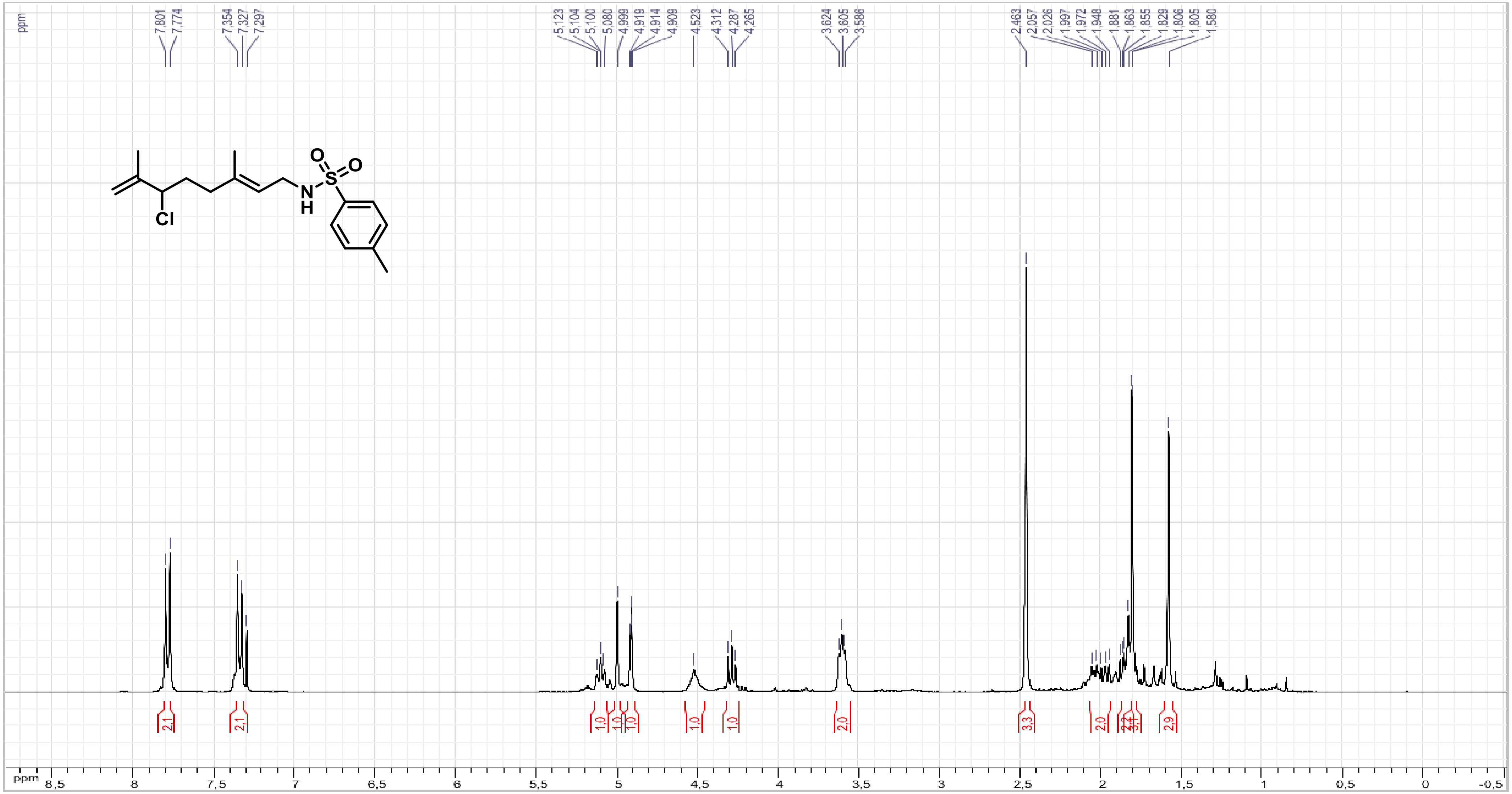

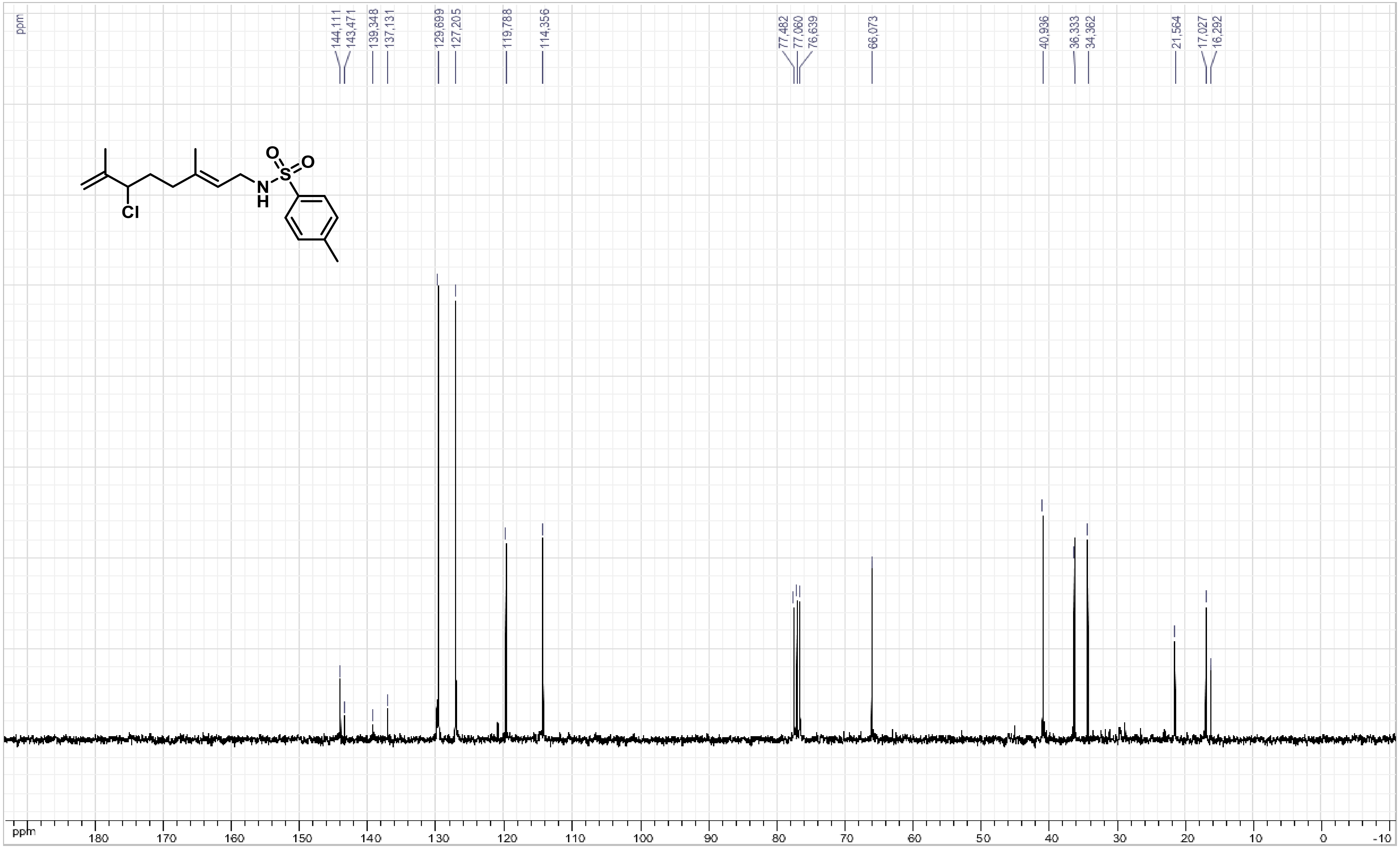

ppm
144,111
143,471
139,348
137,131
129,699
127,205
119,788
114,356
77,482
77,060
76,639
66,073
40,936
36,333
34,362
21,564
17,027
16,292
Cl
NH
O
S
O
ppm
180
170
160
150
140
130
120
110
100
90
80
70
60
50
40
30
20
10
0
-10

3-Chloro-2-methyl-6-methyleneocta-1,7-diene (6e)

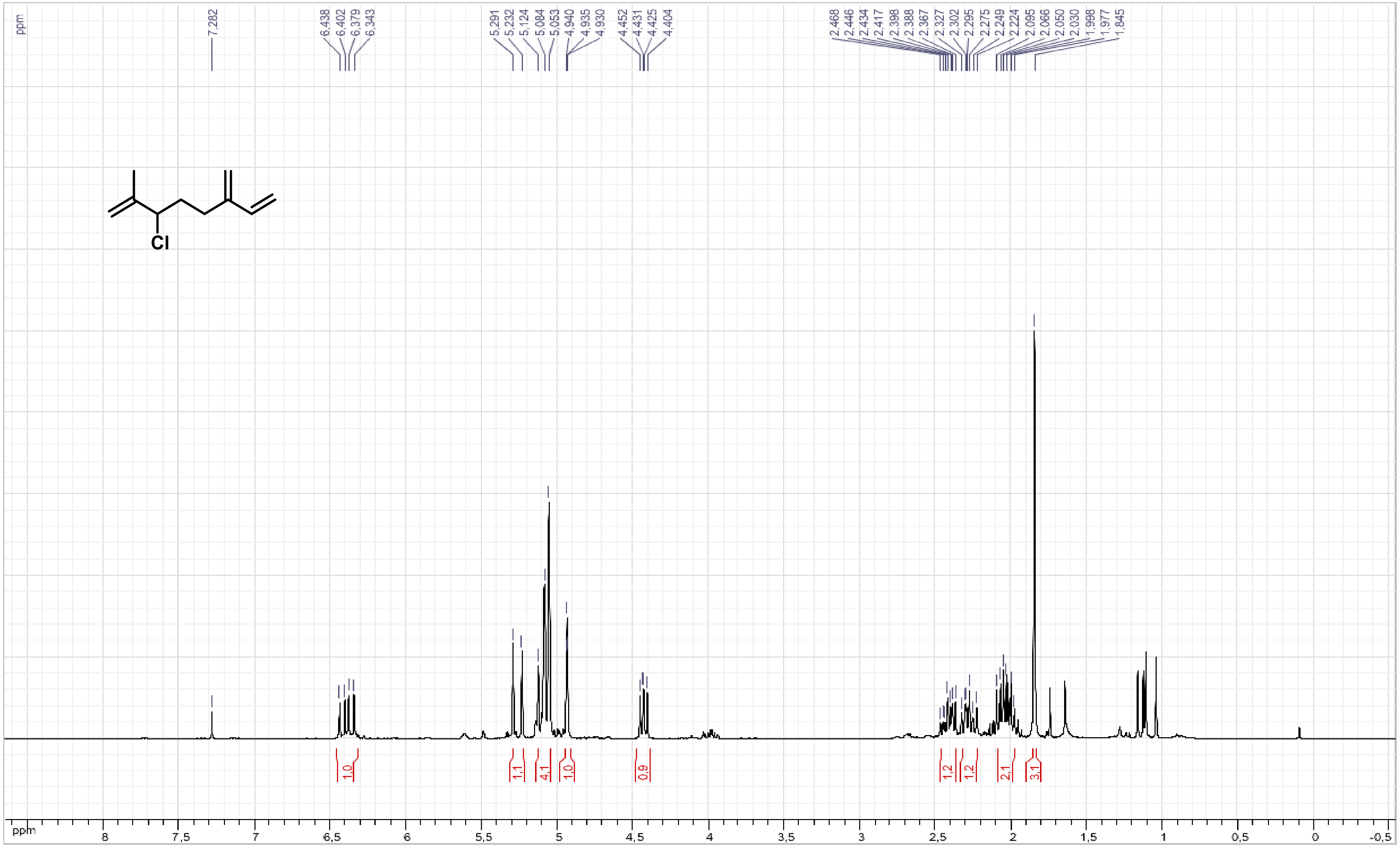

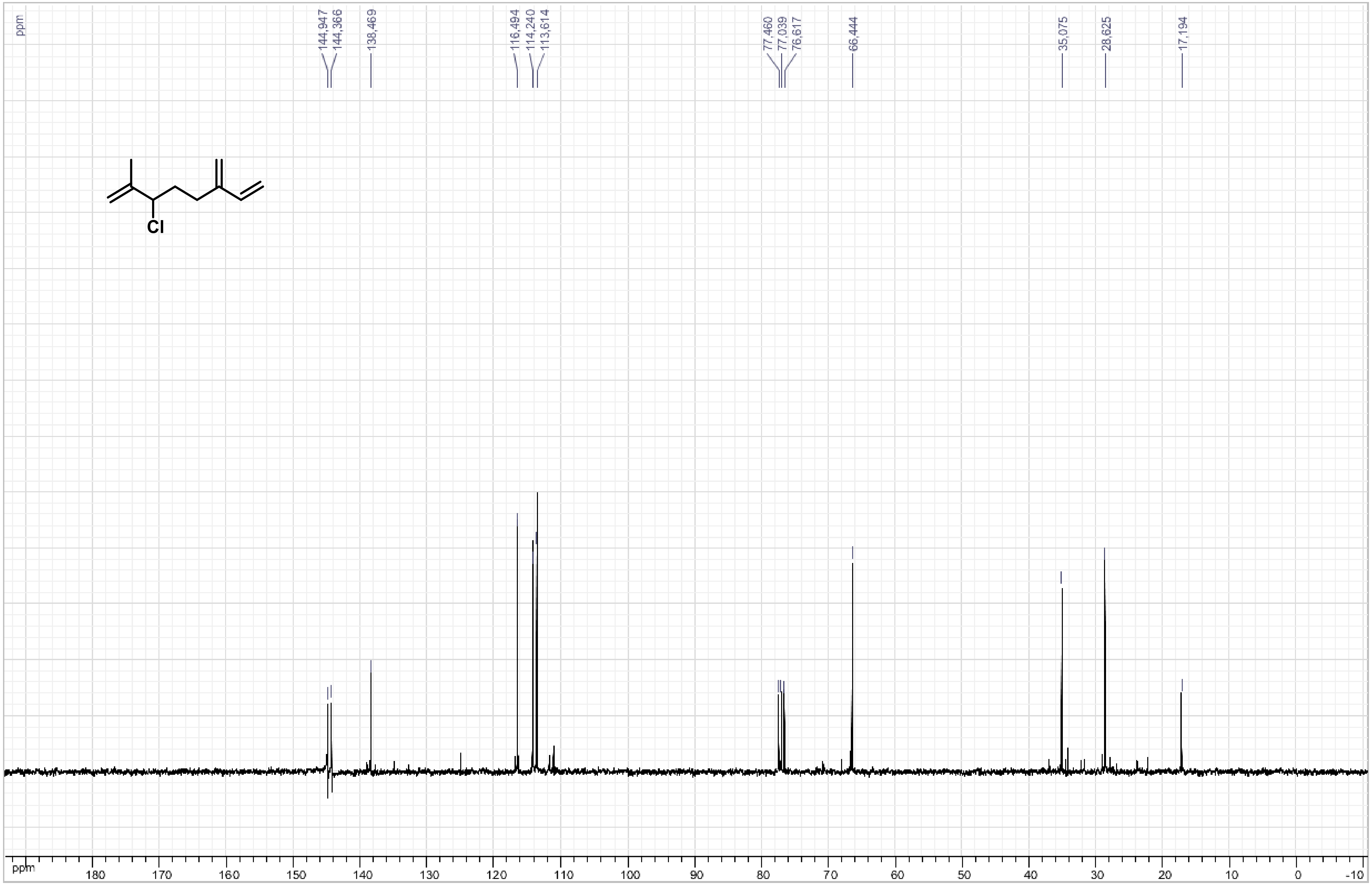
ppm
144.947
144.366
138.469
116.494
114.240
113.614
77.460
77.039
76.617
66.444
35.075
28.625
17.194
Cl
ppm
180
170
160
150
140
130
120
110
100
90
80
70
60
50
40
30
20
10
0
-10
